# The evolution of digital health technologies in cardiovascular disease research

**DOI:** 10.1038/s41746-022-00734-2

**Published:** 2023-01-03

**Authors:** Clara C. Zwack, Milad Haghani, Matthew Hollings, Ling Zhang, Sarah Gauci, Robyn Gallagher, Julie Redfern

**Affiliations:** 1 School of Health Sciences, Faculty of Medicine and Health, University of Sydney, Sydney, NSW Australia; 2 School of Civil and Environmental Engineering, University of New South Wales, Sydney, Australia; 3 Sydney Nursing School, Faculty of Medicine and Health, University of Sydney, Sydney, NSW Australia; 4 Institute for Mental and Physical Health and Clinical Translation, Deakin University, Geelong, VIC Australia

**Keywords:** Cardiovascular diseases, Medical research

## Abstract

When implemented in practice, digital technologies have shown improvements in morbidity and mortality outcomes in patients with cardiovascular disease (CVD). For scholars, research into digital technologies in cardiovascular care has been relatively recent, thus it is important to understand the history of digital health technology in cardiovascular research—its emergence, rate of growth, hot topics, and its temporal evolution. The aim of this study was to analyse more than 16,000 articles in this domain based on their scientometric indicators. Web of Science (WoS) Core Collection was accessed and searched at several levels, including titles, abstracts, keywords, authors, sources and individual articles. Analysis examined the temporal shifts in research and scholarly focus based on keywords, networks of collaboration, topical divisions in relation to digital technologies, and influential publications. Findings showed this research area is growing exponentially. Co-citation analysis revealed twenty prominent research streams and identified variation in the magnitude of activities in each stream. A recent emergence of research activities in digital technology in cardiovascular rehabilitation (CR), out-of-hospital cardiac arrest (OHCA), and arrythmia research was also demonstrated. Conversely, wearable technologies, activity tracking and electronic medical records research are now past their peak of reported research activity. With increasing amounts of novel technologies becoming available and more patients taking part in remote health care monitoring, further evaluation and research into digital technologies, including their long-term effectiveness, is needed. Furthermore, emerging technologies, which are evaluated and/or validated should be considered for implementation into clinical practice as treatment and prevention modalities for CVD.

## Introduction

Digital innovation and connection is a vital part of a modern, accessible healthcare system. Digital health is an umbrella term used to describe a range of technologies, including mobile health and applications, electronic medical records (eMRs), telehealth and telemedicine, wearable devices, robotics and artificial intelligence (AI)^1^. These technologies have a multitude of uses, including disease detection, aiding patient treatment, ensuring continuity of care, and managing a person’s health information. The 1980s were considered a rapidly progressive time for digital health technologies, with the emergence of professional organisations (e.g. American Telemedicine Association and International Medical Informatics Association) whose goals were to transition from traditional healthcare methods to more advanced, technology driven applications^2^. However, the maturation of digital health technology has occurred in the current century. Access to care and health equity is an international priority^3^, and is largely a motivating factor for health systems to move towards digital health adoption. The recent World Heart Federation Roadmap for Digital Health in Cardiology highlights the potential for digital health technologies to achieve optimal and universal health coverage (UHC) through empowering patients and providers, promoting UHC, improving long-term patient outcomes and care experience and reducing healthcare costs^4^. In the last five years (especially the COVID-19 pandemic years of 2020–2022) a new imperative has arisen to extend reach of care and achieving even higher standards of healthcare using digital health technology. Digital technology applications in healthcare have developed into a standalone market, and researchers, health professionals and industry now observe and appreciate a promising future.

Implementing best practice care for patients with cardiovascular diseases (CVDs) is one of the greatest challenges for health care providers. CVDs require frequent, continuous, and seamless management. Thus, these diseases continue to be important targets for implementation of digital technologies to enable patients, their families and communities, to manage -and improve- their health^1^ through improving quality of care, equity of access, increasing efficiencies and promoting better patient self-management^5^. CVD remains the leading cause of death worldwide with an estimated prevalence of 470 million in 2016^6^. CVD encompasses a wide range of conditions, including ischemic heart disease, cerebrovascular disease, cardiomyopathies, arrhythmias, peripheral vascular disease, rheumatic heart disease, congenital heart disease and hypertensive heart disease, among others. The majority of CVD are accelerated by largely modifiable behavioural and metabolic risks factors (e.g., smoking, physical activity, unhealthy diet), although non-modifiable risk factors (e.g., genetics, age, sex) are also important. To date, the weight of scientific evidence supports the use of digital technologies in CVD care and management^7^.

Detecting and visualizing emerging trends and transient patterns in scientific literature, as achieved by Chen’s methods^8^ is essential for developing sound research rationale and formulating relevant, useful and applicable research questions. Therefore, the aim of this study is to conduct a macro-scale analysis of the scientific literature with a broad scope on digital health innovation. Through this exploration we can grasp the breadth and depth of the knowledge of digital technology applications within the field of cardiovascular medicine.

## Results

**Fig. 1:**
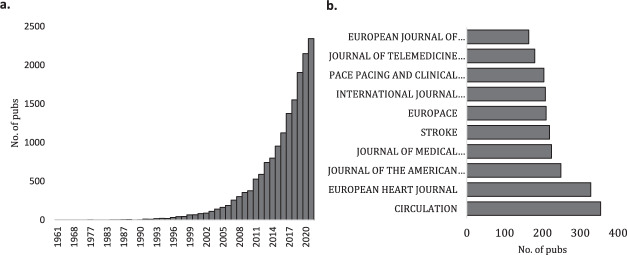
Years of publication and major sources. **a** Distribution of digital technology research articles (in cardiovascular medicine) from 1961 to 2021; **b** Major journals within the topic of digital technology applications in cardiovascular medicine.

Using the search strategy, 16,699 documents were identified in the topic of digital health technologies in cardiovascular medicine. The first article on record, published in 1961 in *Circulation* is titled ‘Electronic device for accurate identification of cardiac conduction system- its use in open-heart surgery’^9^. In the period of 30 years to the early 1990’s only 35 more relevant papers were published. Since the mid-1990’s there has been an accelerating publication trend until 2021, when over 2,300 articles were published in year (Fig. 1a). Within the observable exponential trend in this topic, there are no distinct jumps in research activity. Around 60% of the research output is in the form of original research articles (including case studies), followed by conference proceedings papers (14%), meeting abstracts (13%) and review articles (9%).

The top globally cited articles have quite distinct topics (see Supplementary Table 2). The most cited paper is “Flexible polymer transistors with high pressure sensitivity for application in electronic skin and health monitoring” authored by Schwartz, Tee^10^. The paper has received 1,337 citations in WoS provides information on the fabrication of a novel flexible pressure-sensitive sensor that has high pressure and time resolution capabilities to continuously monitor the human radial artery pulse wave. It is a step-change innovation because previously, only complex instruments such as micromanometers could read a pulse with such accuracy. Furthermore, the flexible pressure sensors developed could be used in mobile health monitoring and remote diagnostics in cardiovascular medicine. Other highly cited papers include topics about health-related behaviours such as sedentary lifestyles^11^, medication adherence^12^, robots^13^, and software^14^, which highlights the diversity within this topic.

Specific subject areas were identified using WoS *Categories*. While most of the literature is concentrated in Cardiac and Cardiovascular Systems (*n* = 4,809, 28%), it reaches across a range of sub-disciplines, including Health Care Science Services (*n* = 1,643, 10%), Engineering Biomedical (*n* = 1,449, 9%), Engineering Electrical Electronic (*n* = 1,255, 8%), Medicine General Internal (*n* = 1,201, 7%), Peripheral Vascular Disease (*n* = 1,142, 7%), Medical Informatics (*n* = 1,118, 7%), Clinical Neurosciences (*n* = 941, 6%) and Rehabilitation (*n* = 752, 4%). *Circulation* and *European Heart Journal* are the dominant outlets for publications in this area of research (Fig. 1b), with around 700 documents attributed to these two alone.

### Author contributors and networks of collaboration

Over 75,000 authors have contributed to the topic, 2,956 of whom have authored five or more articles. Authors with more than 45 contributions include Giuseppe Boriani (University-Hospital Polyclinic of Modena, Italy), Rizwan Sohail (Baylor College of Medicine, United States of America [USA]), Tiny Jaarsma (Linkoping University, Sweden), Paul Friedman (Mayo Clinic College of Medicine, USA), Bruce Wilkoff (Cleveland Clinic, USA), Larry Baddour (Mayo Clinic, USA), Jinseok Lee (Kyung Hee University, South Korea) Julie Redfern (University of Sydney, Australia) and Jenny Wang (University of Sheffield, England).

**Fig. 2:**
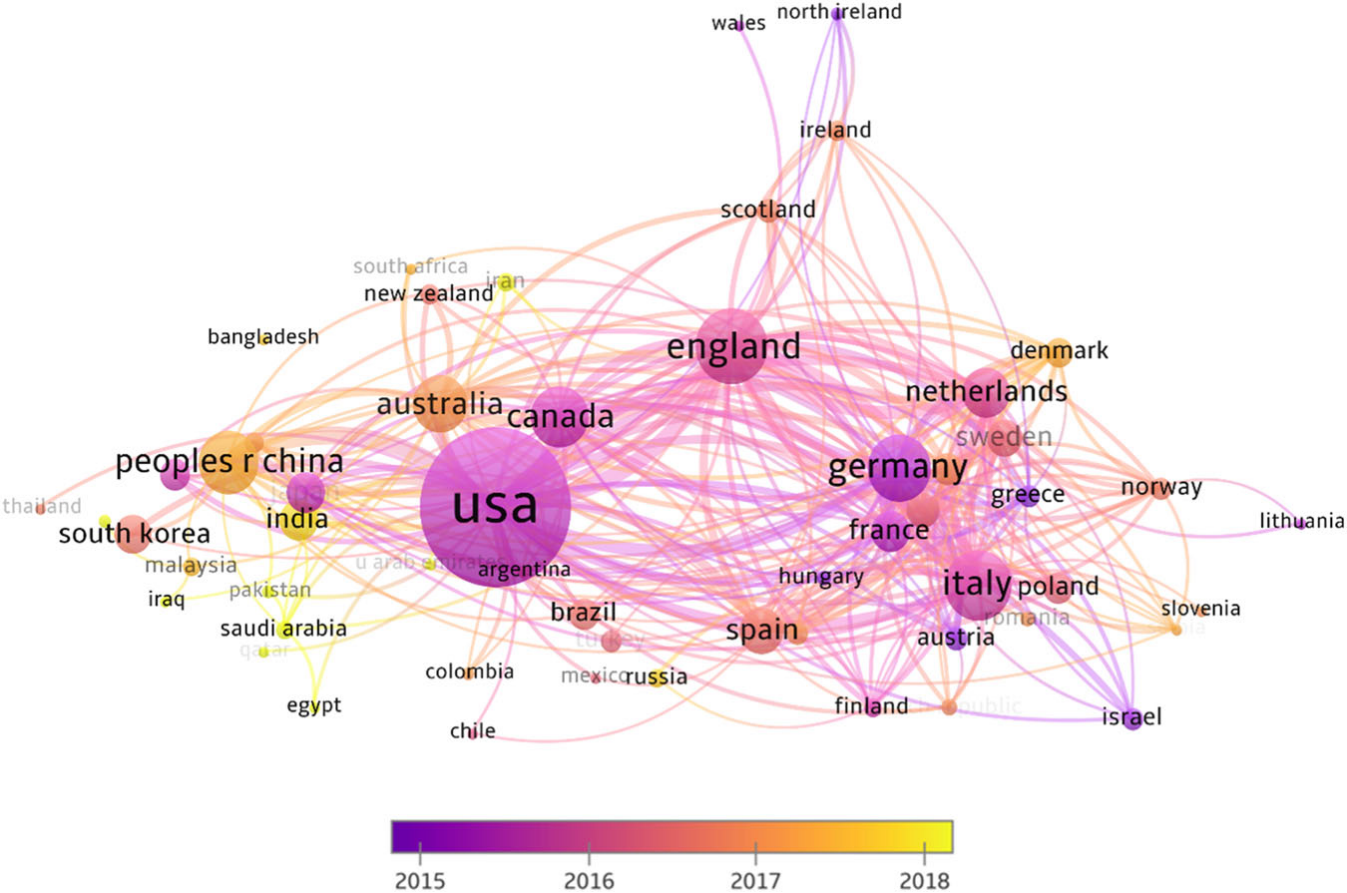
Patterns of scholarly collaborations between countries. The strength of collaborations between researchers from different countries, defined by number of documents, is indicated by the width of the link.

Publications originating from the USA have been most frequent (*n* = 5,493), followed by England (*n* = 1,494), Italy (1,338), Germany (*n* = 1,191), China (*n* = 1047), Canada (*n* = 1030), and Australia (*n* = 908) (Fig. 2). There are fewer contributions from Africa and South America (except for Brazil). Authors based in the USA are involved in all the strongest collaborations; with England (*n* = 260), Canada (*n* = 248), China *(n* = 163), Germany (*n* = 163), Australia (*n* = 159) and Italy (*n* = 146) indicating that lingual similarities and geographical proximity do not necessarily foster greater collaboration. Collaborations between researchers from English-speaking countries such as Australia, Canada, England and USA and Middle Eastern countries Iran, Iraq and Saudi Arabia are amongst the newest. Lastly, we can also observe that there are few low and middle-income countries producing scholarly research in this area.

### Semantic analysis; keywords, titles and abstracts

Table 1 shows the top keywords (see also Supplementary Fig. 1 for density of co-occurrence of keywords in the literature). In just under two years since the term emerged, Covid-19 (and alternatives e.g. sars-cov-2) have already garnered 270 mentions, reflecting the imperative of managing care when physical distancing is demanded. Keywords also provide insight into the temporal shifts in research and scholarly focus. Table 1 also shows the top keywords with the strongest bursts of citation and the keywords with the most recent and strongest bursts of citation. Children and infants, men, youth, and elderly patients are the populations of interest for scholars, as shown by bursts. Children appeared to be a strong research interest for over two decades (1991-2013), in parallel with research topics such as blood pressure, cardiovascular reactivity and family history.

**Table 1:** Top keywords, top keywords bursts of citations and recent keyword bursts of citation.

	Top keywords	n	Top keyword burst	Start-end yr	Strength	Recent keyword burst	Start-end yr
1	telemedicine	1065	randomized trial	2005–2012	17.09	neural network	2018–2021
2	stroke	984	children	1991–2013	16.83	PPG/photoplethysmography	2018–2021
3	heart failure	970	response	1991–2007	15.69	fibrillation	2018–2021
4	Virtual Reality	434	cardiovascular reactivity	1991–2007	14.07	ischemic stroke	2018–2021
5	pacemaker	425	disease management	2006–2012	13.81	mhealth	2017–2021
6	remote monitoring	380	system	2005–2011	13.73	thrombectomy	2017–2021
7	mhealth	336	blood pressure	1991–2004	13.64	IoT	2017–2021
-	Covid-19	270					

In the early 1990s, when digital health innovations were rapidly accelerating in cardiovascular medicine research in the early 1990s, the keyword bursts were generally associated with cardiovascular risk factors (e.g. blood pressure, exercise). More recently, bursts of activity are related to modern terminology affiliated with complex data and sensors, including ‘IoT’ (internet of things), ‘neural network’ and ‘photoplethysmography’.

### Major divisions of digital technologies in cardiovascular research

**Fig. 3:**
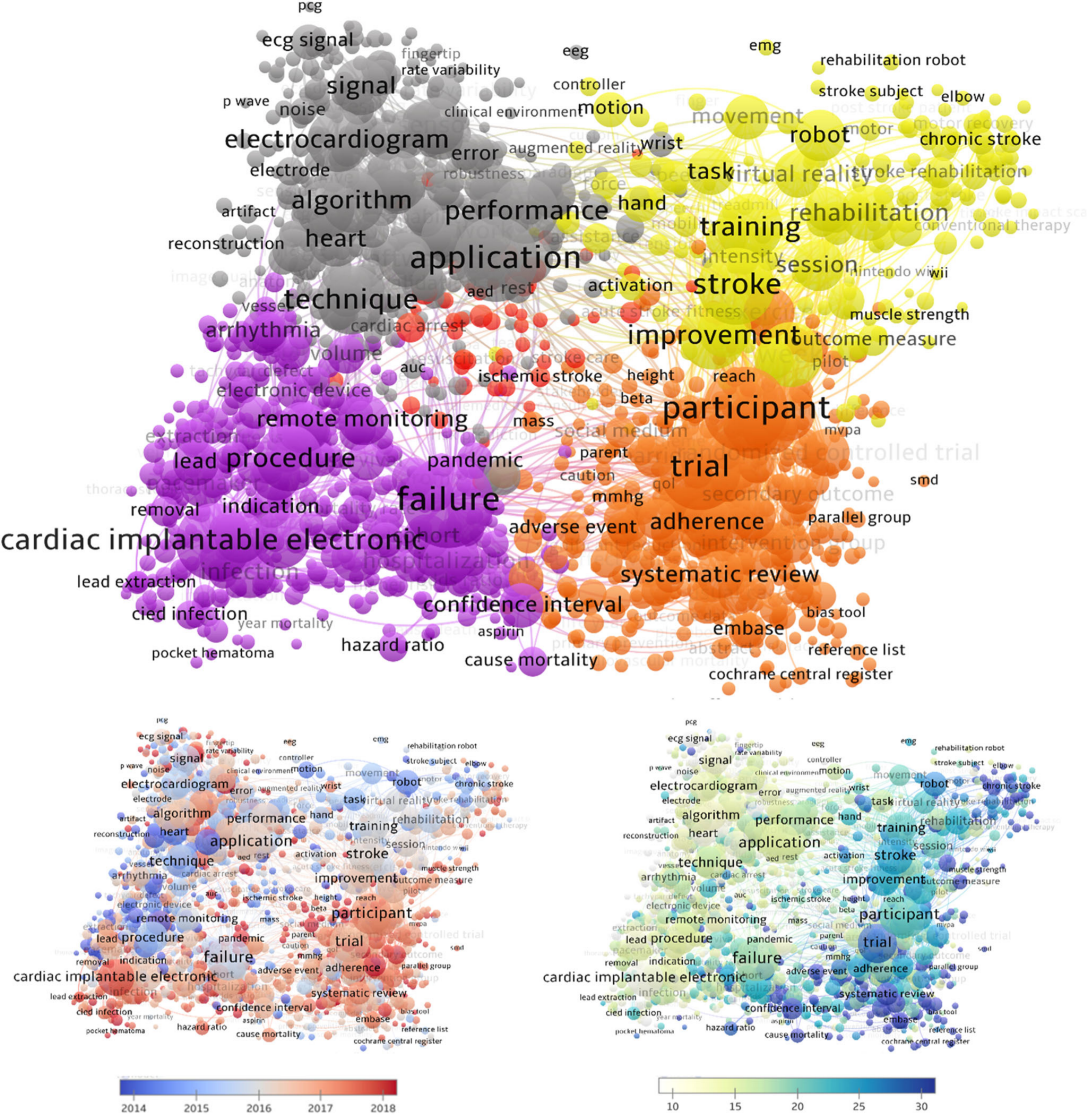
Network-view map of term co-occurrence in titles and abstracts of articles within digital health technologies in cardiovascular medicine. Bottom left: map of average publication year; Bottom right: map of average number of citations. An interactive online map is available here: https://app.vosviewer.com/?json=https://drive.google.com/uc?id=1wo3ylYehGisHVX0CLM49voNitTjKsYOW.

Additional insight into topic structure of the topic was found by completing an analysis of the occurrence of terms in the title and abstracts of articles. Figure 3 shows the frequency of the co-occurrence of terms and is complimented by the averaged publication year of articles containing these terms and the average number of citations articles with these terms receive. The analysis revealed that there are five major divisions. Table 2 shows the top terms, most cited terms, and youngest terms for each cluster.

**Table 2:** Research areas with applications of digital health technology from co-occurrence of terms in titles and abstracts of articles.

Cluster ID (colour)	No. of items	Descriptor	Top terms	Youngest terms	Most cited terms
1 (purple)	491	Cardiac electronic implantable devices & electrophysiology	Cardiac implantable electronic device (CIED), remote monitoring, procedure, failure, infection, complication, implantation, incidence, surgery, pacemaker, lead, video, atrial fibrillation, arrythmia, hospitalization	CIED, pocket hematoma, transvenous lead extraction, major complication, catheter ablation, radiotherapy, leadless pacemaker, transthoracic echocardiology, implantable loop recorder, atrial high-rate episode, envelope, lockdown, sars cov, pandemic	European Heart Rhythm Association, cardiac resynchronisation therapy, hospital mortality
2 (orange)	474	1. Mobile health technology for secondary prevention of CVD 2. Clinical trials	1. Physical activity, body mass index, diabetes, barrier, cardiac rehabilitation, coronary heart disease 2. Intervention group, trial & randomised controlled trial, participant, adherence, primary outcomes, secondary outcome, control group, baseline, usual care Terms related to databases and study design: systematic review, meta-analysis, Embase,	Medication adherence, cardiometabolic risk factor, mhealth, healthy lifestyle, text message, engagement, step count, social medium, promotion, fitbit, activity tracker, apps, ehealth, ethics, vulnerable population, Terms related to databases and study design: primary outcome, secondary outcome, PubMed, heterogeneity, Scopus, trial registration, qualitative study, quantitative study	Confidence interval, cause mortality, systematic review, usual care, metanalysis, Embase, adverse event, bias, data collection, intention, randomised controlled trial, Cochrane library, CINAHL, intake, fitness, oxygen consumption, video game
3 (grey)	441	Wearable technologies	Sensor, algorithm, application, technique, performance, heart rate, environment, accuracy, signal, wearable device, work, feature, detection, software, internet, electrocardiogram, sensitivity, doctor, real-time, capability, validation, requirement, image, error, heart rate variability	Photoplethysmography (ppg), IoT, wearable device & warble technology, smartphone camera, ppg signal, neural network, support vector machine, cloud, machine learning, artificial intelligence, apple watch, smartwatch, bpm (beats per minute), deep learning, scalability, decision tree	Skin, frequency domain, power consumption, property, RAT, response time
4 (yellow)	195	Technology applications in stroke rehabilitation	Rehabilitation, stroke, improvement & significant improvement, week, training, virtual reality, robot, task, movement, motion, hand, arm, stroke patient, intensity impairment, speed, force, recovery	Exergame, gamification, immersive virtual reality, cognitive impairment.	Computer game, arm, outcome measure, strength, cognitive function, muscle strength, motor impairment, daily living & ADL, upper limb, Fugl Meyer assessment, robotic device, robot-assisted therapy, functional recovery, hemiparesis, velocity, player
5 (red)	57	Emergency cardiovascular care	Cardiopulmonary resuscitation (CPR), cardiac arrest, emergency, acute stroke	Hospital cardiac arrest, Out-of-hospital cardiac arrest (OHCA)	Telestroke

Cluster 1 (Fig. 3; purple, 491 items) focusses on *Cardiac electronic implantable devices* (CIEDs), including pacemakers, implantable cardioverter-defibrillators and other devices used to help control of monitor heartbeats in individuals with health rhythm disorders and heart failure (HF).

Cluster 2 (orange, 474 items) represents the area of *Mobile health technology for secondary prevention of CVD*. This includes methods of measuring risk factors and modes for intervention designed to affect the actions that individuals take regarding their health. This division is saturated with terms such as physical activity, healthy lifestyle, Fitbit and step count, and is associated with clinical trials.

Cluster 3 (grey, 441 items) consists of terms related to *Wearable technologies*. A large focus of this clusters is using photoplethysmography – or sensor-technology to measure physiological parameters.

The last of the major divisions, Cluster 4 (yellow, 195 items) is concerning *Technology applications in stroke rehabilitation*. Possible interventions for post-stroke rehabilitation have included use of immersive technologies and gamification such as Virtual Reality and robotics for increasing movement.

Using the interactive link to the visual representation (see caption of Fig. 3), it is determined that some of the newest terms in Cluster 1 are related to the Covid-19 pandemic (‘lockdown’, ‘pandemic’, ‘sars cov’). Throughout the clusters, there is reduced occurrence of terms related to minority populations such as ‘disability’, ‘rural’ and ‘culturally diverse’.

The smaller division, Cluster 5 (red, 57 items) consists of terms related to *Emergency cardiovascular care*. The care and management of out-of-hospital cardiac arrest (OHCA) is evolving and is strongly influenced by emerging digital technologies. Examples include wearable life detection technologies to improve survival, drones delivering automated defibrillators to the scene of the arrest, and advancements in mobile and digital technology used to leverage bystander response^15^.

### Author collaboration

**Fig. 4:**
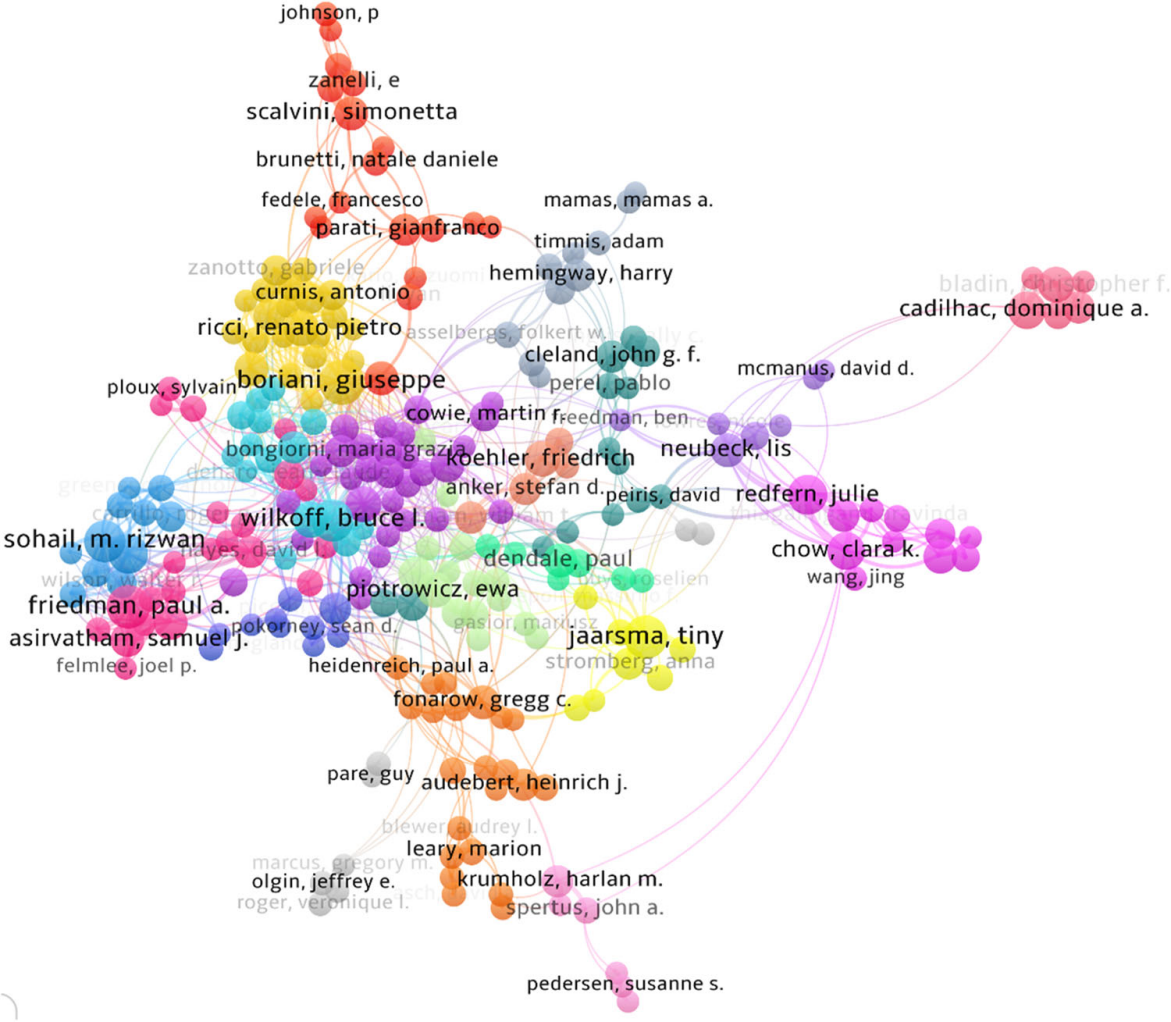
Clusters of author collaboration by total link strength. Co-authorship link strength is used to provide an indication of how many publications two researchers have co-authored. Interactive map available here: https://app.vosviewer.com/?json=https://drive.google.com/uc?id=129YuMxn-9sn-ht5mCd88-ADpbh6nX0v5.

Examining collaboration, Fig. 4 shows clusters of authors who frequently work together to produce scholarly research. This figure does not represent all 75,000 authors who have contributed to this topic overall, only those who have contributed to the central network of research (see Supplementary Table 1 for details of each cluster). There are ten clusters with ten or more authors.

Clusters 1, 2, 3, 6, and 10 are groups of scholars researching cardiac electrophysiology. However, there are some minor variations in research topic, for example, clusters 3 and 6 is focused on cardiac resynchronisation therapy, whilst clusters 2 and 6 are looking at lead extraction and management, and cluster 10 is associated with research in infections associated CIEDs. Cluster 1 is very homogenous and based in Western Europe, with few exceptions. Similarly, Cluster 6 is based in North America, with several authors affiliated with Cleveland Clinic. Cluster 5, another group researching thromboembolic diseases and telecardiology, is exclusive to Italian affiliations. Aside from topics related to CIEDs, four of the other major collaborative groups are researching cardiopulmonary resuscitation (cluster #4), telemonitoring in HF and stroke prevention (cluster #7), mHealth and telemonitoring in HF (cluster #8) and secondary prevention of coronary heart disease (CHD) (cluster #9).

The strongest links within this central author network are between Ewa Piotrowicz and Ryszard Piotrowicz (cluster 7, link strength = 23.73), Friedrich Koehler and Kerstin Koehler (cluster 18, 13.50), and Clara Chow and Julie Redfern (cluster 9, 13.78). Almost all the strongest links are between authors in the same cluster, except for frequent collaboration between Julie Redfern and Lis Neubeck (cluster 9 and 15 respectively, link strength = 7.33), which links two groups of multidisciplinary clinician researchers who are focussed on secondary prevention of CHD and CR.

### Influential references of digital technology and cardiovascular science literature

Influential articles relevant to the specific topic, ranked by local citation count, are listed in Supplementary Table 3. This metric considers the reference lists of documents in the overall dataset and how many citations the references receive. Based on this metric, ‘Telemonitoring in Patients with Heart Failure’^16^ is the most influential study in this field. The study indicated that the application of telemonitoring for patients recently hospitalized for HF did not improve outcomes. The other two most influential papers within this topic are ‘2016 ESC Guidelines for the diagnosis and treatment of acute and chronic heart failure’^17^ and ‘Update on Cardiovascular Implantable Electronic Device Infections and Their Management: A Scientific Statement From the American Heart Association’^18^. The last of which provides an important update on recommendations for the care of patients with infections due to CIEDs, as well as highlighting the research gaps. Studies, reviews, and guidelines focussed on HF, telemonitoring and CIEDs dominate the list of top ten locally cited articles.

Additionally, we can observe the articles that have had the strongest burst of citations since publication (see Supplementary Table 4). The subjects of all of articles with the strongest bursts are related to telemonitoring or remote monitoring. The top two are ‘Noninvasive home telemonitoring for patients with heart failure at high risk of recurrent admission and death: the Trans-European Network-Home-Care Management System (TEN-HMS) study’ (strength = 36.55)^19^ and ‘Structured telephone support or telemonitoring programmes for patients with chronic heart failure’ (strength = 31.81)^20^.

### Temporal evolution of digital health technologies in cardiovascular research

**Fig. 5:**
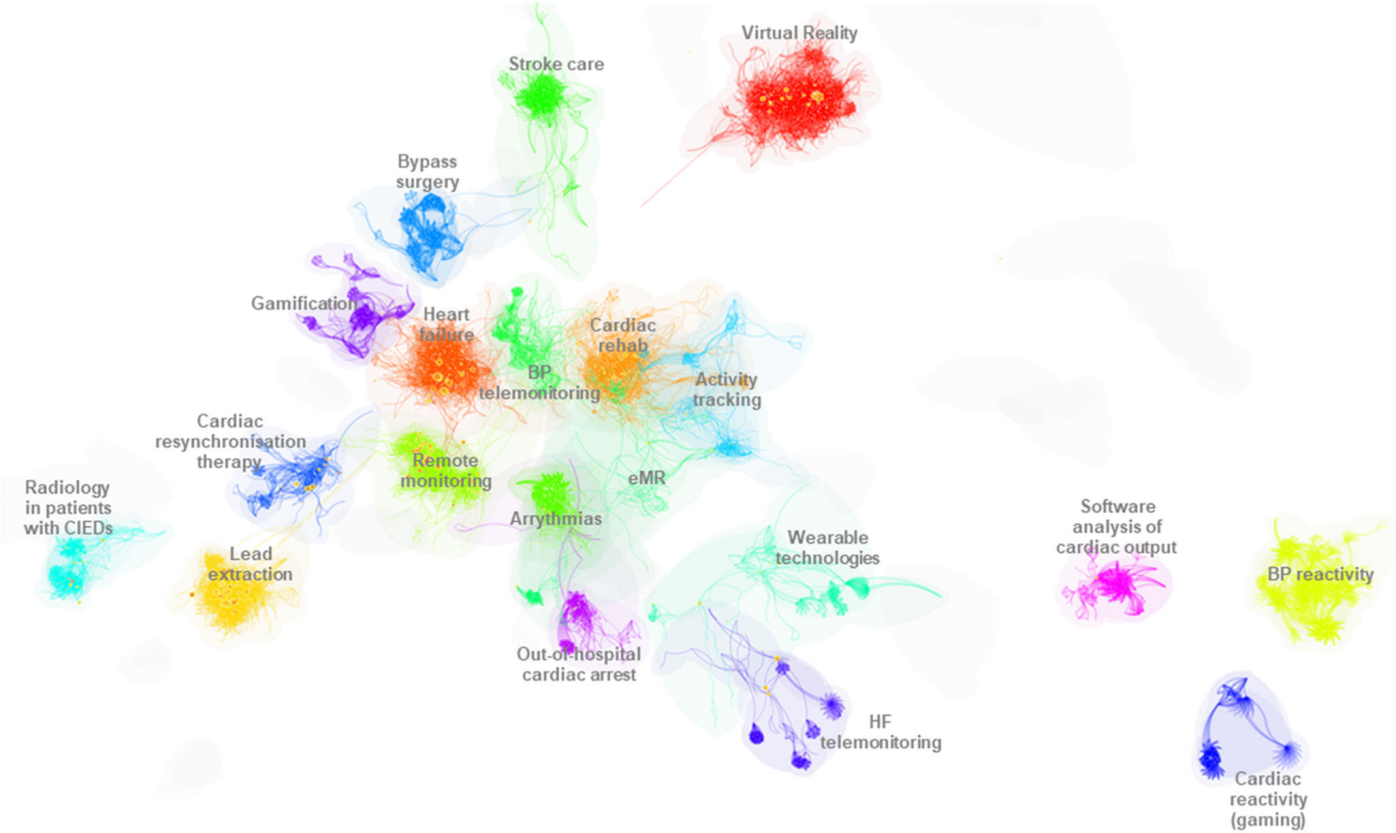
Birds-eye view of the research areas that digital technologies have been applied in cardiovascular medicine. A dynamic visualisation of the interplay between the research areas (from 1990 to 2021) is accessible via this link Temporal map_digital health_CVD.mp4.

The growth and temporal evolution of this topic was visualized using document co-citation methodology. Figure 5 shows a birds-eye view of the twenty major divisions - or *research streams* - in which digital technologies have been applied in cardiovascular research (Supplementary Fig. 2 shows the research streams in a timeline format).

**Fig. 6:**
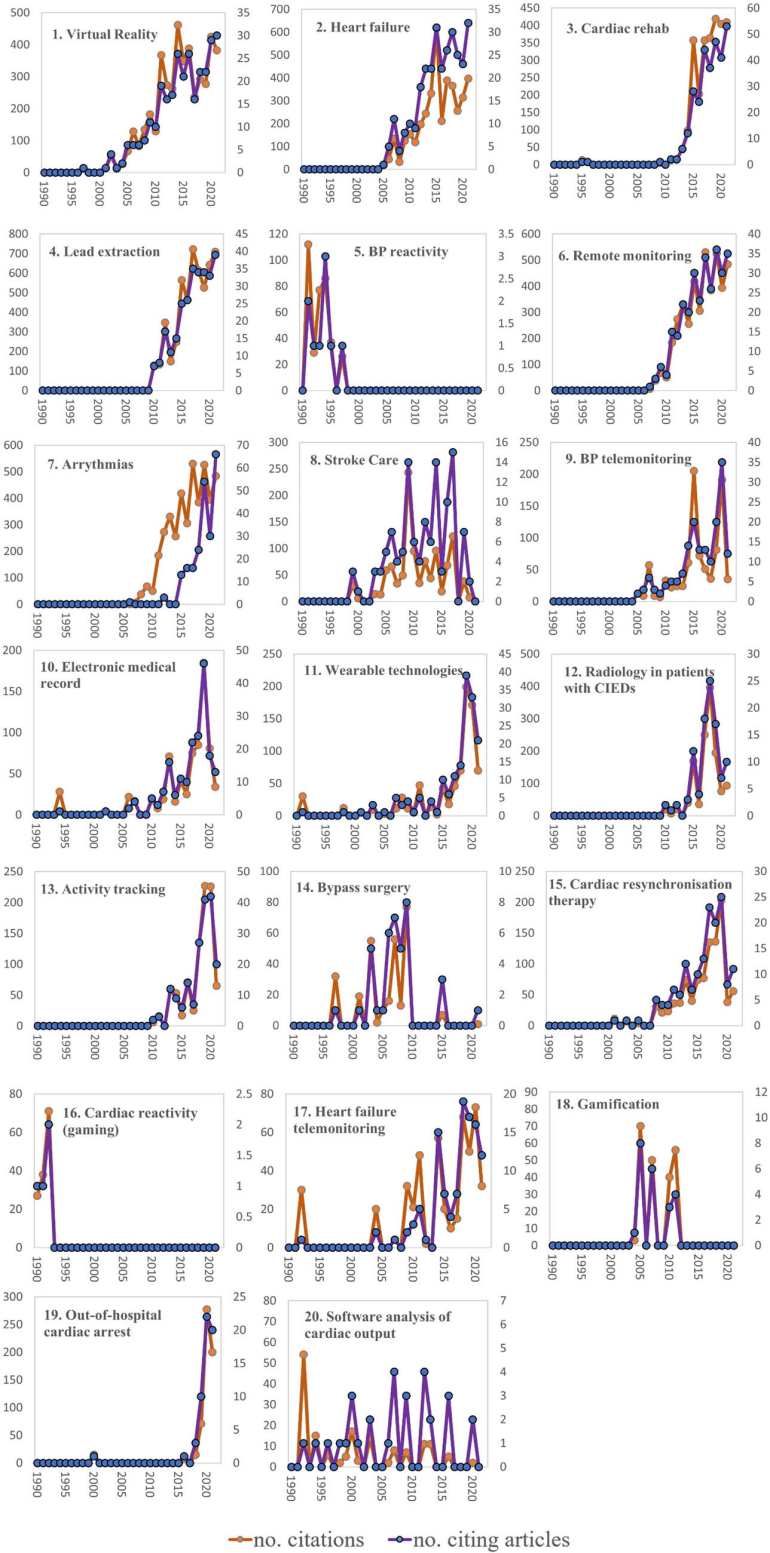
Number of citations (orange datapoints) and number of citing articles (blue datapoints) for each cluster. Left axis: total number of citations; Right axis: total number of citing articles. Note: scale is different for each cluster.

Figure 6 provides a visual representation of the extent of research activity within each stream of digital health applications within cardiovascular medicine (1990-2021) (see Supplementary Fig. 3 for deeper insight into the twenty research streams). It is evident that within the major streams, blood pressure reactivity, bypass surgery, cardiac reactivity to gaming and gamification are no longer active research areas in digital health applications. We can clearly see that there are emerging and heightened research activities in the areas of cardiac rehabilitation and out-of-hospital cardiac arrest. Research in remote monitoring, Virtual Reality, HF, transvenous lead extraction, remote monitoring, and arrythmias emerged between 2000-2010 and have maintained a high level of scholarly interest since. Research into the application of wearables (smartwatches, body sensors etc.) and activity tracking for cardiovascular care, and eMRs, appeared to peak between 2015-2020 but are now showing signs of slowing down. The oldest clusters, as determined by the mean year of article publication are *Cardiac reactivity* (1981), and similarly *Blood pressure reactivity* (1984), and *Software analysis of cardiac output* (1989). The average year the citing articles are 1991, 1993 and 2007 respectively, showing that there is little or no modern research being conducted in these areas. Contrastingly, looking at research activity we can see that the most modern topics are *Cardiac rehabilitation* (mean citing year 2018), *Activity tracking* (2018), *Radiology in patients with CIEDs*, (2018), *Arrythmias* (2019) and *Out-of-hospital cardiac arrest* (2020).

*Cardiac rehab*i*litation* is an interesting cluster in that it has some of oldest articles, dating from 1954, but it still a modern-day research area. The earlier papers from mid-1970’s-1980’s have a focus on behavioural change and self-efficacy^21,22^.

A paper by Chow, Redfern^23^, titled ‘Effect of Lifestyle-Focused Text Messaging on Risk Factor Modification in Patients With Coronary Heart Disease A Randomized Clinical Trial’ has received the most local citations (*n* = 131, and globally *n* = 554) as well as having the strongest burst of citation (13.05) between 2017-2021. Chow, Redfern^23^, whose focus is secondary prevention of CHD, shows that using a text-message intervention may improve cardiovascular risk factors such as blood lipids, physical activity, blood pressure, BMI and tobacco intake; a clear example of a simple digital health application for improving cardiovascular care outcomes. Lastly, research related to HF, CIEDs and remote monitoring make an appearance in more than half of the research.

## Discussion

For scholars, research into digital health technologies in cardiovascular care has been relatively recent, thus it is important to understand the history of digital health technology in cardiovascular research—when it emerged, the rate of growth, hot topics, and its temporal evolution—to ensure clarity and direction when formulating research questions. Previous studies using a similar method have provided insight into the evolution of digital medicine and digital health research^24,25^. Other studies, including a systematic review have shown how digital health interventions can have a positive effect on CVD outcomes^26^. However, no other paper has completed a macro-scale analysis of the relevant research. The results of this analysis will help inform scholars of any significant developments in the research area, identify gaps in the literature, and make informed predictions about the future directions of the topic.

One notable omission relevant to digital health applications in cardiovascular care is the absence of no major or frequent keywords associated with *co-design research* (e.g ‘patient-centred’, ‘user’, ‘consumer-led’). Patient-centered co-designs are important in terms of implementation factor so researchers can see how new devices are accepted and effectively used by patients in a home-based environment^27^. In cardiovascular research, large-scale and long-term user involvement through a co-design process, can support adaption and lead of quicker adoption of digital health technologies^28^. Research groups are working and publishing in the human-centered design space^29^, thus, terminology related to co-design frameworks will likely emerge should this topic be re-analysed in the near future. On the other hand, with the global burden of CVD showing no signs of slowing, it is promising to see the frequency of clinical trials utilised in secondary prevention of CVD. Data from clinical trials are consistently proving the efficacy of interventions in reducing the risk of cardiovascular events in secondary prevention patients^30^, however, there are still issues with poor adherence to therapies^31^.

Researchers from North America, Australia, and parts of Western Europe appear to be most active in this area with strong focus on CIEDs and secondary prevention of CVD. There are multiple author networks whose work focusses solely on CIEDs and electrophysiology. It could be argued that this is one of the most influential digital technologies in contemporary cardiovascular science as around three million people worldwide have a pacemaker^32^ and many more have an implantable cardioverter-defibrillator. Remote monitoring has significantly transformed the standard of care for people with CIEDs^33^. The pandemic has forced reorganisation of healthcare delivery, and has led to significant increase in remote monitoring of CIEDs^34^.

The diversity and extensive communication between the central groups of scholars in this space highlight the collective efforts towards understanding how digital technologies can impact cardiovascular care. Many opportunities exist in the digital technology space for health system reform, and this requires a united and interdisciplinary effort^35^. Collaborative research networks, such as the American Heart Association’s new Strategically Focused Research Network on Health Technologies and Innovation are moving towards^36^, SOLVE-CHD^37^ and AF-Screen^38^, are other examples of advocacy groups who are promoting discussion and unification of research towards digital technology innovation and capacity building in the CVD care area.

Emerging research areas, including telemedicine and remote monitoring have seen widespread adoption over the past few decades, with the Covid-19 pandemic forcing current healthcare delivery to adapt and change more rapidly than ever before. It is expected that telemonitoring will now shift to include mainstream management of cardiovascular disease care in the coming years. Studies have shown that the *digital divide* is persistent^39–41^, meaning that chasm between those who have access to technologies and the digital literacy to work them, and those who do not, remains a challenge for cardiovascular researchers and practitioners who care for people with CVD. From this review, we saw that research central to digital health in cardiovascular care was conducted in the context of high-income countries only, similar to telemedicine^42^ and digital literacy research^43^. We also observed that there is not a large amount of research being conducted in some minority populations (e.g culturally diverse, disability, low-income). It is noteworthy, however, that there are robust and frequent studies including children, the elderly and rural populations.

It is important to note that digital health innovations in cardiovascular care are not without their challenges, including issues with data ownership, interoperability, and data excess^44,45^. Also, evidence demonstrates that digital technologies are not at the stage of being standalone treatment options and should be integrated into an overall care model. However, the pandemic has enabled the rapid and wide implementation of telehealth and forced us to address any existing gaps and create long-term strategies to close them^27^.

Due to the size of the body of literature included in this analysis, the gaps that have been identified are of a large-scale. Minor gaps in each research stream are not discernible. Additionally, it was not possible to seek causality of why individual papers have become influential or why individual streams have become hot topics. Thus, a scientometric review of a research sub-division or a systematic review should be conducted to identify these. Future research opportunities also exist for a sub-analysis of the metrics used in digital health studies, including patient outcomes (e.g. complication), resource utilization (e.g. rehospitalizations), patient or clinician satisfaction (e.g. QoL questionnaires) or metrics in access or equity. This is an important area to consider, however, outside of the scope of this broader scale analysis. Another limitation is the possibility of a delay in the emergence of a research stream. For example, the OHCA cluster shows distinct characteristics of an emerging research area. While the activities of this cluster were detectable since 2016, this does not indicate that the stream was only introduced to the literature at this time. It may simply mean that it takes some time for literature on this topic to accumulate and become a prominent research stream. With time, we may also see digital technology evolution related to COVID-19 and the impact it has had in CVD care. A repeat analysis in the coming years will likely reveal what the popular research topics and practices were during the pandemic years. Lastly, the definition of digital health technologies is not uniform, and it was initially unclear whether CIEDs fall under this broad category; hence it was not included in the search strings. However, a significant amount of literature regarding CIED research was found (related to keywords such as “remote monitoring” and “digital healthcare”). Therefore, this work was included for completeness.

The advancement of digital health technologies in cardiovascular care, is for some, a solution to many of the medical and public health challenges that are faced every day. Novel technologies are increasingly becoming available and more patients taking part in remote healthcare monitoring, hence, further evaluation and research into digital health technologies, including their long-term effectiveness, is needed. Furthermore, emerging technologies, which are evaluated and/or validated should be considered for implementation into clinical practice as treatment and prevention modalities for CVD. The current study provides a bridge between different segments of digital technology and CVD research, its major research streams and trends within those streams, and provides broader insight that may otherwise not be obtainable from smaller-scale counterpart studies. Outcomes inform future research directions and facilitate collaborations across various sectors of this field to further facilitate the advancement of CVD knowledge.

## Methods

Scientometric methods are increasingly being used to quantitatively measure research metrics and trends within fields and topics across disciplines (e.g. medicine, engineering, science). Traditionally, systematic reviews are used in medicine and healthcare to determine efficacy, effectiveness and other outcomes due to their rigorous and specific nature. However, scientometrics can complement systematic reviews by summarising the *overall trends* of an entire field, sub-field or targeted topic, with virtually no limit on the size of the scholarly literature.

### Search strategy

To retrieve the data for this study, the Web of Science (WoS) Core Collection was accessed and searched in January 2022. A search query was formulated that included terms relevant to *digital health technologies* and *cardiovascular medicine* (see Supplementary Methods). The terms were searched in the titles (Field tag: TI), abstracts (AB) and author keywords (AK) of articles indexed across the WoS Core Collection. Terms were separated from each other using the Boolean operator (OR) and in the case of multiple words within a term, quotation marks were placed around the term. Asterisks were used to allow for term variation (for example, plurals).

Given that the description of the research methodology is included in the abstracts of these articles, we excluded “electronic medical records” (and its variations) from the abstract search string to avoid false positives. However, the term was included in title and author keyword search strings to ensure the inclusion of electronic medical record research. No restrictions were set on the document type or other subcategory. The maximum year was set to December 31, 2021, with no restriction on the minimum. Full bibliographic details of the documents were exported from WoS as text files. Details include the document title, authors, author affiliations, year of publication, source (journal) title, citation count, document type, abstract, author keywords, keywords plus, funding source, full list of document references and conference information, if relevant.

### Semantic analyses

Keyword analysis were conducted using VOSviewer 1.6.15. Keywords provide insight into the temporal shifts in research and scholarly focus. A citation *burst* indicates a sudden increase in the number of citations an article with mentioned keywords receives^46^, the strength or duration, and the start and end year of the peak activity. Bursts of citation with a minimum of four years in duration, aggregated at the level of keywords, were calculated and sorted based on *start year*. For this analysis, only bursts that occurred after 1990 were included, as in the years prior, publications were scattered and sparse, making it difficult to calculate a burst. and abstract analysis were also conducted using VOSviewer 1.6.15. Clusters of terms are formed by the frequency they occur in the title and abstracts of the articles to provides an objective overview about the structure and divisions within this research topic. The frequency of occurrence of the term was set to a minimum of 15.

Analysis of author networks were conducted using VOSviewer 1.6.15. Each author is represented by a node. The link strength, as shown by the thickness of the links between author nodes, represents the number of co-authored documents.

### Temporal analyses

CiteSpace 5.7.R1^46^ was used to complete the document co-citation and citation burst analyses. Using the concept of document co-citation, a methodology developed by Chen^47^, we can obtain a different perspective of the most influential studies within the topic. When two articles appear in the reference list of a third article, they are co-cited by that third article. Essentially, two articles that are frequently co-cited are likely related or are similar in subject. Document co-citation analysis results in a new set of documents, which include valuable knowledge sources for digital technology applications in cardiovascular medicine that are instrumental in the development of this literature but were not captured by the WoS search query.

From document co-citation we can find (i) references with the most local citations (citations from within the literature exclusively relevant to this topic), (ii) references with the strongest citation burst and, 3) references with the highest centrality (document co-citation across multiple clusters). The second of which indicates a heightened attention to an individual article within the field, representing a temporal component of the research topic.

CiteSpace 5.7.R1^46^ is used to generate the dynamic visualisation, which shows insight into the emergence of each research stream since 1990. In the visualisation, parts of the network that have been most active during each year appear more striking, representing co-citation instances during that year. Influential references are identified using the three metrics (local citations, bursts, centrality) However, these metrics are measuring articles that may or may not be about digital health applications, so we must also look at the citing articles with the highest *coverage* to determine which digital health-related articles are citing the most references within the specified research stream.

Digital technologies have increasingly been adopted in the past few decades, hence, for heightened relevance, the time period for the analysis was set for 1990-2021 (at one-year intervals) and the number of look-back years was set to 50, meaning that articles in the reference lists had to be published less than 50 years ago to be included in the analysis. Each individual reference is represented by a node. The relative size of the node is proportional to the number of local citations identified to that reference, and the nodes are connected by links to create a network of major research streams, all contained within the topic of digital health applications in cardiovascular medicine. Links indicate the occurrence of co-citation. Each stream has been provided with a descriptor based on the contents of the cluster. Furthermore, CiteSpace analysis also provides a timeline view of the evolution of research streams. The references of each stream are visualised and aligned across the timeline based on the year of publication from 1950 to 2021.

## Supplementary information

Supplementary Information

## Data Availability

All data is available upon reasonable request to the corresponding author.
