# Supplementary material for: The evolution of digital health technologies in cardiovascular disease research
Source: NPJ Digit Med. 2023 Jan 3;6:1. doi: 10.1038/s41746-022-00734-2 (PMC9808768; doi:10.1038/s41746-022-00734-2)
Supplement: Supplementary file 1 — Supplementary Information [file 41746_2022_734_MOESM1_ESM.pdf]

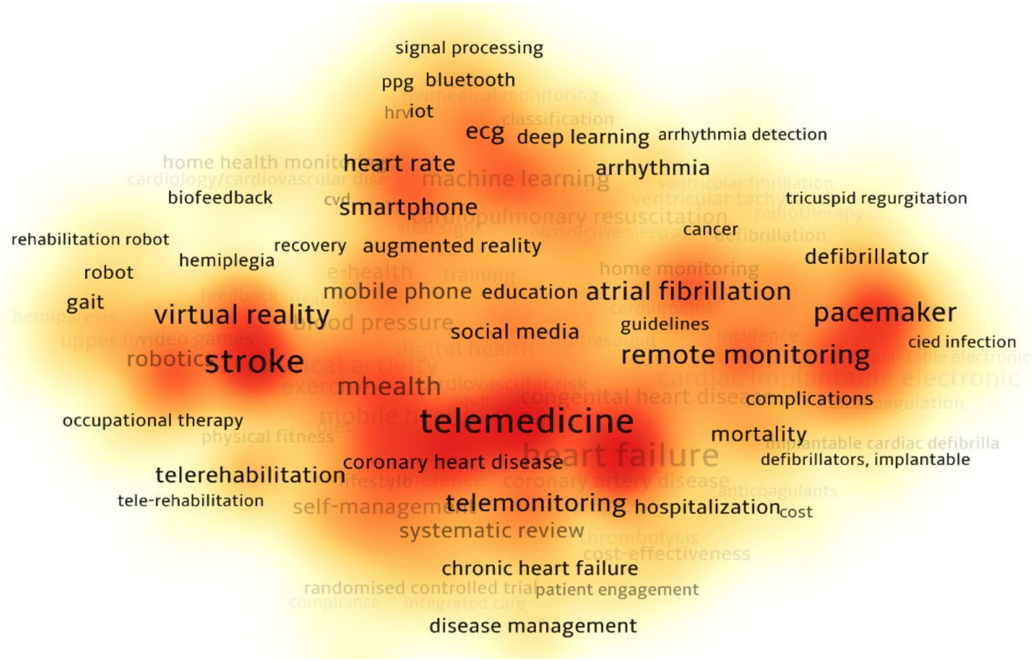

Supplementary Figure 1 Density of co-occurrence of keywords in the literature regarding digital health in cardiovascular medicine. The more significant terms (orange cluster density) are those that occur at increased frequency. An interactive map is available via this link:

https://app.vosviewer.com/?json=https://drive.google.com/uc?id=1dNfiWwb6qAvKTiKEbNXZ0vRcMdCBoYP_

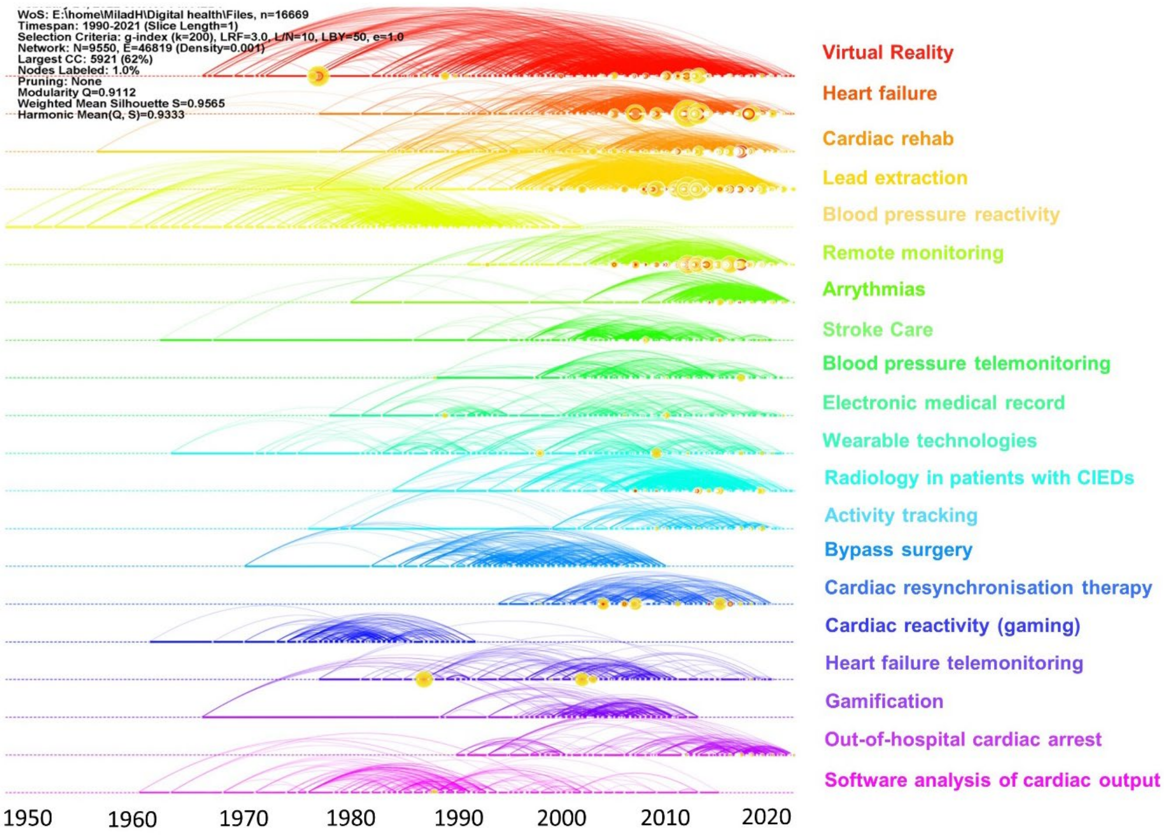

Supplementary Figure 2 Time-line view of the network of document co-citation.

	Cluster descriptor	Topic division	1990	1991	1992	1993	1994	1995	1996	1997	1998	1999	2000	2001	2002	2003	2004	2005	2006	2007	2008	2009	2010	2011	2012	2013	2014	2015	2016	2017	2018	2019	2020	2021
1	Virtual Reality	Technology use in neurological rehabilitation													•			•	•	•	•	•	•	••	••	••	••	••	••	••	••	••	••	••
2	Heart failure	CIEDS & electrophysiology																	•	•	•	•	•	•	••	••	••	••	••	••	••	••	••	••
3	Cardiac rehab	mHealth, secondary prevention & clinical trials																								•	•	••	••	••	••	••	••	••
4	Lead extraction	CIEDS & electrophysiology																					•	•	••	••	••	••	••	••	••	••	••	••
5	Blood pressure reactivity	mHealth, secondary prevention & clinical trials					•																											
6	Remote monitoring	CIEDS & electrophysiology																			•	•	•	••	••	••	••	••	••	••	••	••	••	••
7	Arrythmias	CIEDS & electrophysiology																							•			••	••	••	••	••	••	••
8	Stroke Care	Technology use in neurological rehabilitation										•					•	•	•	•	•	••	•	•	•	•	••	•	••	••		•		
9	Blood pressure telemonitoring	mHealth, secondary prevention & clinical trials																	•	•	•		•	•	•	•	••	••	••	••	••	••	••	••
10	Electronic medical record	Wearable technology applications																		•			•		•	••	•	••	••	••	••	••	••	••
11	Wearable technologies	Wearable technology applications														•					•	•	•			•		••	•	••	••	••	••	••
12	Radiology in patients with CIEDs	CIEDS & electrophysiology																										••	•	••	••	••	•	••
13	Activity tracking	mHealth, secondary prevention & clinical trials																								••	•	•	••	•	••	••	••	••
14	Bypass surgery	CIEDS & electrophysiology														•			•	•	•	•						•						
15	Cardiac resynchronisation therapy	CIEDS & electrophysiology																			•	•	•	•	•	••	•	••	••	••	••	••	•	••
16	Cardiac reactivity (gaming)	mHealth, secondary prevention & clinical trials			•																													
17	Heart failure telemonitoring	CIEDS & electrophysiology																					•	•			••	•	•	•	••	••	••	••
18	Gamification	Technology use in neurological rehabilitation																•		•			•	•										
19	Out-of-hospital cardiac arrest	Emergency cardiovascular care																													•	••	••	••
20	Software analysis of cardiac output	Wearable technology applications												•							•		•		•				•					

Supplementary Figure 3 Analysis of the activity of digital health innovation within cardiovascular research (1990-2021) as reflected in referencing patterns of citing articles. Moderate activity is represented by the symbol • and strong activity is represented by ••. The colour-coding reflects the divisions of the topic as shown in Figure 4. CVD: cardiovascular disease, CIEDs: cardiac implantable electronic devices.

Supplementary Table 1 Clusters of author collaboration[1].

Cluster	Author name (number of documents, number of links, total link strength*)	Research Focus
#1 (n=30)	Auricchio, Angelo (24, 22, 14);); Birgersdotter-Green, Ulrika (18, 4, 4); Blomstrom-Lundqvist, Carina (19, 21,17); Bongiorni, Maria Grazia (27, 23, 17); Brachmann, Johannes (10, 7, 3); Brugada, Josep (10, 17, 9); Burri, Haran (15, 18, 11); Costa, Roberto (10, 9, 4); Cowie, Martin (24, 9, 8); Cronin, Edmond (10, 11, 9); Dagres, Nikolaos (20, 24, 19); Dickstein, Kenneth (14, 15, 12): Epstein, Laurence (12, 18, 7); Glikson, Michael (10, 15, 4); Heidbuchel, Hein (13, 20, 10); Hindricks, Gerhard (31, 23, 23); Kautzner, Josef (15, 22, 12); Kirchhof, Paulus (11, 16, 7); Lampert, Rachel (11, 9, 6); Lewalter, Thorsten (13, 21, 11); Nielsen, Jens Corsedis (18, 19, 11); Passman, Rod (10, 11, 6); Richter, Sergio (10, 2, 9); Russo, Andrea (10, 11, 8); Sanders, Prashanthan (13, 6, 3); Slotwiner, David (10, 20, 7); Taborsky, Milos (15, 14, 10) Vardas, Panos (20, 23, 17); Varma, Niraj (37, 38, 31); Witte, Klaus (10, 1, 4)	-Arrhythmias<br>-Cardiac electrophysiology<br>-Cardiac pacing
#2 (n=22)	Asirvatham, Samuel (32, 12, 21); Boehmer, John (13, 6, 5); Bordachar, Pierre (16, 5, 14); Cha, Yong-Mei (32, 17, 32); Deharo, Jean-Claude (21, 19, 12); Desimone, Christopher (12, 12, 12); Felmlee, Joel (10, 5, 10); Friedman, Paul (63, 18, 56); Haissaguerre, Michel (11, 3, 10); Hayes, David (27, 27, 25); Hodge, David (11, 14, 10); Khairy, Paul (10, 2, 2); Kramer, Daniel (12, 5, 3); Madhavan, Malini (13, 13, 12); Marijon, Eloi (10, 5, 2); Mcleod, Christopher (11, 9, 11); Mueller, Paul (11, 5, 8); Mulpuru, Siva (12, 8, 10); Ploux, Sylvain (13, 3, 12); Saxon, Leslie (15, 8, 6); Singh, Jagmeet (10, 3, 2); Swetz, Keith (10, 2, 4)	-Atrial fibrillation<br>-Imaging<br>-ECG<br>-CIED lead management & extraction
#3 (n=22)	Biffi, Mauro (31, 31, 30); Boriani, Giuseppe (68, 44, 58); Calo, Leonardo (13, 12, 10); Capucci, Alessandro (14, 17, 12); Curnis, Antonio (23, 23, 21); D'onofrio, Antonio (26, 19, 24); Diemberger, Igor (15, 14, 15); Gargaro, Alessio (15, 12, 15); Giacopelli, Daniele (14, 9, 12); Iacopini, Saverio (10, 16, 8); Landolina, Maurizio (17, 17, 16); Lunati, Maurizio (10, 13, 8); Morichelli, Loredana (12, 13, 12); Padeletti, Luigi (11, 23, 11); Palmisano, Pietro (10, 13, 10); Ricci, Renato Pietro (37, 29, 35); Sanitni, Massimo (10, 11, 8); Valsecchi, Sergio (22, 17, 16); Vitolo, Marco (11, 9, 11); Zanotto, Gabriele (21, 17, 18); Ziacchi, Matteo (14, 16, 14)	-Atrial fibrillation<br>-Cardiac resynchronization therapy
#4 (n=22)	Abella, Benjamin (13, 5, 11); Albert, Nancy (10, 8, 8); Asch, David (14, 5, 8); Audebert, Heinrich (27, 5, 16); Blewer, Audrey (13, 6, 13); Bosworth, Hayden (16, 2, 2); Bowles, Kathryn (12, 1, 2); Chumbler, Neale (11, 2, 2); Demaerschalk, Bart (15, 2, 1); Fonarow, Gregg (30, 14, 21); Heidenreich, Paul (12, 12, 10); Hernandez, Adrian (12, 7, 7); Leary, Marion (17, 7, 15); Liu, Jun (10, 2, 3); Merchant, Raina (13, 7, 11); Peterson, Eric (17, 14, 11); Riegel, Barbara (13, 8, 5); Rumsfeld, John (14, 12, 8); Schenkel, Johannes (16, 2, 13); Schwamm, Lee (13, 10, 7); Spindler, Helle (10, 1, 1); Wang, Wei (11, 2, 1)	-Cardiopulmonary resuscitation<br>-Emergency cardiovascular care<br>-Telemedicine within stroke care
#5 (n=18)	Brunetti, Natale Daniele (16, 4, 11); Capomolla, Soccorso (10, 5, 8); Di Biase, Matteo (10, 1, 8); Ellis, Christopher (11, 1, 1); Fedele, Francesco (11, 8, 7); Giordano, A (15, 6, 15); Glisenti, F (15, 5, 12); Hobbs, Richard (10, 2, 5); Johnson, Paul (12, 2, 6); Kario, Kazuomi (11, 3, 3); Lip, Gregory (31, 23, 16); McManus, Richard (10, 3, 6); Natale, Andrea (11, 4, 4); Omboni, Stefano (16, 4, 7); Parati, Gianfranco (27, 15, 13); Scalvini, Simonetta (31, 9, 25); Zanelli, Emanuella (16, 5, 16)	-Telecardiology<br>-Thromboembolic disease management<br>-Chronic HF<br>-Blood pressure monitoring
#6 (n=15)	Essebag, Vidal (12, 5, 11); Healey, Jeff (16, 17, 13); Hussein, Ayman (11, 6, 10); Kennergren, Charles (15, 21, 12); Korantzopoulos, Panagiotis (10, 9, 5); Krahn, Andrew (13); Mittal, Suneet (26, 29, 18); Philippon, Francois (19, 14, 12); Poole, Jeanne (11, 23, 8); Rickard, John (11, 12, 9); Schaller, Robert (10, 6, 1); Tarakji, Khandoun (37, 27, 34); Verma, Atul (13, 11, 12); Wazni, Oussama (20, 12, 18); Wilkoff, Bruce (57, 31, 49)	-Cardiac resynchronization therapy<br>-Transvenous Lead Extractions<br>-Atrial fibrillation
#7 (n=15)	Gasior, Mariusz (13, 4, 13); Grabowski, Marcin (19, 10, 17); Jachec, Wojciech (13, 8, 13); Kalarus, Zbigniew (16, 17, 16); Kutarski, Andrzej (17, 10, 17); Lenarczyk, Radoslaw (10, 8, 10); Opolski, Grzegorz (22, 11, 22); Piotrowicz, Ewa (39, 22, 35); Piotrowicz, Ryszard (36, 20, 35); Polewczyk, Anna (15, 5, 13); Potpara, Tanjana (10, 13, 10); Steckiewicz, R (13, 2, 3); Szalewska, Dominika (10, 5, 6); Tajstra, Mateusz (12, 3, 11); Zareba, Wojciech (12, 12, 10)	-Telemonitoring in HF<br>-Stroke prevention in atrial fibrillation<br>-Cardiac resynchronization therapy
#8 (n=14)	Ashley, Euan (11, 3, 5); Clark, Robyn (26, 3, 14); Cleland, John (27, 6, 15); Clifford, Gari (12, 3, 3); Harrington, Robert (10, 4, 9); Inglis, Sally (17, 3, 14); Martin, Seth (19, 2, 1); Peiris, David (14, 6, 11); Perel, Pablo (16, 9, 9); Prabhakaran, Dorairaj (10, 7, 9); Prieto-Merino, David (10, 6, 8); Tandon, Nikhil (10, 4, 8); Tarassenko, Lionel (11, 5, 5); Turakhia, Mintu (26, 19, 18)	-mHealth and telemonitoring in HF<br>-ECG data analysis
#9 (n=11)	Chow, Clara (44, 14, 41); Islam, Shariful (10, 7, 9); Jiang, Yannan (20, 3, 17) Lear, Scott (15, 5, 1); Maddison, Ralph (31, 8, 25); Redfern, Julie (49, 13, 45); Santo, Karla (12, 7, 12); Stewart, Ralph (11, 3, 11); Thiagalingam, Aravinda (25, 7, 24); Wang, Jing (13, 1, 1); Robyn Whittaker (21, 9, 19)	-Digital health innovations for secondary prevention of CHD
#10 (n=11)	Baddour, Larry (50, 18, 44); Carrillo, Roger (24, 13, 12); Desimone, Daniel (16, 9, 15); Greenspon, Arnold (17, 16, 13); Henrikson, Charles (10, 5, 6); Miro, Jose (15, 6, 9); Prutkin, Jordan (10, 6, 9); Sohail, Muhammed Rizwan (56, 23, 48); Steckelberg, James (18, 10, 18); Uslan, Daniel (15, 11, 15); Wilson, Walter (23, 11, 23)	-Infections associated with CIEDs

*Total link strength represents the instances of co-authorships within the network.

[1] Smaller clusters that are not included in the table (n ≤ 10), can be visualised in the interactive map using the link under Figure 9.

Supplementary Table 2 Most cited papers (WoS dataset)

Title	Authors (year)	Journal	Citation count (global)
Flexible polymer transistors with high pressure sensitivity for application in electronic skin and health monitoring	Schwartz, Tee (1)	Nature Communications	1364
Reducing children's television viewing to prevent obesity - A randomized controlled trial	Robinson (2)	JAMA	1266
Kubios HRV - Heart rate variability analysis software	Tarvainen, Niskanen (3)	Computer Methods and Programs in Biomedicine	1159
Youth Risk Surveillance- United States, 2013	Kann, Kinchen (4)	MMWR Surveillance Summaries	1077
Two-dimensional strain - A novel software for real-time quantitative echocardiographic assessment of myocardial function	Leitman, Lysyansky (5)	Journal of the American Society of Echocardiography	985
Medication Adherence: WHO Cares?	Brown and Bussell (6)	Mayo Clinic Proceedings	924
Effects of robot-assisted therapy on upper limb recovery after stroke: a systematic review	Kwakkel, Kollen (7)	Neurorehabilitation and Neural Repair	932
Robot-Assisted Therapy for Long-Term Upper-Limb Impairment after Stroke	Lo, Guarino (8)	New England Journal of Medicine	832
Robot-assisted movement training compared with conventional therapy techniques for the rehabilitation of upper-limb motor function after stroke	Lum, Burgar (9)	Archives of Physical Medicine and Rehabilitation	770
Telemonitoring in Patients with Heart Failure	Chaudhry, Mattera (10)	New England Journal of Medicine	742

Supplementary Table 3 Most cited papers by local citation count (document co-citation output)

Title	Authors (year)	Journal	Citation count (local)
Telemonitoring in Patients with Heart Failure	Chaudhry, Mattera (10)	New England Journal of Medicine	332
2016 ESC Guidelines for the diagnosis and treatment of acute and chronic heart failure: The Task Force for the diagnosis and treatment of acute and chronic heart failure of the European Society of Cardiology (ESC)Developed with the special contribution of the Heart Failure Association (HFA) of the ESC	Ponikowski, Voors (11)	European Heart Journal	291
Update on Cardiovascular Implantable Electronic Device Infections and Their Management: A Scientific Statement From the American Heart Association	Baddour, Epstein (12)	Circulation	270
The post-stroke hemiplegic patient. 1. a method for evaluation of physical performance	Fugl-Meyer, Jääskö (13)	Scandinavian Journal of Rehabilitation Medicine	245
Impact of Remote Telemedical Management on Mortality and Hospitalizations in Ambulatory Patients With Chronic Heart Failure The Telemedical Interventional Monitoring in Heart Failure Study	Koehler, Winkler (14)	Circulation	232
16-year trends in the infection burden for pacemakers and implantable cardioverter-defibrillators in the United States: 1993 to 2008	Greenspon, Patel (15)	Journal of the American College of Cardiology	229
Wireless pulmonary artery haemodynamic monitoring in chronic heart failure: a randomised controlled trial	Abraham, Adamson (16)	Lancet	201
Noninvasive home telemonitoring for patients with heart failure at high risk of recurrent admission and death: the Trans-European Network-Home-Care Management System (TEN-HMS) study	Cleland, Louis (17)	Journal of the American College of Cardiology	198
Implant-based multiparameter telemonitoring of patients with heart failure (IN-TIME): a randomised controlled trial	Hindricks, Taborsky (18)	Lancet	196
Transvenous lead extraction: Heart Rhythm Society expert consensus on facilities, training, indications, and patient management: this document was endorsed by the American Heart Association (AHA)	Wilkoff, Love (19)	Heart Rhythm	190

Supplementary Table 4 Articles with strongest bursts of citation in the literature (document co-citation output)

Title	Author(s) (year)	Journal	Begin	End	Strength
Noninvasive home telemonitoring for patients with heart failure at high risk of recurrent admission and death: the Trans-European Network-Home-Care Management System (TEN-HMS) study	Cleland, Louis (17)	Journal of American College of Cardiology	2006	2014	36.55
Structured telephone support or telemonitoring programmes for patients with chronic heart failure	Inglis, Clark (20)	Cochrane Database of Systematic Reviews	2011	2015	31.81
Randomized trial of a daily electronic home monitoring system in patients with advanced heart failure: the Weight Monitoring in Heart Failure (WHARF) trial	Goldberg, Piette (21)	American Heart Journal 2003	2005	2012	30.79
Telemonitoring or structured telephone support programmes for patients with chronic heart failure: systematic review and meta-analysis	Clark, Inglis (22)	BMJ	2008	2013	26.47
A Meta-Analysis of Remote Monitoring of Heart Failure Patients	Klersy, De Silvestri (23)	Journal of American College of Cardiology	2010	2016	24.48

Supplementary Table 5 Major research streams (clusters) as determined by co-citation of references in the literature of digital health applications in cardiovascular medicine. eMR: electronic medical record; BP: Blood pressure; HRV: heart rate variability

Author descriptor<br><br>Other descriptors extracted from titles of citing articles	-size<br>-silhouette score<br>-mean year (reference)<br>-year range (reference)<br>-mean year (citing)<br>-year range (citing)	Influential references			Citing articles with highest coverage
		Local citation count	Citation burst strength (strength, duration of burst)	Centrality	
Virtual Reality<br><br>-chronic stroke<br>-stroke rehabilitation<br>-robot-assisted therapy	-S=795<br>-SS=0.983<br>-MY (ref)=2005<br>-YR (ref)=1964-2020<br>-MY (citing)=2015<br>-YR (citing)=1997-2021	1. Fugl-Meyer, Jääskö (13) (245)<br>2. Laver, George (24) (162)<br>3. Lo, Guarino (8) (123)<br>4. Kwakkel, Kollen (7) (121)<br>5. Folstein, Folstein (25) (99)<br>6. Langhorne, Coupar (26) (97)<br>7. Bohannon, Larkin (27) (97)<br>8. Langhorne, Bernhardt (28) (94)<br>9. Saposnik, Teasell (29) (87)<br>10. Lum, Burgar (30) (85)	1. Hesse, Werner (31) (20.13, 2007-2014)<br>2. Volpe, Krebs (32) (19.5, 2001-2014)<br>3. Kwakkel, Kollen (7) (19.4, 2009-2015)<br>4. Krebs, Hogan (33) (19.27, 2000-2013)<br>5. Aisen, Krebs (34) (18.32, 1999-2013)<br>6. Lum, Burgar (30) (18.1, 2005-2014)<br>7. Burgar, Lum (35) (17.72, 2001-2012)<br>8. Prange, Jannink (36) (17.34, 2008-2014)<br>9. Lo, Guarino (8) (17.26, 2011, 2016)<br>10. Fasoli, Krebs (37) (16.38, 2006-2012)	1. Piron, Turolla (38) (0.03)<br>2. Housman, Scott (39) (0.02)<br>3. Volpe, Krebs (40) (0.02)	1. Laver, Lange (41) (54)<br>2. Basteris, Nijenhuis (42) (50)<br>3. Laver, George (43) (44)<br>4. Kwakkel, Kollen (7) (36)<br>5. Masiero, Celia (44) (34)<br>6. Duret and Gracies (45) (34)<br>7. Brokaw, Murray (46) (31)<br>8. Sivan, O'Connor (47) (31)<br>9. Acosta, Dewald (48) (30)<br>10. Mehrholz, Hadrich (49) (30)
Heart failure<br><br>-home telemonitoring<br>-heart failure patients<br>-virtual reality	-S=552<br>-SS=0.881<br>-MY (ref)=2007<br>-YR (ref)=1975-2020<br>-MY (citing)=2016<br>-YR (citing)=2005-2021	1. Chaudhry, Mattera (10) (332)<br>2. Ponikowski, Voors (11) (291)<br>3. Koehler, Winkler (14) (232)<br>4. Cleland, Louis (17) (198)<br>5. Inglis, Clark (20) (151)<br>6. Ong, Romano (50) (141)<br>7. Clark, Inglis (22) (140)<br>8. Klersy, De Silvestri (23) (123)<br>9. Goldberg, Piette (21) (110)<br>10. Inglis, Clark (51) (110)	1. Cleland, Louis (17) (36.55, 2006-2014)<br>2. Inglis, Clark (20) (31.81, 2011-2015)<br>3. Goldberg, Piette (21) (30.79, (2005-2012)<br>4. Clark, Inglis (22) (26.47, 2008-2013)<br>5. Klersy, De Silvestri (23) (24.48, 2010, 2016)<br>6. Louis, Turner (52) (23.4, 2005-2011)<br>7. McMurray, Adamopoulos (53) (21.93, 2014-2017)<br>8. Roger, Go (54) (18.53, 2012-2015)<br>9. Jerant, Azari (55) (18.27, 2003-2010)<br>10. Benatar, Bondmass (56) (18, 2006-2014)	1. Jerant, Azari (55) (0.02)<br>2. Artinian, Harden (57) (0.02)<br>3. Higgins, Altman (58) (0.02)<br>4. Hsieh and Shannon (59) (0.02)<br>5. Finkelstein, Speedie (60) (0.02)	1. Flodgren, Rachas (61) (96)<br>2. Inglis, Clark (62) (89)<br>3. Inglis, Clark (20) (85)<br>4. Di Lenarda, Casolo (63)<br>5. Aronow and Shamliyan (64) (39)<br>6. Lin, Yuan (65) (36)<br>7.Dickinson, Allen (66) (35)<br>8. Banchs and Scher (67) (34)<br>9. Givertz, Stevenson (68) (33)<br>10. Bertini, Marcantoni (69) (33)
Cardiac rehabilitation<br><br>-coronary heart disease<br>-secondary prevention<br>-cardiovascular disease<br>-medication adherence	-S=487<br>-SS=0.896<br>-MY (ref)=2009<br>-YR (ref)=1954-2020<br>-MY (citing)=2018<br>-YR (citing)=1995-2021	1. Chow, Redfern (70) (131)<br>2. Heran, Chen (71) (128)<br>3. Varnfield, Karunanithi (72) (107)<br>4.Burke, Ma (73) (101)<br>5. Craig, Marshall (74) (76)<br>6. Kroenke, Spitzer (75) (69)<br>7. Piepoli, Hoes (76) (69)<br>8. Eysenbach and Consort (77) (66)<br>9. Maddison, Pfaeffli (78) (66)<br>10. Free, Phillips (79) (66)	1. Chow, Redfern (70) (13.05, 2017-2021)<br>2. McMurray, Adamopoulos (53) 12.22, 2013-2016)<br>3. Burke, Ma (73) (9.94, 2016-2021)<br>4. Zutz, Ignaszewski (80) (9.24, 2012-2016)<br>5. Chan, Tetzlaff (81) (8.99, 2017-2021)<br>6. Neubeck, Lowres (82) (7.9, 2013-2018)<br>7. Fjeldsoe, Marshall (83) (7.17 2014-2018)<br>8. Thakkar, Kurup (84) (7.04, 2016-2021)<br>9. Lester, Ritvo (85) (7.03, 2015-2018)<br>10. Dale, Whittaker (86) (7, 2017-2021)		1. Flodgren, Rachas (61) (52)<br>2. Burke, Ma (73) (38)<br>3. Dale, Whittaker (86) (29)<br>4. Devi, Singh (87) (26)<br>5. Adler, Martin (88) (25)<br>6. Jin, Khonsari (89) (24)<br>7. Clark, Conway (90) (23)<br>8. (91) (22)<br>9. Brouwers, Kraal (92) (21)<br>10. Frederix, Vanhees (93) (21)
Lead extraction<br><br>-risk factor<br>-transvenous lead extraction	-S=424<br>-SS=0.954<br>-MY (ref)=2009<br>-YR (ref)=1972-2020	1.Baddour, Epstein (12) (270)<br>2.Greenspon, Patel (15) (229)<br>3.Wilkoff, Love (19) (190)<br>4.Sohail, Uslan (94) (164)	1. Sohail, Uslan (94) (11.77, 2010-2014)<br>2. BLOOM, HEEKE (103) (11.72, 2010-2015)	1. Boriani, Maniadakis (109) (0.04)<br>2. DerSimonian and Laird (110) (0.02)	1. Kusumoto, Schoenfeld (95) (91)<br>2. Blomström-Lundqvist, Traykov (111) (89)<br>3. Kusumoto, Schoenfeld (95) (71)

-infective endocarditis<br>-heart failure<br>-antibacterial envelope	-MY (citing)=2017<br>-YR (citing)=2010-2021	5. Kusumoto, Schoenfeld (95) (160)<br>6. Klug, Balde (96) (160)<br>7. Mond and Proclemer (97) (148)<br>8. Voigt, Shalaby (98) (134)<br>9 .Sohail, Henrikson (99) (117)<br>10. Voigt, Shalaby (100) (116)<br>11.de Oliveira, Martinelli (101) (112)<br>12. Poole, Gleva (102) (105)	3. Baddour, Epstein (12) (11.4 2012-2016)<br>4. Sohail, Uslan (104) (10.39, 2010-2014)<br>5. Habib, Lancellotti (105) (10.26, 2017-2021)<br>6. Chamis, Peterson (106) (10.07, 2010-2015)<br>7. Poole, Gleva (102) (9.32, 2014-2017)<br>8. .Sohail, Uslan (107) (9.17, 2010-2016)<br>9. Greenspon, Patel (15) (9.06, 2013-2018)<br>10. Al-Khatib, Lucas (108) (9.03, 2010-2015)		4. Padfield, Steinberg (112) (55)<br>5. Palmeri, Kramer (113) (54)<br>6. Frausing, Kronborg (114) (49)<br>7. Polyzos, Konstantelias (115) (48)<br>8. Palraj, Farid (116) (47)<br>9. Arnold and Chu (117) (46)<br>10. Han, Hawkins (118) (45)
Blood pressure reactivity<br><br>-type-a-behaviour<br>-challenging task<br>-contingent reinforcement<br>-physiological reactivity	-S=417<br>-SS=0.985<br>-MY (ref)=1984<br>-YR (ref)= 1945-2000<br>-MY (citing)=1993<br>-MY (citing)=1991-1997	1. Falkner, Kushner (119) (10)<br>2. Borghi, Boschi (120) (7)<br>3. Braden, Leatherbury (121) (7)<br>4. Park and Menard (122) (6)	1. Falkner, Kushner (119) (6.77, 1991-1995)<br>2. Borghi, Boschi (120) (4.56, 1991-2001)<br>3. Braden, Leatherbury (121) (4.53, 1993-2003)<br>4. Park and Menard (122) (3.98, 1991-1998)	1. Anderson and Armstead (123) (0.03)<br>2. Anderson, Land (124) (0.03)<br>3. Beaglehole, Salmond (125) (0.02)	1. Lyness (126) (77)<br>2. Murphy, Alpert (127) (64)<br>3. Saab, Tischenkel (128) (48)<br>4. Miller and Sita (129) (39)<br>5. Miller, Friese (130) (37)<br>6. Miller (131) (29)<br>7. Sorof, Forman (132) (26)<br>8. Treiber, Raunikar (133) (26)<br>9. Musante, Raunikar (134) (21)
Remote monitoring<br><br>-atrial high-rate episode<br>-atrial fibrillation<br>-cardiac rehabilitation<br>-embolic stroke	-S=404<br>-SS=0.902<br>-MY (ref)=2010<br>-YR (ref)=1989-2020<br>-MY (citing)=2016<br>-YR (citing)=2007-2021	1. Abraham, Adamson (16) (201)<br>2. Hindricks, Taborsky (18) (196)<br>3. Varma, Epstein (135) (183)<br>4. Crossley et al., 2011) (149)<br>5. Healey, Connolly (136) (136)<br>6. Slotwiner, Varma (137) (123)<br>7. Saxon, Hayes (138) (118)<br>8. Yancy, Jessup (139) (116)<br>9. Landolina, Perego (140) (111)<br>10. Mabo, Victor (141) (104)	1. Raatikainen, Uusimaa (142) (21.37, 2009-2016)<br>2. Varma, Epstein (135) (21.13, 2011-2016)<br>3. Ricci, Morichetli (143) (19.32, 2008-2015)<br>4. Wilkoff, Auricchio (144) (18.98, 2009-2015)<br>5. Lazarus (145) (17.92, 2008-2014)<br>6. Schoenfeld, Compton (146) (17.86, 2007-2016)<br>7. Crossley et al., 2011) (16.39, 2012-2016)<br>8. Wilkoff, Auricchio (147) (16.2, 2010-2013)<br>9. Marzegalli, Lunati (148) (15.96, 2009-2015)<br>10. Fauchier, de Bouët du Portal (149) (15.89, 2007-2014)	1. Wilkoff, Auricchio (144) (0.02)<br>2. Gladstone, Spring (150) (0.02)<br>3. Hindricks, Pokushalov (151) (0.02)<br>4. Adams, Bendixen (152) (0.02)	1. Dubner, Auricchio (153) (56)<br>2. Flodgren, Rachas (154) (43)<br>3. Imberti, Tosetti (155) (41)<br>4. Braunschweig, Anker (156) (37)<br>5. Sanna (157) (32)<br>6. Heywood, Jermyn (158) (31)<br>7. Lucà, Cipolletta (159) (30)<br>8. Freedman, Hindricks (160) (30)<br>9. Noseworthy, Kaufman (161) (29)<br>10. Alvarez, Cronin (162) (29)
Arrythmias<br><br>-atrial fibrillation<br>-detecting atrial fibrillation<br>-heart failure<br>-heart rhythm<br>-diagnostic accuracy	-S=211<br>-SS=0.975<br>-MY (ref)=2015<br>-YR (ref)=1981-2020<br>-MY (citing)=2019<br>-YR (citing)=2006-2021	1. Lau, Lowres (163) (70)<br>2. Lowres, Neubeck (164) (49)<br>3. Tison, Sanchez (165) (45)<br>4. Bumgarner, Lambert (166) (44)<br>5. Barrett, Komatireddy (167) (40)<br>6. Haberman, Jahn (168) (40)<br>7.Chan, Wong (169) (38)<br>8. McManus, Lee (170) (34)<br>9. Turakhia, Desai (171) (33)<br>10. Svennberg, Engdahl (172) (31)	1. Scully, Lee (173) (10.52, 2013-2017)<br>2. Boulos, Wheeler (174) (8.48, 2012-2016)<br>3. McManus, Lee (170) (7.07, 2016-2019)<br>4. Poh, McDuff (175) (5.34, 2015-2018)<br>5. Tarakji, Wazni (176) (4.95, 2016-2019)<br>6. Verkruysse, Svaasand (177) (4.34, 2015-2018)<br>7. Haberman, Jahn (178) (4.32, 2016-2019)	1. Bosworth, Powers (182) (0.04)<br>2. McManus, Chong (183) (0.04)<br>3. Boriani, Laroche (184) (0.04)<br>4. Turakhia, Hoang (185) (0.03)<br>5. Ades, Pashkow (179) (0.02)<br>6. Heidbuchel and Hindricks (186) (0.02)<br>7. Merchant, Abella (187) (0.02)	1. Varma, Cygankiewicz (188) (144)<br>2. Lopez Perales, Van Spall (189)(29)<br>3. Li, White (190)(23)<br>4. Tadi, Mehrang (191) (21)<br>5. Freedman, Hindricks (160) (20)<br>6. König, Bollmann (192)(19)<br>7. Krittanawong, Johnson (193) (18)<br>8. Pereira, Tran (194) (17)<br>9. Folke, Andelius (195) (16)<br>10. McConnell, Turakhia (196) (15)

			8. Ades, Pashkow (179) (4.29, 2003-2015)<br>9. Bruining, Caiani (180) (4.25, 2016-2019)<br>10. Jonathan and Leahy (181) (4.05, 2012-2015)		
Stroke Care<br><br>-integrative stroke care<br>-acute stroke<br>-telemedic pilot project	-S=191<br>-SS=0.978<br>-MY (ref)=2006<br>-YR (ref)=1960-2018<br>-MY (citing)=2012<br>-YR (citing)=1999-2020	1. Braun and Clarke (197) (74)<br>2. Audebert, Schenkel (198) (56)<br>3. Go, Mozaffarian (199) (55)<br>4. Schwamm, Holloway (200)(43)<br>5. Meyer, Raman (201)(43)<br>6. Marler (202) (42)<br>7. Audebert, Kukla (203) (41)<br>8. Shafqat, Kvedar (204) (39)<br>9. Handschu, Littmann (205) (34)<br>10. Levine and Gorman (206) (33)	1. Shafqat, Kvedar (204) (18.89, 2003-2013)<br>2. Audebert, Kukla (203) (15, 2005-2010)<br>3. Wang, Lee (207) (14.38, 2005-2013)<br>4. Wiborg and Widder (208) (13.41, 2005-2010)<br>5. LaMonte, Bahouth (209) (13.1, 2004-2010)<br>6. Meyer, Raman (201) (13.06, 2009-2016)<br>7. Schwamm, Rosenthal (210) (12.65, 2005-2010)<br>8. Audebert, Kukla (211) (12.02, 2007-2010)<br>9. Schwamm, Holloway (212) (11.95, 2010-2014)<br>10. Demaerschalk, Miley (213) (11.95, 2007-2015)		1. Audebert, Berger (214) (39)<br>2. Schwamm, Audebert (215) (37)<br>3. Schwamm, Holloway (212) (35)<br>4. Demaerschalk, Miley (213) (32)<br>5. Demaerschalk (216) (28)<br>6. Levine and Gorman (206) (23)<br>7. Johansson and Wild (217) (18)<br>8. Pervez, Silva (218) (18)<br>9. Wechsler, Demaerschalk (219) (18)<br>10. Ickenstein, Horn (220) (16)
Blood pressure telemonitoring<br><br>-hypertension management<br>-home blood pressure telemonitoring<br>-heart failure	-S=188<br>-SS=0.922<br>-MY (ref)=2008<br>-YR (ref)=1986-2019<br>-MY (citing)=2016<br>-YR (citing)=2005-2021	1. Shamseer, Moher (221) (107)<br>2. Chobanian, Bakris (222) (61)<br>3. Green, Cook (223) (57)<br>4. Morisky, Green (224) (39)<br>5. McManus, Mant (225) (38)<br>6. Margolis, Asche (226) (35)<br>7. Hippisley-Cox, Coupland (227) (34)<br>8. Lewington, Clarke (228) (31)<br>9. Bodenheimer, Wagner (229) (29)<br>10. Wootton (230) (28)	1. Wootton (230) (8.63, 2014-2017)<br>2. Friedman, Kazis (231) (7.21, 2006-2011)<br>3. Rogers, Small (232) (7.21, 2006-2011)<br>4. Chobanian, Bakris (222) (6.66, 2005-2013)<br>5. Shea, Weinstock (233) (6.63, 2010-2016)<br>6. Ekeland, Bowes (234) (6.39, 2014-2017)<br>7. Bodenheimer, Wagner (229) (6.39, 2011-2016)<br>8. Artinian, Washington (235) (6.02, 2005-2010)<br>9. McKinstry, Hanley (236) (6.02, 2015-2018)<br>10. Scherr, Zweiker (237) (5.57, 2007-2010)	1. Chobanian, Bakris (222) (0.03)<br>2.Green, Cook (238) (0.02)<br>3. Pickering, Hall (239) (0.02)	1. Flodgren, Rachas (154) (33)<br>2. Omboni and Ferrari (240) (23)<br>3. Burke, Ma (73) (19)<br>4. Paré, Jaana (241) (18)<br>5. Parati and Omboni (242) (16)<br>6. Omboni, Panzeri (243) (16)<br>7.Mc Kinstry, Hanley (244) (16)<br>8. Ringeval, Wagner (245)(15)<br>9. Oliveira, Paula (246) (15)

Electronic medical record<br><br>-artificial intelligence<br>-resolution recovery software<br>-remote monitoring<br>-using eMR	-S=169<br>-SS=0.969<br>-MY (ref)=2005<br>-YR (ref)=1976-2019<br>-MY (citing)=2017<br>-YR (citing)=1994-2021	1. D'Agostino, Vasan (247) (81)<br>2. Yusuf, Hawken (248) (75)<br>3. Charlson, Pompei (249) (62)<br>4. Lloyd-Jones (250) (52)<br>5. Wilson, D'Agostino (251) (49)<br>6. Conroy, Pyörälä (252) (40)<br>7. Craig, Dieppe (253) (39)<br>8. Grundy (254) (29)<br>9. Hannun, Rajpurkar (255) (29)<br>10. James, Oparil (256) (25)	1. James, Oparil (256) (6.99, 2015-2018)<br>2. Stone, Robinson (257) (6.64, 2015-2018)<br>3. Cleeman (258) (5.73, 2007-2013)<br>4. Wilson, D'Agostino (251) (5.55, 1999-2008)<br>5. Garg, Adhikari (259) (5.09, 2010-2013)<br>6. LeCun, Bengio (260) (4.93, 2017-2021)<br>7. Craig, Dieppe (253) (4.93, 2015-2018)<br>8. Cabana, Rand (261) (3.73, 2011-2017)<br>9. (262) (3.61, 2017-2021)		1. Pashos, Normand (263) (28)<br>2. Krittanawong, Johnson (193) (15)<br>3. Wasimuddin, Elleithy (264) (14)<br>4. Davis, Abidi (265) (12)<br>5.Ting, Peng (266) (10)<br>6. Seetharam, Kagiyama (267) (10)<br>7. (268) (10)
Wearable technologies<br><br>-heart rate variability<br>-heart failure<br>-smartphone seismocardiogram<br>-HRV biofeedback<br>-cardiovascular abnormalities	-S=169<br>-SS=0.982<br>-MY (ref)=2002<br>-YR (ref)=1961-2019<br>-MY (citing)=2017<br>-YR (citing)=1991-2021	1. Allen (269) (95)<br>2. Camm (270) (91)<br>3. Pantelopoulos and Bourbakis (271) (48)<br>4. Inan, Migeotte (272) (42)<br>5. Shaffer and Ginsberg (273) (41)<br>6. Rajendra Acharya, Paul Joseph (274) (41)<br>7. Patel, Park (275) (33)<br>8. (276) (29)<br>9. Tamura, Maeda (277) (28)<br>10. Schäfer and Vagedes (278) (26)	1. (270) (10.35, 2002-2010)<br>2. Hamilton and Tompkins (279) (7.31, 2010-2017)<br>3. Tamura, Maeda (277) (6.41, 2016-2021)<br>4. Lee, Chen (280) (5.75, 2008-2014)<br>5. (281) (4.22, 2008-2011)<br>6. Hung and Zhang (282) (4.22, 2008-2011)<br>7. Patel, Park (275) (3.77, 2014-2021)	1. Akselrod, Gordon (283) (0.08)<br>2. Carroll, Turner (284) (0.07)<br>3. Rajendra Acharya, Paul Joseph (274) (0.06)<br>4.Billman (285) (0.03)	1. Sloan and Bigger (286) (30)<br>2. Chow and Yang (287) (21)<br>3. Rapalis, Petrėnas (288) (15)<br>4. (289) (14)<br>5. Blum, Rockstroh (290) (14)<br>6. Sufi and Khalil (291) (13)<br>7. Sufi, Khalil (292) (13)<br>8. (293) (12)<br>9. Gratze, Fortin (294) (12)<br>10. Vogel, Auinger (295) (11)
Radiology in patients with CIEDs<br><br>-Undergoing radiotherapy<br>-radiologists consensus statement<br>-magnetic resonance<br>-Canadian Association	-S=160<br>-SS=0.988<br>-MY (cited)=2009<br>-YR (ref)=1982-2020<br>-MY (citing)=2018<br>-YR (citing)=2010-2021	1. Indik, Gimbel (296) (75<br>2. Brignole, Auricchio (297) (72)<br>3. Crossley, Poole (298) (71)<br>4. Russo, Costa (299) (63)<br>5. Kalin and Stanton (300) (60)<br>6. Hurkmans, Knegjens (301) (55)<br>7. Nazarian, Hansford (302) (50)<br>8. Wilkoff, Bello (303) (46)<br>9. Beinart and Nazarian (304) (42)<br>10. Epstein, Abraham (305) (40)	1. Nazarian, Hansford (302) (9.96, 2015-2019)<br>2. Wilkoff, Bello (303) (8.81, 2012-2018)<br>3. Levine, Gomes (306) (7.59, 2010-2018)<br>4. Sommer, Naehle (307) (7.38, 2015-2018)<br>5. Gimbel, Bello (308) (7.38, 2015-2018)<br>6. Higgins, Gard (309) (6.99, 2015-2018)<br>7. Kalin and Stanton (300) (6.79, 2014-2018)<br>8. Roguin, Schwitter (310) (6.43, 2010-2015)<br>9. Cohen, Brinton (311) (6.2, 2014-2018)<br>10. Roguin, Zviman (312) (6.07, 2008-2017)		1. Indik, Gimbel (313) (43)<br>2. Yeung, Chacko (314) (32)<br>3. Muthalaly, Nerlekar (315) (32)<br>4. Nyotowidjojo, Skinner (316) (23)<br>5. Zecchin, Severgnini (317) (22)<br>6. Kalb, Indik (318) (22)<br>7. Jung, Jäckle (319) (22)<br>8. Ipek and Nazarian (320) (20)<br>9. Shah, Patel (321) (20)<br>10. Miften, Mihailidis (322) (20)
Activity tracking<br><br>-Validation study<br>-energy expenditure<br>-heart rate<br>-apple watch<br>-physical activity	-S=156<br>-SS=0.972<br>-MY (ref)=2011<br>-YR (ref)=1974-2019<br>-MY (citing)=2018<br>-YR (citing)=2010-2021	1. Widmer, Collins (323) (53)<br>2. Garber, Blissmer (324) (52)<br>3. Moher, Liberati (325) (46)<br>4. Bravata, Smith-Spangler (326) (45)<br>5. Shcherbina, Mattsson (327) (44)<br>6. Egger, Smith (328) (36)<br>7. Haskell, Lee (329) (34)<br>8. Wallen, Gomersall (330) (33)<br>9. Martin, Feldman (331) (30)<br>10. Evenson, Goto (332) (28)	1. Evenson, Goto (332) (8.12, 2017-2021)<br>2. Higgins (333) (7.52, 2012-2015)<br>3. Graves, Ridgers (334) (7.13, 2013-2016)<br>4. Haskell, Lee (329) (6.85, 2014-2018)<br>5. Deutsch, Borbely (335) (5.66, 2011-2015)<br>6. Germano, Hoes (336) (5.16, 2014-2018)<br>7. Lanningham-Foster, Foster (337) (4.86, 2011-2014)<br>8. Ferguson, Rowlands (338) (4.6, 2016-2019)<br>9. Graf, Pratt (339) (4.32, 2011-2014)	1. Wang, Blackburn (340) (0.03<br>2. Egger, Smith (328) (0.02)<br>3. Dooley, Golaszewski (341) (0.02)<br>4. Bunn, Navalta (342) (0.02)	1. Fuller, Colwell (343) (26)<br>2. Müller, Wang (344) (22)<br>3. LeBlanc, Chaput (345) (18)<br>4. Nelson and Allen (346) (18)<br>5. Chow and Yang (287) (18)<br>6. Shrestha, Kukkonen-Harjula (347) (15)<br>7. Nelson, Low (348) (14)<br>8. Ringeval, Wagner (245) (13)<br>9. Lobelo, Kelli (349) (12)<br>10. Budig, Höltke (350) (12)

			10. Moher, Liberati (325) (4.28, 2016-2021)		
Bypass surgery<br><br>-coronary artery bypass surgery<br>-distance medicine technology<br>-electronic communication<br>-early recovery telehealth intervention<br>-high disease burden	-S=145<br>-SS=0.987<br>-MY (cited)=1997<br>-YR (ref)=1968-2008<br>-MY (citing)=2007<br>-YR (citing)= 1997-2021	1. Ware and Sherbourne (351) (25)<br>2. Krumholz, Parent (352) (24)<br>3. (353) (18)<br>4. Rosamond, Flegal (354) (18)<br>5. Vinson, Rich (355) (14)<br>6. Thompson, Buchner (356) (14)<br>7. Stewart, Marley (357) (11)<br>8. McMurray and Stewart (358) (7)<br>9. Hailey, Roine (359) (7)	1. Krumholz, Parent (352) (11.62, 2003-2013)<br>2. Shah, Der (353) (11.4, 2003-2008)<br>3. Vinson, Rich (355) (8.56, 2003-2009)<br>4. Rosamond, Flegal (354) (7.73, 2009-2015)<br>5. Stewart, Marley (357) (6.5, 2003-2010)<br>6. Ware and Sherbourne (351) (4.89, 2003-2009)<br>7. McMurray and Stewart (358) (4.41, 2006-2009)<br>8. Hailey, Roine (359) (4.31, 2004-2009)		1. Balas, Jaffrey (360) (32)<br>2. Barnason, Zimmerman (361) (30)<br>3. Barnason, Zimmerman (362) (30)<br>4. LaFramboise, Todero (363) (19)<br>5. Brennan, Moore (364) (19)<br>6. Fonarow, Abraham (365) (13)<br>7. Louis, Turner (366) (13)<br>8. Zimmerman and Barnason (367) (13)<br>9. Artinian, Harden (368) (12)<br>10. Strömberg (369) (10)
Cardiac resynchronisation therapy<br><br>-left ventricular<br>-device therapy<br>-sudden cardiac death	-S=136<br>-SS=0.927<br>-MY (ref)=2007<br>-YR (ref)= 1992-2018<br>-MY (citing)=2016<br>-YR (citing)=2001-2021	1. Brignole, Auricchio (370) (136)<br>2. Moss, Zareba (371) (133)<br>3. Bardy, Lee (372) (127)<br>4. Cleland, Daubert (373) (98)<br>5. Bristow, Saxon (374) (92)<br>6. Moss, Hall (375) (62)<br>7. Priori, Blomström-Lundqvist (376) (52)<br>8. Moss, Hall (377) (49)<br>9. Moss, Schuger (378) (44)<br>10. Tang, Wells (379) (43)	1. Epstein, Dimarco (380) (9.1, 2011-2017)<br>2. Moss, Hall (377) (5.78, 2009-2015)<br>3. Tang, Wells (379) (5.67, 2013-2017)<br>4. Abraham, Fisher (381) (5.49, 2003-2011)<br>5. McMurray, Solomon (382) (5.43, 2017-2021)<br>6. Bristow, Saxon (374) (5.2, 2009-2017)<br>7. Bardy, Lee (372) (5.02, 2008-2013)<br>8. Cleland, Daubert (373) (5.02, 2007-2015)<br>9. McAnulty, Halperin (383) (4.75, 2015-2018)<br>10. Moss, Zareba (371) (4.7, 2008-2013)	1. Bristow, Saxon (374) (0.03)	1. Hussein and Wilkoff (384) (23)<br>2. Alvarez, Cronin (162) (19)<br>3. Tseng, Kunze (385) (17)<br>4. Linde, Bongiorni (386) (16)<br>5. Sandhu, Levy (387) (15)<br>6. Gopinathannair, Cornwell (388) (14)<br>7. Ng Chee and Mela (389) (13)<br>8. Chia and Foo (390) (13)<br>9. Boriani, De Ponti (391) (12)<br>10. Saad, Hentschel (392) (120
Cardiac reactivity (gaming)<br><br>-*-physiological reactivity*<br>*-type-a-behaviour pattern*	-S=119<br>-SS=0.996<br>-MY (ref)=1981<br>-YR (ref)=1959-1990<br>-MY (citing)=1991<br>-YR (citing)=1990-1992<br><br>*Oldest*	1. Krantz and Manuck (393) (13)<br>2. Dembroski, MacDougall (394) (4)<br>3. Keys, Taylor (395) (4)<br>4. Houston, Smith (396) (4)	1. Krantz and Manuck (393) (8.74, 1990-1996)	1. Krantz and Manuck (393) (0.02)	1. Lundberg, Rasch (397) (38)<br>2. Svebak, Knardahl (398) (37)<br>3. Larkin, Zayfert (399) (34)<br>4. Larkin, Manuck (400) (27)

Heart failure telemonitoring<br><br>*-wireless telemonitoring*<br>*-principal components analysis*<br>*-cardiac abnormalities*	-S=119<br>-SS=0.989<br>-MY (ref)=2000<br>-YR (ref)=1975-2018<br>-MY (citing)=2017<br>-YR (citing)=1992-2021<br><br>*Computer based stuff*	1. Pan and Tompkins (401) (186)<br>2. Goldberger, Amaral (402) (156)<br>3. Moody and Mark (403) (78)<br>4. Oresko, Jin (404) (35)<br>5. Hochreiter and Schmidhuber (405) (29)<br>6. Kiranyaz, Ince (406) (28)<br>7. Miao, Cheng (407) (19)<br>8. Gradl, Kugler (408) (18)<br>9. Acharya, Oh (409) (17)<br>10. Luz, Schwartz (410) (17)	1. Oresko, Jin (404) (5.02, 2013-2018)	1. Pan and Tompkins (401) (0.03)	1. Factor, Gelernter (411) (30)<br>2. Mamaghanian, Khaled (412) (17)<br>3. Ibaida, Khalil (413) (16)<br>4. Mamaghanian, Khaled (414) (16)<br>5. Wasimuddin, Elleithy (264) (15)<br>6. Ryan, Sullivan (415) (12)<br>7. Faezipour, Saeed (416) (10)<br>8. Kanoun, Mamaghanian (417) (10)<br>9. Ryan, O'Sullivan (418) (9)<br>10. Faezipour, Saeed (419) (9)
Gamification<br><br>-Xbox gaming platform<br>-congestive heart failure<br>-African American<br>-organised program<br>-lifesaving treatment	-S=113<br>-SS=0.968<br>-MY (ref)=2001<br>-YR (ref)=1964-2011<br>-MY (citing)=2007<br>-YR (citing)=2004-2011	1. Adams Jr, Fonarow (420) (17)<br>2. Grady, Dracup (421) (15)<br>3. Massie and Shah (422) (10)<br>4. Haddad, Hunt (423) (10)<br>5. Rosamond, Flegal (424) (8)<br>6. Finkelstein and Cha (425) (6)<br>7. Stewart, Pearson (426) (6)	1. Grady, Dracup (421) (5.99, 2005-2012)<br>2. Massie and Shah (422) (5.73, 2005-2011)<br>3. Rosamond, Flegal (424) (4.72, 2007-2011)<br>4. Stewart, Pearson (426) (3.87, 2005-2008)		1. Finkelstein and Wood (427) (20)<br>2. Finkelstein and Wood (428) (16)<br>3. Finkelstein and Dennison (429) (14)<br>4. Finkelstein, Cha (430) (14)<br>5. Fonarow, Abraham (431) (14)<br>6. Baker, Persell (432) (13)<br>7. Kleinpell and Avitall (433) (13)<br>8. Finkelstein, Wood (434) (12)<br>9. Fonarow, Abraham (365) (12)<br>10. Müller-Nordhorn and Willich (435) (12)
Out-of-hospital cardiac arrest<br><br>-first responder<br>-cardiopulmonary resuscitation<br>-citizen responded<br>-cardiovascular care science	-S=110<br>-SS=0.988<br>-MY (ref)=2010<br>-YR (ref)=1988-2020<br>-MY (citing)=2020<br>-YR (citing)=2000-2021	1. Ringh, Rosenqvist (436) (54)<br>2. Zijlstra, Stieglis (437) (36)<br>3. Hasselqvist-Ax, Riva (438) (32)<br>4. Perkins, Handley (439) (31)<br>5. Pijls, Nelemans (440) (31)<br>6. Brooks, Simmons (441) (25)<br>7. Sasson, Rogers (442) (25)<br>8. Berglund, Claesson (443) (25)<br>9. Caputo, Muschietti (444) (24)<br>10. Monsieurs, Nolan (445) (21)	1. Brooks, Simmons (441) (7.25, 2017-2021)<br>2. Rumsfeld, Brooks (446) (5.51, 2017-2021)		1. Berg, Cheng (447) (32)<br>2. Greif, Bhanji (448) (27)<br>3. Semeraro, Greif (449) (27)<br>4. Sarkisian, Mickley (450) (27)<br>5. Scquizzato, Pallanch (451) (16)<br>6. Derkenne, Jost (452) (15)<br>7. Lyznicki, Williams (453) (15)<br>8. Matinrad, Granberg (454) (13)<br>9. Folke, Andelius (455) (13)<br>10. Metelmann, Metelmann (456) (13)
Software analysis of cardiac output<br><br>-ventricular volume<br>-quantitative gated SPECT software<br>-segmental wall motion<br>-ejection fraction	-S=108<br>-SS=0.996<br>-MY (ref)=1989<br>-YR (ref)=1958-2013<br>-MY (citing)=2007<br>-YR (citing)=1992-2020	1. Bland and Altman (457) (98)<br>2. Agatston, Janowitz (458) (10)<br>3. Germano, Kiat (459) (10)<br>4. Critchley and Critchley (460) (4)<br>5. DePuey, Nichols (461) (4)	1. Germano, Kiat (459) (5.89, 1998-2009)	1. Bland and Altman (457) (0.06)<br>2. Devereux, Pickering (462) (0.02)<br>3. Alderman, Ooi (463) (0.02)	1. Prisant, Bottini (464) (54)<br>2. Burker, Fredrikson (465) (15)<br>3. Lum and Coel (466) (7)<br>4. Knollmann, Helmig (467) (7)

Supplementary Methods Search query for WoS

TI=((heart OR cardiovascular OR cardiac OR cardiorespiratory OR cardiopulmonary OR cardiometabolic OR stroke OR myocardial OR coronary) AND (“health technology” OR “health technologies” OR “healthcare technology” OR “healthcare technologies” OR “electronic healthcare” OR “digital healthcare” OR “health tech” OR "mobile application*" OR "mobile app*" OR “digital device*” OR “electronic device*” OR “web-based” OR “web based” OR cybermedicine OR cybertherapy OR “online community” OR “online platform*” OR webinar* OR telehealth OR telemedicine OR “telehealth app*” OR “telemedicine app*” OR robot OR “videogame*” OR “video game*” OR “computer game*” OR wii OR gamification OR “immersive technology” OR VR/AR OR “immersive technologies” OR “immersive tech” OR “virtual reality” OR “virtual realities” OR “augmented reality” OR “augmented realities” OR “mixed reality” OR “mixed realities” OR “smart speaker” OR “smart device*” OR avatar* OR chatbot* OR “voice search” OR “voice activation” OR ehealth OR “electronic health” OR “digital health” OR emedicine OR “electronic medicine” OR “digital medicine” OR mhealth OR “mobile health” OR “text message” OR “text messaging” OR “text-messaging” OR “short message service” OR SMS OR “video-assisted” OR “video health” OR Twitter OR “social media” OR “Youtube” OR “Instagram” OR “facebook” OR tiktok OR smartphone* OR “smart technology” OR “smart technologies” OR “smart sensor” OR “body sensor*” OR “wireless sensor*” OR “voice technology” OR “voice technologies” OR iphone OR ipad OR “mobile phone*” OR “cell phone*” OR “android phone*” OR “telemedical” OR “health tracker*” OR “fitness tracker*” OR “fitness watch” OR “apple watch” OR fitbit OR garmin OR “activity tracker*” OR “electronic prescribing” OR “E-prescription” or eprescription* OR evisit OR “E-visit” OR “health information technology” OR “health information technologies” OR “blockchain” OR “health wearables” OR “wearables" OR “wearable devices” OR “wearable technology” OR “wearable technologies” OR telecardiology OR telemonitored OR telemonitor OR telerehabilitation OR “health monitoring system” OR “healthcare monitoring system” OR “Internet of things” OR bluetooth“ OR electronic database” OR “texting” OR “software” OR “webpage” OR “website” OR “internet-based” OR “electronic medical record*” OR “personal health record*” OR “electronic health record*” OR “health monitoring device” OR “personal monitoring device” OR “home monitoring device” OR “fitness app*” OR “sleep tracking device” OR “sleep tracking app*”)) OR

((TI=(obesity OR diabetes OR nutrition OR “physical activity” OR “physical inactivity” OR diet OR dietary OR “blood pressure” OR hypertensive OR hypertension OR smoking OR alcohol OR overweight OR sedentary OR cholesterol OR triglycerides OR lipid* OR dyslipidemia OR dyslipidaemia OR stress OR hypercholesterolaemia) AND AB=(cardiovascular or cardiac)) AND TI=(“health technology” OR “health technologies” OR “healthcare technology” OR “healthcare technologies” OR “electronic healthcare” OR “digital healthcare” OR “health tech” OR "mobile application*" OR "mobile app*" OR “digital device*” OR “electronic device*” OR “web-based” OR “web based” OR cybermedicine OR cybertherapy OR “online community” OR “online platform*” OR webinar* OR telehealth OR telemedicine OR “telehealth app*” OR “telemedicine app*” OR robot OR “videogame*” OR “video game*” OR “computer game*” OR wii OR gamification OR “immersive technology” OR VR/AR OR “immersive technologies” OR “immersive tech” OR “virtual reality” OR “virtual realities” OR “augmented reality” OR “augmented realities” OR “mixed reality” OR “mixed realities” OR “smart speaker” OR “smart device*” OR avatar* OR chatbot* OR “voice search” OR “voice activation” OR ehealth OR “electronic health” OR “digital health” OR emedicine OR “electronic medicine” OR “digital medicine” OR mhealth OR “mobile health” OR “text message” OR “text messaging” OR “text-messaging” OR “short message service” OR SMS OR “video-assisted” OR “video health” OR Twitter OR “social media” OR “Youtube” OR “Instagram” OR “facebook” OR tiktok OR smartphone* OR “smart technology” OR “smart technologies” OR “smart sensor” OR “body sensor*” OR “wireless sensor*” OR “voice technology” OR “voice technologies” OR iphone OR ipad OR “mobile phone*” OR “cell phone*” OR “android phone*” OR “telemedical” OR “health tracker*” OR “fitness tracker*” OR “fitness watch” OR “apple watch” OR fitbit OR garmin OR “activity tracker*” OR “electronic prescribing” OR “E-prescription” or eprescription* OR evisit OR “E-visit” OR “health information technology” OR “health information technologies” OR “blockchain” OR “health wearables” OR “wearables" OR “wearable devices” OR “wearable technology” OR “wearable technologies” OR telecardiology OR telemonitored OR telemonitor* OR telerehabilitation OR “health monitoring system” OR “healthcare monitoring system” OR “Internet of things” OR bluetooth“ OR electronic database” OR “texting” OR “software” OR “webpage” OR “website” OR “internet-based” OR “electronic medical record*” OR “personal health record*” OR “electronic health record*” OR “health monitoring device” OR “personal monitoring device” OR “home monitoring device” OR “fitness app*” OR “sleep tracking device” OR “sleep tracking app*”)) OR

AB=((“heart disease” OR “heart attack” OR “heart failure” OR “heart arrythmia*” OR “heart valve” OR cardiovascular OR cardiac OR cardiorespiratory OR cardiometabolic) AND (“health technology” OR “health technologies” OR “healthcare technology” OR “healthcare technologies” OR “electronic healthcare” OR “digital healthcare” OR “health tech” OR "mobile application*" OR "mobile app*" OR “digital device*” OR “electronic device*” OR “web-based” OR “web based” OR Cybermedicine OR cybertherapy OR “online community” OR “online platform*” OR telehealth OR telemedicine OR “telehealth app*” OR “telemedicine app*” OR “videogame*” OR “video game*” OR “computer game*” OR wii OR gamification OR “immersive technology” OR VR/AR OR “immersive technologies” OR “immersive tech” OR “virtual reality” OR “virtual realities” OR “augmented reality” OR “augmented realities” OR “mixed reality” OR “mixed realities” OR “smart speaker” OR “smart device*” OR avatar* OR chatbot* OR “voice search” OR “voice activation” OR ehealth OR “digital health” OR emedicine OR “digital medicine” OR mhealth OR “mobile health” OR “text message” OR “text messaging” OR “text-messaging” OR “short message service” OR SMS OR “video-assisted” OR “video health” OR Twitter OR “social media” OR “Youtube” OR “Instagram” OR tiktok OR smartphone* OR “smart technology” OR “smart technologies” OR “smart sensor” OR “body sensor*” OR “wireless sensor*” OR “voice technology” OR “voice technologies” OR iphone OR ipad OR “mobile phone*” OR “cell phone*” OR “android phone*” OR “telemedical” OR “health tracker*” OR “fitness tracker*” OR “fitness watch” OR smartwatch OR “apple watch” OR fitbit OR garmin OR “activity tracker*” OR “electronic prescribing” OR “E-prescription” or eprescription* OR evisit OR “E-visit” OR “health information technology” OR “health information technologies” OR “blockchain” OR “health wearables” OR “wearables" OR “wearable devices” OR “wearable technology” OR “wearable technologies” OR telecardiology OR telemonitored OR telemonitor* OR telerehabilitation OR “health monitoring system” OR “healthcare monitoring system” OR “Internet of things” OR Bluetooth OR “health monitoring device” OR “personal monitoring device” OR “home monitoring device” OR “fitness app*” OR “sleep tracking device” OR “sleep tracking app*” OR smartwatch)) OR

AK=((“heart disease” OR “heart attack” OR “heart failure” OR “heart arrythmia*” OR “heart valve” OR cardiovascular OR cardiac OR cardiorespiratory OR cardiometabolic) AND (“health technology” OR “health technologies” OR “healthcare technology” OR “healthcare technologies” OR “electronic healthcare” OR “digital healthcare” OR “health tech” OR "mobile application*" OR "mobile app*" OR “digital device*” OR “electronic device*” OR “web-based” OR “web based” OR Cybermedicine OR cybertherapy OR “online community” OR “online platform*” OR webinar* OR telehealth OR telemedicine OR “telehealth app*” OR “telemedicine app*” OR “videogame*” OR “video game*” OR “computer game*” OR wii OR gamification OR “immersive technology” OR VR/AR OR “immersive technologies” OR “immersive tech” OR “virtual reality” OR “virtual realities” OR “augmented reality” OR “augmented realities” OR “mixed reality” OR “mixed realities” OR “smart speaker” OR “smart device*” OR avatar* OR chatbot* OR “voice search” OR “voice activation” OR ehealth OR “electronic health” OR “digital health” OR emedicine OR “digital medicine” OR mhealth OR “mobile health” OR “text message” OR “text messaging” OR “text-messaging” OR “short message service” OR SMS OR “video-assisted” OR “video health” OR Twitter OR “social media” OR “Youtube” OR “Instagram” OR “facebook” OR tiktok OR smartphone* OR “smart technology” OR “smart technologies” OR “smart sensor” OR “body sensor*” OR “wireless sensor*” OR “voice technology” OR “voice technologies” OR iphone OR ipad OR “mobile phone*” OR “cell phone*” OR “android phone*” OR “telemedical” OR “health tracker*” OR “fitness tracker*” OR “fitness app*” OR “sleep tracking device” OR “sleep tracking app*” OR “fitness watch” OR “apple watch” OR fitbit OR garmin OR “activity tracker*” OR “electronic prescribing” OR “E-prescription” or eprescription* OR evisit OR “E-visit” OR “health information technology” OR “health information technologies” OR “blockchain” OR “health wearables” OR “wearables" OR “wearable devices” OR “wearable technology” OR “wearable technologies” OR telecardiology OR telemonitored OR telemonitor* OR telerehabilitation OR “health monitoring system” OR “healthcare monitoring system” OR “health monitoring device” OR “personal monitoring device” OR “home monitoring device” OR “Internet of things” OR Bluetooth OR smartwatch))

Supplementary References

1. Schwartz G, Tee BCK, Mei JG, Appleton AL, Kim DH, Wang HL, et al. Flexible polymer transistors with high pressure sensitivity for application in electronic skin and health monitoring. NATURE COMMUNICATIONS. 2013;4.
2. Robinson TN. Reducing children's television viewing to prevent obesity - A randomized controlled trial. JAMA-JOURNAL OF THE AMERICAN MEDICAL ASSOCIATION. 1999;282(16):1561-7.
3. Tarvainen MP, Niskanen JP, Lipponen JA, Ranta-aho PO, Karjalainen PA. Kubios HRV - Heart rate variability analysis software. COMPUTER METHODS AND PROGRAMS IN BIOMEDICINE. 2014;113(1):210-20.
4. Kann L, Kinchen S, Shanklin SL, Flint KH, Hawkins J, Harris WA, et al. Youth risk behavior surveillance—United States, 2013. Morbidity and Mortality Weekly Report: Surveillance Summaries. 2014;63(4):1-168.
5. Leitman M, Lysyansky P, Sidenko S, Shir V, Peleg E, Binenbaum M, et al. Two-dimensional strain - A novel software for real-time quantitative echocardiographic assessment of myocardial function. JOURNAL OF THE AMERICAN SOCIETY OF ECHOCARDIOGRAPHY. 2004;17(10):1021-9.
6. Brown MT, Bussell JK. Medication Adherence: WHO Cares? Mayo Clin Proc. 2011;86(4):304-14.
7. Kwakkel G, Kollen BJ, Krebs HI. Effects of robot-assisted therapy on upper limb recovery after stroke: a systematic review. Neurorehabilitation and Neural Repair. 2008;22(2):111-21.
8. Lo AC, Guarino PD, Richards LG, Haselkorn JK, Wittenberg GF, Federman DG, et al. Robot-Assisted Therapy for Long-Term Upper-Limb Impairment after Stroke. New England Journal of Medicine. 2010;362(19):1772-83.
9. Lum PS, Burgar CG, Shor PC, Majmundar M, Van der Loos M. Robot-assisted movement training compared with conventional therapy techniques for the rehabilitation of upper-limb motor function after stroke. ARCHIVES OF PHYSICAL MEDICINE AND REHABILITATION. 2002;83(7):952-9.
10. Chaudhry SI, Mattera JA, Curtis JP, Spertus JA, Herrin J, Lin Z, et al. Telemonitoring in Patients with Heart Failure. New England Journal of Medicine. 2010;363(24):2301-9.
11. Ponikowski P, Voors AA, Anker SD, Bueno H, Cleland JGF, Coats AJS, et al. 2016 ESC Guidelines for the diagnosis and treatment of acute and chronic heart failure: The Task Force for the diagnosis and treatment of acute and chronic heart failure of the European Society of Cardiology (ESC)Developed with the special contribution of the Heart Failure Association (HFA) of the ESC. European Heart Journal. 2016;37(27):2129-200.
12. Baddour LM, Epstein AE, Erickson CC, Knight BP, Levison ME, Lockhart PB, et al. Update on Cardiovascular Implantable Electronic Device Infections and Their Management A Scientific Statement From the American Heart Association. CIRCULATION. 2010;121(3):458-77.
13. Fugl-Meyer AR, Jääskö L, Leyman I, Olsson S, Steglind S. The post-stroke hemiplegic patient. 1. a method for evaluation of physical performance. Scandinavian Journal of Rehabilitation Medicine. 1975;7(1):13-31.
14. Koehler F, Winkler S, Schieber M, Sechtem U, Stangl K, Bohm M, et al. Impact of Remote Telemedical Management on Mortality and Hospitalizations in Ambulatory Patients With Chronic Heart Failure The Telemedical Interventional Monitoring in Heart Failure Study. CIRCULATION. 2011;123(17):1873-80.
15. Greenspon AJ, Patel JD, Lau E, Ochoa JA, Frisch DR, Ho RT, et al. 16-year trends in the infection burden for pacemakers and implantable cardioverter-defibrillators in the United States: 1993 to 2008. Journal of the American College of Cardiology. 2011;58(10):1001-6.
16. Abraham WT, Adamson PB, Bourge RC, Aaron MF, Costanzo MR, Stevenson LW, et al. Wireless pulmonary artery haemodynamic monitoring in chronic heart failure: a randomised controlled trial. Lancet. 2011;377(9766):658-66.
17. Cleland JG, Louis AA, Rigby AS, Janssens U, Balk AH. Noninvasive home telemonitoring for patients with heart failure at high risk of recurrent admission and death: the Trans-European Network-Home-Care Management System (TEN-HMS) study. J Am Coll Cardiol. 2005;45(10):1654-64.
18. Hindricks G, Taborsky M, Glikson M, Heinrich U, Schumacher B, Katz A, et al. Implant-based multiparameter telemonitoring of patients with heart failure (IN-TIME): a randomised controlled trial. Lancet. 2014;384(9943):583-90.
19. Wilkoff BL, Love CJ, Byrd CL, Bongiorni MG, Carrillo RG, Crossley GH, 3rd, et al. Transvenous lead extraction: Heart Rhythm Society expert consensus on facilities, training, indications, and patient management: this document was endorsed by the American Heart Association (AHA). Heart Rhythm. 2009;6(7):1085-104.

20. Inglis SC, Clark RA, McAlister FA, Ball J, Lewinter C, Cullington D, et al. Structured telephone support or telemonitoring programmes for patients with chronic heart failure. COCHRANE DATABASE OF SYSTEMATIC REVIEWS. 2010(8).
21. Goldberg LR, Piette JD, Walsh MN, Frank TA, Jaski BE, Smith AL, et al. Randomized trial of a daily electronic home monitoring system in patients with advanced heart failure: the Weight Monitoring in Heart Failure (WHARF) trial. American Heart Journal. 2003;146(4):705-12.
22. Clark RA, Inglis SC, McAlister FA, Cleland JGF, Stewart S. Telemonitoring or structured telephone support programmes for patients with chronic heart failure: systematic review and meta-analysis. BMJ. 2007;334(7600):942.
23. Klersy C, De Silvestri A, Gabutti G, Regoli F, Auricchio A. A Meta-Analysis of Remote Monitoring of Heart Failure Patients. JOURNAL OF THE AMERICAN COLLEGE OF CARDIOLOGY. 2009;54(18):1683-94.
24. Laver KE, George S, Thomas S, Deutsch JE, Crotty M. Virtual reality for stroke rehabilitation. COCHRANE DATABASE OF SYSTEMATIC REVIEWS. 2011(9).
25. Folstein MF, Folstein SE, McHugh PR. "Mini-mental state". A practical method for grading the cognitive state of patients for the clinician. J Psychiatr Res. 1975;12(3):189-98.
26. Langhorne P, Coupar F, Pollock A. Motor recovery after stroke: a systematic review. Lancet Neurol. 2009;8(8):741-54.
27. Bohannon RW, Larkin PA, Smith MB, Horton MG. Relationship between static muscle strength deficits and spasticity in stroke patients with hemiparesis. Physical therapy. 1987;67(7):1068-71.
28. Langhorne P, Bernhardt J, Kwakkel G. Stroke rehabilitation. Lancet. 2011;377(9778):1693-702.
29. Saposnik G, Teasell R, Mamdani M, Hall J, McIlroy W, Cheung D, et al. Effectiveness of Virtual Reality Using Wii Gaming Technology in Stroke Rehabilitation A Pilot Randomized Clinical Trial and Proof of Principle. STROKE. 2010;41(7):1477-84.
30. Lum PS, Burgar CG, Shor PC, Majmundar M, Van der Loos M. Robot-assisted movement training compared with conventional therapy techniques for the rehabilitation of upper-limb motor function after stroke. Arch Phys Med Rehabil. 2002;83(7):952-9.
31. Hesse S, Werner C, Pohl M, Rueckriem S, Mehrholz J, Lingnau ML. Computerized Arm Training Improves the Motor Control of the Severely Affected Arm After Stroke. Stroke. 2005;36(9):1960-6.
32. Volpe BT, Krebs HI, Hogan N, Edelstein L, Diels C, Aisen M. A novel approach to stroke rehabilitation: robot-aided sensorimotor stimulation. Neurology. 2000;54(10):1938-44.
33. Krebs HI, Hogan N, Aisen ML, Volpe BT. Robot-aided neurorehabilitation. IEEE Transactions on Rehabilitation Engineering. 1998;6(1):75-87.
34. Aisen ML, Krebs HI, Hogan N, McDowell F, Volpe BT. The effect of robot-assisted therapy and rehabilitative training on motor recovery following stroke. Arch Neurol. 1997;54(4):443-6.
35. Burgar CG, Lum PS, Shor PC, Van der Loos HFM. Development of robots for rehabilitation therapy: The Palo Alto VA/Stanford experience. Journal of rehabilitation research and development. 2000;37(6):663-74.
36. Prange GB, Jannink MJ, Groothuis-Oudshoorn CG, Hermens HJ, Ijzerman MJ. Systematic review of the effect of robot-aided therapy on recovery of the hemiparetic arm after stroke. J Rehabil Res Dev. 2006;43(2):171-84.
37. Fasoli SE, Krebs HI, Stein J, Frontera WR, Hogan N. Effects of robotic therapy on motor impairment and recovery in chronic stroke. Archives of Physical Medicine and Rehabilitation. 2003;84(4):477-82.
38. Piron L, Turolla A, Agostini M, Zucconi C, Cortese F, Zampolini M, et al. EXERCISES FOR PARETIC UPPER LIMB AFTER STROKE: A COMBINED VIRTUAL-REALITY AND TELEMEDICINE APPROACH. JOURNAL OF REHABILITATION MEDICINE. 2009;41(12):1016-20.
39. Housman SJ, Scott KM, Reinkensmeyer DJ. A randomized controlled trial of gravity-supported, computer-enhanced arm exercise for individuals with severe hemiparesis. Neurorehabil Neural Repair. 2009;23(5):505-14.
40. Volpe BT, Krebs HI, Hogan N. Robot-aided sensorimotor training in stroke rehabilitation. Adv Neurol. 2003;92:429-33.
41. Laver KE, Lange B, George S, Deutsch JE, Saposnik G, Crotty M. Virtual reality for stroke rehabilitation. Cochrane Database Syst Rev. 2017;11(11):Cd008349.

42. Basteris A, Nijenhuis SM, Stienen AHA, Buurke JH, Prange GB, Amirabdollahian F. Training modalities in robot-mediated upper limb rehabilitation in stroke: a framework for classification based on a systematic review. JOURNAL OF NEUROENGINEERING AND REHABILITATION. 2014;11.
43. Laver KE, George S, Thomas S, Deutsch JE, Crotty M. Virtual reality for stroke rehabilitation. COCHRANE DATABASE OF SYSTEMATIC REVIEWS. 2015(2).
44. Masiero S, Celia A, Armani M, Rosati G, Tavolato B, Ferraro C, et al. Robot-aided intensive training in post-stroke recovery. Aging Clinical and Experimental Research. 2006;18(3):261-5.
45. Duret C, Gracies JM. [Does upper limb robot-assisted rehabilitation contribute to improve the prognosis of post-stroke hemiparesis?]. Rev Neurol (Paris). 2014;170(11):671-9.
46. Brokaw EB, Murray T, Nef T, Lum PS. Retraining of interjoint arm coordination after stroke using robot-assisted time-independent functional training. J Rehabil Res Dev. 2011;48(4):299-316.
47. Sivan M, O'Connor RJ, Makower S, Levesley M, Bhakta B. Systematic review of outcome measures used in the evaluation of robot-assisted upper limb exercise in stroke. Journal of rehabilitation medicine. 2011;43(3):181-9.
48. Acosta AM, Dewald HA, Dewald JPA. Pilot study to test effectiveness of video game on reaching performance in stroke. Journal of rehabilitation research and development. 2011;48(4):431.
49. Mehrholz J, Hadrich A, Platz T, Kugler J, Pohl M. Electromechanical and robot-assisted arm training for improving generic activities of daily living, arm function, and arm muscle strength after stroke. COCHRANE DATABASE OF SYSTEMATIC REVIEWS. 2012(6).
50. Ong MK, Romano PS, Edgington S, Aronow HU, Auerbach AD, Black JT, et al. Effectiveness of Remote Patient Monitoring After Discharge of Hospitalized Patients With Heart Failure: The Better Effectiveness After Transition -- Heart Failure (BEAT-HF) Randomized Clinical Trial. JAMA Intern Med. 2016;176(3):310-8.
51. Inglis SC, Clark RA, McAlister FA, Stewart S, Cleland JGF. Which components of heart failure programmes are effective? A systematic review and meta-analysis of the outcomes of structured telephone support or telemonitoring as the primary component of chronic heart failure management in 8323 patients: Abridged Cochrane Review. European Journal of Heart Failure. 2011;13(9):1028-40.
52. Louis AA, Turner T, Gretton M, Baksh A, Cleland JGE. A systematic review of telemonitoring for the management of heart failure. EUROPEAN JOURNAL OF HEART FAILURE. 2003;5(5):583-90.
53. McMurray JJ, Adamopoulos S, Anker SD, Auricchio A, Böhm M, Dickstein K, et al. ESC Guidelines for the diagnosis and treatment of acute and chronic heart failure 2012: The Task Force for the Diagnosis and Treatment of Acute and Chronic Heart Failure 2012 of the European Society of Cardiology. Developed in collaboration with the Heart Failure Association (HFA) of the ESC. Eur Heart J. 2012;33(14):1787-847.
54. Roger VL, Go AS, Lloyd-Jones DM, Benjamin EJ, Berry JD, Borden WB, et al. Heart disease and stroke statistics--2012 update: a report from the American Heart Association. Circulation. 2012;125(1):e2-e220.
55. Jerant AF, Azari R, Nesbitt TS. Reducing the Cost of Frequent Hospital Admissions for Congestive Heart Failure: A Randomized Trial of a Home Telecare Intervention. Medical Care. 2001;39(11).
56. Benatar D, Bondmass M, Ghitelman J, Avitall B. Outcomes of chronic heart failure. Archives of internal medicine. 2003;163(3):347-52.
57. Artinian NT, Harden JK, Kronenberg MW, Vander Wal JS, Daher E, Stephens Q, et al. Pilot study of a Web-based compliance monitoring device for patients with congestive heart failure. Heart Lung. 2003;32(4):226-33.
58. Higgins JPT, Altman DG, Gøtzsche PC, Jüni P, Moher D, Oxman AD, et al. The Cochrane Collaboration’s tool for assessing risk of bias in randomised trials. Bmj. 2011;343.
59. Hsieh H-F, Shannon SE. Three approaches to qualitative content analysis. Qualitative health research. 2005;15(9):1277-88.
60. Finkelstein SM, Speedie SM, Demiris G, Veen M, Lundgren JM, Potthoff S. Telehomecare: quality, perception, satisfaction. Telemedicine Journal & E-Health. 2004;10(2):122-8.
61. Flodgren G, Rachas A, Farmer AJ, Inzitari M, Shepperd S. Interactive telemedicine: effects on professional practice and health care outcomes. Cochrane Database Syst Rev. 2015;2015(9):Cd002098.

62. Inglis SC, Clark RA, Dierckx R, Prieto-Merino D, Cleland JGF. Structured telephone support or non-invasive telemonitoring for patients with heart failure. COCHRANE DATABASE OF SYSTEMATIC REVIEWS. 2015(10).
63. Di Lenarda A, Casolo G, Gulizia MM, Aspromonte N, Scalvini S, Mortara A, et al. The future of telemedicine for the management of heart failure patients: a Consensus Document of the Italian Association of Hospital Cardiologists (A.N.M.C.O), the Italian Society of Cardiology (S.I.C.) and the Italian Society for Telemedicine and eHealth (Digital S.I.T.). Eur Heart J Suppl. 2017;19(Suppl D):D113-d29.
64. Aronow WS, Shamliyan TA. Comparative Effectiveness of Disease Management With Information Communication Technology for Preventing Hospitalization and Readmission in Adults With Chronic Congestive Heart Failure. J Am Med Dir Assoc. 2018;19(6):472-9.
65. Lin M-h, Yuan W-l, Huang T-c, Zhang H-f, Mai J-t, Wang J-f. Clinical effectiveness of telemedicine for chronic heart failure: a systematic review and meta-analysis. Journal of Investigative Medicine. 2017;65(5):899-911.
66. Dickinson MG, Allen LA, Albert NA, DiSalvo T, Ewald GA, Vest AR, et al. Remote Monitoring of Patients With Heart Failure: A White Paper From the Heart Failure Society of America Scientific Statements Committee. J Card Fail. 2018;24(10):682-94.
67. Banchs JE, Scher DL. Emerging role of digital technology and remote monitoring in the care of cardiac patients. Med Clin North Am. 2015;99(4):877-96.
68. Givertz MM, Stevenson LW, Costanzo MR, Bourge RC, Bauman JG, Ginn G, et al. Pulmonary Artery Pressure-Guided Management of Patients With Heart Failure and Reduced Ejection Fraction. J Am Coll Cardiol. 2017;70(15):1875-86.
69. Bertini M, Marcantoni L, Toselli T, Ferrari R. Remote monitoring of implantable devices: should we continue to ignore it? International journal of cardiology. 2016;202:368-77.
70. Chow CK, Redfern J, Hillis GS, Thakkar J, Santo K, Hackett ML, et al. Effect of Lifestyle-Focused Text Messaging on Risk Factor Modification in Patients With Coronary Heart Disease A Randomized Clinical Trial. JAMA-JOURNAL OF THE AMERICAN MEDICAL ASSOCIATION. 2015;314(12):1255-63.
71. Heran BS, Chen JMH, Ebrahim S, Moxham T, Oldridge N, Rees K, et al. Exercise-based cardiac rehabilitation for coronary heart disease. Cochrane Database of Systematic Reviews. 2011(7).
72. Varnfield M, Karunanithi M, Lee CK, Honeyman E, Arnold D, Ding H, et al. Smartphone-based home care model improved use of cardiac rehabilitation in postmyocardial infarction patients: results from a randomised controlled trial. HEART. 2014;100(22):1770-9.
73. Burke LE, Ma J, Azar KMJ, Bennett GG, Peterson ED, Zheng Y, et al. Current Science on Consumer Use of Mobile Health for Cardiovascular Disease Prevention A Scientific Statement From the American Heart Association. CIRCULATION. 2015;132(12):1157-213.
74. Craig CL, Marshall AL, Sjöström M, Bauman AE, Booth ML, Ainsworth BE, et al. International physical activity questionnaire: 12-country reliability and validity. Med Sci Sports Exerc. 2003;35(8):1381-95.
75. Kroenke K, Spitzer RL, Williams JB. The PHQ-9: validity of a brief depression severity measure. J Gen Intern Med. 2001;16(9):606-13.
76. Piepoli MF, Hoes AW, Agewall S, Albus C, Brotons C, Catapano AL, et al. 2016 European Guidelines on cardiovascular disease prevention in clinical practice: The Sixth Joint Task Force of the European Society of Cardiology and Other Societies on Cardiovascular Disease Prevention in Clinical Practice (constituted by representatives of 10 societies and by invited experts)Developed with the special contribution of the European Association for Cardiovascular Prevention & Rehabilitation (EACPR). European Heart Journal. 2016;37(29):2315-81.
77. Eysenbach G, Consort EG. CONSORT-EHEALTH: improving and standardizing evaluation reports of Web-based and mobile health interventions. J Med Internet Res. 2011;13(4):e1923.
78. Maddison R, Pfaeffli L, Whittaker R, Stewart R, Kerr A, Jiang YN, et al. A mobile phone intervention increases physical activity in people with cardiovascular disease: Results from the HEART randomized controlled trial. EUROPEAN JOURNAL OF PREVENTIVE CARDIOLOGY. 2015;22(6):701-9.
79. Free C, Phillips G, Watson L, Galli L, Felix L, Edwards P, et al. The effectiveness of mobile-health technologies to improve health care service delivery processes: a systematic review and meta-analysis. PLoS Med. 2013;10(1):e1001363.
80. Zutz A, Ignaszewski A, Bates J, Lear SA. Utilization of the internet to deliver cardiac rehabilitation at a distance: a pilot study. Telemed J E Health. 2007;13(3):323-30.

81. Chan A-W, Tetzlaff JM, Altman DG, Laupacis A, Gøtzsche PC, Krleža-Jerić K, et al. SPIRIT 2013 statement: defining standard protocol items for clinical trials. Annals of internal medicine. 2013;158(3):200-7.
82. Neubeck L, Lowres N, Benjamin EJ, Ben Freedman S, Coorey G, Redfern J. The mobile revolution-using smartphone apps to prevent cardiovascular disease. NATURE REVIEWS CARDIOLOGY. 2015;12(6):350-60.
83. Fjeldsoe BS, Marshall AL, Miller YD. Behavior change interventions delivered by mobile telephone short-message service. Am J Prev Med. 2009;36(2):165-73.
84. Thakkar J, Kurup R, Laba T-L, Santo K, Thiagalingam A, Rodgers A, et al. Mobile telephone text messaging for medication adherence in chronic disease: a meta-analysis. JAMA internal medicine. 2016;176(3):340-9.
85. Lester RT, Ritvo P, Mills EJ, Kariri A, Karanja S, Chung MH, et al. Effects of a mobile phone short message service on antiretroviral treatment adherence in Kenya (WelTel Kenya1): a randomised trial. The Lancet. 2010;376(9755):1838-45.
86. Dale LP, Whittaker R, Jiang YN, Stewart R, Rolleston A, Maddison R. Text Message and Internet Support for Coronary Heart Disease Self-Management: Results From the Text4Heart Randomized Controlled Trial. J Med Internet Res. 2015;17(10).
87. Devi R, Singh SJ, Powell J, Fulton EA, Igbinedion E, Rees K. Internet-based interventions for the secondary prevention of coronary heart disease. Cochrane Database Syst Rev. 2015(12):Cd009386.
88. Adler AJ, Martin N, Mariani J, Tajer CD, Owolabi OO, Free C, et al. Mobile phone text messaging to improve medication adherence in secondary prevention of cardiovascular disease. Cochrane Database Syst Rev. 2017;4(4):Cd011851.
89. Jin K, Khonsari S, Gallagher R, Gallagher P, Clark AM, Freedman B, et al. Telehealth interventions for the secondary prevention of coronary heart disease: A systematic review and meta-analysis. Eur J Cardiovasc Nurs. 2019;18(4):260-71.
90. Clark RA, Conway A, Poulsen V, Keech W, Tirimacco R, Tideman P. Alternative models of cardiac rehabilitation: A systematic review. EUROPEAN JOURNAL OF PREVENTIVE CARDIOLOGY. 2015;22(1):35-74.
91. Gruska M, Aigner G, Altenberger J, Burkart-Küttner D, Fiedler L, Gwechenberger M, et al. Recommendations on the utilization of telemedicine in cardiology. Wien Klin Wochenschr. 2020;132(23-24):782-800.
92. Brouwers RWM, Kraal JJ, Traa SCJ, Spee RF, Oostveen LMLC, Kemps HMC. Effects of cardiac telerehabilitation in patients with coronary artery disease using a personalised patient-centred web application: protocol for the SmartCare-CAD randomised controlled trial. BMC Cardiovascular Disorders. 2017;17(1):46.
93. Frederix I, Vanhees L, Dendale P, Goetschalckx K. A review of telerehabilitation for cardiac patients. JOURNAL OF TELEMEDICINE AND TELECARE. 2015;21(1):45-53.
94. Sohail MR, Uslan DZ, Khan AH, Friedman PA, Hayes DL, Wilson WR, et al. Management and outcome of permanent pacemaker and implantable cardioverter-defibrillator infections. Journal of the American College of Cardiology. 2007;49(18):1851-9.
95. Kusumoto FM, Schoenfeld MH, Wilkoff BL, Berul CI, Birgersdotter-Green UM, Carrillo R, et al. 2017 HRS expert consensus statement on cardiovascular implantable electronic device lead management and extraction. HEART RHYTHM. 2017;14(12):E503-+.
96. Klug D, Balde M, Pavin D, Hidden-Lucet F, Clementy J, Sadoul N, et al. Risk factors related to infections of implanted pacemakers and cardioverter-defibrillators: results of a large prospective study. Circulation. 2007;116(12):1349-55.
97. Mond HG, Proclemer A. The 11th World Survey of Cardiac Pacing and Implantable Cardioverter-Defibrillators: Calendar Year 2009–A World Society of Arrhythmia's Project. Pacing and Clinical Electrophysiology. 2011;34(8):1013-27.
98. Voigt A, Shalaby A, Saba S. Continued Rise in Rates of Cardiovascular Implantable Electronic Device Infections in the United States: Temporal Trends and Causative Insights. PACE-PACING AND CLINICAL ELECTROPHYSIOLOGY. 2010;33(4):414-9.
99. Sohail MR, Henrikson CA, Braid-Forbes MJ, Forbes KF, Lerner DJ. Mortality and Cost Associated With Cardiovascular Implantable Electronic Device Infections. Archives of Internal Medicine. 2011;171(20):1821-8.
100. Voigt A, Shalaby A, Saba S. Rising rates of cardiac rhythm management device infections in the United States: 1996 through 2003. Journal of the American College of Cardiology. 2006;48(3):590-1.

101. de Oliveira JC, Martinelli M, Nishioka SADO, Varejão T, Uipe D, Pedrosa AAA, et al. Efficacy of Antibiotic Prophylaxis Before the Implantation of Pacemakers and Cardioverter-Defibrillators. Circulation: Arrhythmia and Electrophysiology. 2009;2(1):29-34.
102. Poole JE, Gleva MJ, Mela T, Chung MK, Uslan DZ, Borge R, et al. Complication Rates Associated With Pacemaker or Implantable Cardioverter-Defibrillator Generator Replacements and Upgrade Procedures. Circulation. 2010;122(16):1553-61.
103. BLOOM H, HEEKE B, LEON A, MERA F, DELURGIO D, BESHAI J, et al. Renal Insufficiency and the Risk of Infection from Pacemaker or Defibrillator Surgery. Pacing and Clinical Electrophysiology. 2006;29(2):142-5.
104. Sohail MR, Uslan DZ, Khan AH, Friedman PA, Hayes DL, Wilson WR, et al. Infective endocarditis complicating permanent pacemaker and implantable cardioverter-defibrillator infection. Mayo Clin Proc. 2008;83(1):46-53.
105. Habib G, Lancellotti P, Antunes MJ, Bongiorni MG, Casalta J-P, Del Zotti F, et al. 2015 ESC guidelines for the management of infective endocarditis: the task force for the management of infective endocarditis of the European Society of Cardiology (ESC) endorsed by: European Association for Cardio-Thoracic Surgery (EACTS), the European Association of Nuclear Medicine (EANM). European heart journal. 2015;36(44):3075-128.
106. Chamis AL, Peterson GE, Cabell CH, Corey GR, Sorrentino RA, Greenfield RA, et al. Staphylococcus aureus bacteremia in patients with permanent pacemakers or implantable cardioverter-defibrillators. Circulation. 2001;104(9):1029-33.
107. Sohail MR, Uslan DZ, Khan AH, Friedman PA, Hayes DL, Wilson WR, et al. Risk Factor Analysis of Permanent Pacemaker Infection. Clinical Infectious Diseases. 2007;45(2):166-73.
108. Al-Khatib SM, Lucas FL, Jollis JG, Malenka DJ, Wennberg DE. The Relation Between Patients' Outcomes and the Volume of Cardioverter-Defibrillator Implantation Procedures Performed by Physicians Treating Medicare Beneficiaries. Journal of the American College of Cardiology. 2005;46(8):1536-40.
109. Boriani G, Maniadakis N, Auricchio A, Mueller-Riemenschneider F, Fattore G, Leyva F, et al. Health technology assessment in interventional electrophysiology and device therapy: a position paper of the European Heart Rhythm Association. European heart journal. 2013;34(25):1869-74.
110. DerSimonian R, Laird N. Meta-analysis in clinical trials. Controlled clinical trials. 1986;7(3):177-88.
111. Blomström-Lundqvist C, Traykov V, Erba PA, Burri H, Nielsen JC, Bongiorni MG, et al. European Heart Rhythm Association (EHRA) international consensus document on how to prevent, diagnose, and treat cardiac implantable electronic device infections-endorsed by the Heart Rhythm Society (HRS), the Asia Pacific Heart Rhythm Society (APHRS), the Latin American Heart Rhythm Society (LAHRS), International Society for Cardiovascular Infectious Diseases (ISCVID) and the European Society of Clinical Microbiology and Infectious Diseases (ESCMID) in collaboration with the European Association for Cardio-Thoracic Surgery (EACTS). Europace. 2020;22(4):515-49.
112. Padfield GJ, Steinberg C, Bennett MT, Chakrabarti S, Deyell MW, Bashir J, et al. Preventing cardiac implantable electronic device infections. Heart Rhythm. 2015;12(11):2344-56.
113. Palmeri NO, Kramer DB, Karchmer AW, Zimetbaum PJ. A Review of Cardiac Implantable Electronic Device Infections for the Practicing Electrophysiologist. JACC-CLINICAL ELECTROPHYSIOLOGY. 2021;7(6):811-24.
114. Frausing M, Kronborg MB, Johansen JB, Nielsen JC. Avoiding implant complications in cardiac implantable electronic devices: what works? Europace. 2021;23(2):163-73.
115. Polyzos KA, Konstantelias AA, Falagas ME. Risk factors for cardiac implantable electronic device infection: a systematic review and meta-analysis. Europace. 2015;17(5):767-77.
116. Palraj BR, Farid S, Sohail MR. Strategies to prevent infections associated with cardiovascular implantable electronic devices. Expert Rev Med Devices. 2017;14(5):371-81.
117. Arnold CJ, Chu VH. Cardiovascular implantable electronic device infections. Infectious Disease Clinics. 2018;32(4):811-25.
118. Han HC, Hawkins NM, Pearman CM, Birnie DH, Krahn AD. Epidemiology of cardiac implantable electronic device infections: incidence and risk factors. Europace. 2021;23(23 Suppl 4):iv3-iv10.
119. Falkner B, Kushner H, Onesti G, Angelakos ET. Cardiovascular characteristics in adolescents who develop essential hypertension. Hypertension. 1981;3(5):521-7.
120. Borghi C, Boschi S, Ambrosioni E, Melandri G, Branzi A, Magnani B. Evidence of a partial escape of renin-angiotensin-aldosterone blockade in patients with acute myocardial infarction treated with ACE inhibitors. The Journal of Clinical Pharmacology. 1993;33(1):40-5.

121. Braden DS, Leatherbury L, Treiber FA, Strong WB. Noninvasive assessment of cardiac output in children using impedance cardiography. American heart journal. 1990;120(5):1166-72.
122. Park MK, Menard SM. Accuracy of blood pressure measurement by the Dinamap monitor in infants and children. Pediatrics. 1987;79(6):907-14.
123. Anderson NB, Armstead CA. Toward understanding the association of socioeconomic status and health: A new challenge for the biopsychosocial approach. Psychosomatic medicine. 1995;57(3):213-25.
124. Anderson NB, Land JD, Taguchi F, Williams Jr RB, Houseworth SJ. Race, parental history of hypertension, and patterns of cardiovascular reactivity in women. Psychophysiology. 1989;26(1):39-47.
125. Beaglehole R, Salmond CE, Hooper A, Huntsman J, Stanhope JM, Cassel JC, et al. Blood pressure and social interaction in Tokelauan migrants in New Zealand. Journal of Chronic Diseases. 1977;30(12):803-12.
126. Lyness SA. PREDICTORS OF DIFFERENCES BETWEEN TYPE-A AND TYPE-B INDIVIDUALS IN HEART-RATE AND BLOOD-PRESSURE REACTIVITY. PSYCHOLOGICAL BULLETIN. 1993;114(2):266-95.
127. Murphy JK, Alpert BS, Walker SS, Willey ES. CHILDRENS CARDIOVASCULAR REACTIVITY - STABILITY OF RACIAL-DIFFERENCES AND RELATION TO SUBSEQUENT BLOOD-PRESSURE OVER A ONE-YEAR PERIOD. PSYCHOPHYSIOLOGY. 1991;28(4):447-57.
128. Saab PG, Tischenkel N, Spitzer SB, Gellman MD, Pasin RD, Schneiderman N. RACE AND BLOOD-PRESSURE STATUS INFLUENCES CARDIOVASCULAR-RESPONSES TO CHALLENGE. JOURNAL OF HYPERTENSION. 1991;9(3):249-58.
129. Miller SB, Sita A. PARENTAL HISTORY OF HYPERTENSION, MENSTRUAL-CYCLE PHASE, AND CARDIOVASCULAR-RESPONSE TO STRESS. PSYCHOSOMATIC MEDICINE. 1994;56(1):61-9.
130. Miller SB, Friese M, Sita A. PARENTAL HISTORY OF HYPERTENSION, SODIUM LOADING, AND CARDIOVASCULAR-RESPONSE TO STRESS. PSYCHOSOMATIC MEDICINE. 1995;57(4):381-9.
131. Miller SB. AFFECTIVE MODERATORS OF THE CARDIOVASCULAR-RESPONSE TO STRESS IN OFFSPRING OF HYPERTENSIVES. JOURNAL OF PSYCHOSOMATIC RESEARCH. 1992;36(2):149-57.
132. Sorof JM, Forman A, Cole N, Jemerin JM, Morris RC. Potassium intake and cardiovascular reactivity in children with risk factors for essential hypertension. JOURNAL OF PEDIATRICS. 1997;131(1):87-94.
133. Treiber F, Raunikar RA, Davis H, Fernandez T, Levy M, Strong WB. 1-YEAR STABILITY AND PREDICTION OF CARDIOVASCULAR FUNCTIONING AT REST AND DURING LABORATORY STRESSORS IN YOUTH WITH FAMILY HISTORIES OF ESSENTIAL-HYPERTENSION. INTERNATIONAL JOURNAL OF BEHAVIORAL MEDICINE. 1994;1(4):335-53.
134. Musante L, Raunikar RA, Treiber F, Davis H, Dysart J, Levy M, et al. CONSISTENCY OF CHILDRENS HEMODYNAMIC-RESPONSES TO LABORATORY STRESSORS. INTERNATIONAL JOURNAL OF PSYCHOPHYSIOLOGY. 1994;17(1):65-71.
135. Varma N, Epstein AE, Irimpen A, Schweikert R, Love C. Efficacy and Safety of Automatic Remote Monitoring for Implantable Cardioverter-Defibrillator Follow-Up. Circulation. 2010;122(4):325-32.
136. Healey JS, Connolly SJ, Gold MR, Israel CW, Van Gelder IC, Capucci A, et al. Subclinical Atrial Fibrillation and the Risk of Stroke. New England Journal of Medicine. 2012;366(2):120-9.
137. Slotwiner D, Varma N, Akar JG, Annas G, Beardsall M, Fogel RI, et al. HRS Expert Consensus Statement on remote interrogation and monitoring for cardiovascular implantable electronic devices. HEART RHYTHM. 2015;12(7):E69-+.
138. Saxon LA, Hayes DL, Gilliam FR, Heidenreich PA, Day J, Seth M, et al. Long-Term Outcome After ICD and CRT Implantation and Influence of Remote Device Follow-Up. Circulation. 2010;122(23):2359-67.
139. Yancy CW, Jessup M, Bozkurt B, Butler J, Casey Jr DE, Drazner MH, et al. 2013 ACCF/AHA guideline for the management of heart failure: executive summary: a report of the American College of Cardiology Foundation/American Heart Association Task Force on practice guidelines. Circulation. 2013;128(16):1810-52.

140. Landolina M, Perego GB, Lunati M, Curnis A, Guenzati G, Vicentini A, et al. Remote Monitoring Reduces Healthcare Use and Improves Quality of Care in Heart Failure Patients With Implantable Defibrillators The Evolution of Management Strategies of Heart Failure Patients With Implantable Defibrillators (EVOLVO) Study. CIRCULATION. 2012;125(24):2985-92.
141. Mabo P, Victor F, Bazin P, Ahres S, Babuty D, Da Costa A, et al. A randomized trial of long-term remote monitoring of pacemaker recipients (the COMPAS trial). Eur Heart J. 2012;33(9):1105-11.
142. Raatikainen MJ, Uusimaa P, van Ginneken MM, Janssen JP, Linnaluoto M. Remote monitoring of implantable cardioverter defibrillator patients: a safe, time-saving, and cost-effective means for follow-up. Europace. 2008;10(10):1145-51.
143. Ricci RP, Morichetli L, Santini M. Home monitoring remote control of pacemaker and implantable cardioverter defibrillator patients in clinical practice: impact on medical management and health-care resource utilization. EUROPACE. 2008;10(2):164-70.
144. Wilkoff BL, Auricchio A, Brugada J, Cowie M, Ellenbogen KA, Gillis AM, et al. HRS/EHRA expert consensus on the Monitoring of Cardiovascular Implantable Electronic Devices (CIEDs): Description of techniques, indications, personnel, frequency and ethical considerations. HEART RHYTHM. 2008;5(6):907-25.
145. Lazarus A. Remote, Wireless, Ambulatory Monitoring of Implantable Pacemakers, Cardioverter Defibrillators, and Cardiac Resynchronization Therapy Systems: Analysis of a Worldwide Database. Pacing and Clinical Electrophysiology. 2007;30(s1):S2-S12.
146. Schoenfeld MH, Compton SJ, Mead RH, Weiss DN, Sherfesee L, Englund J, et al. Remote monitoring of implantable cardioverter defibrillators: a prospective analysis. Pacing Clin Electrophysiol. 2004;27(6 Pt 1):757-63.
147. Wilkoff BL, Auricchio A, Brugada J, Cowie M, Ellenbogen KA, Gillis AM, et al. HRS/EHRA expert consensus on the monitoring of cardiovascular implantable electronic devices (CIEDs): Description of techniques, indications, personnel, frequency and ethical considerations. EUROPACE. 2008;10(6):707-25.
148. Marzegalli M, Lunati M, Landolina M, Perego GB, Ricci RP, Guenzati G, et al. Remote Monitoring of CRT-ICD: The Multicenter Italian CareLink Evaluation—Ease of Use, Acceptance, and Organizational Implications. Pacing and Clinical Electrophysiology. 2008;31(10):1259-64.
149. Fauchier L, de Bouët du Portal H, Giraudeau C, Froger S, Cosnay P, Babuty D. [Postmarketing surveillance in patients with cardiac pace-makers or automatic implantable defibrillators]. Ann Cardiol Angeiol (Paris). 2005;54(1):38-43.
150. Gladstone DJ, Spring M, Dorian P, Panzov V, Thorpe KE, Hall J, et al. Atrial Fibrillation in Patients with Cryptogenic Stroke. New England Journal of Medicine. 2014;370(26):2467-77.
151. Hindricks G, Pokushalov E, Urban L, Taborsky M, Kuck K-H, Lebedev D, et al. Performance of a New Leadless Implantable Cardiac Monitor in Detecting and Quantifying Atrial Fibrillation Results of the XPECT Trial. Circulation: Arrhythmia and Electrophysiology. 2010;3(2):141-7.
152. Adams HP, Bendixen BH, Kappelle LJ, Biller J, Love BB, Gordon DL, et al. Classification of subtype of acute ischemic stroke. Definitions for use in a multicenter clinical trial. TOAST. Trial of Org 10172 in Acute Stroke Treatment. Stroke. 1993;24(1):35-41.
153. Dubner S, Auricchio A, Steinberg JS, Vardas P, Stone P, Brugada J, et al. ISHNE/EHRA expert consensus on remote monitoring of cardiovascular implantable electronic devices (CIEDs). Ann Noninvasive Electrocardiol. 2012;17(1):36-56.
154. Flodgren G, Rachas A, Farmer AJ, Inzitari M, Shepperd S. Interactive telemedicine: effects on professional practice and health care outcomes. COCHRANE DATABASE OF SYSTEMATIC REVIEWS. 2015(9).
155. Imberti JF, Tosetti A, Mei DA, Maisano A, Boriani G. Remote monitoring and telemedicine in heart failure: implementation and benefits. Current Cardiology Reports. 2021;23(6):55.
156. Braunschweig F, Anker SD, Proff J, Varma N. Remote monitoring of implantable cardioverter-defibrillators and resynchronization devices to improve patient outcomes: dead end or way ahead? Europace. 2019;21(6):846-55.
157. Sanna T. Long-term monitoring to detect atrial fibrillation with the indwelling implantable cardiac monitors. International Journal of Stroke. 2018;13(9):893-904.
158. Heywood JT, Jermyn R, Shavelle D, Abraham WT, Bhimaraj A, Bhatt K, et al. Impact of Practice-Based Management of Pulmonary Artery Pressures in 2000 Patients Implanted With the CardioMEMS Sensor. Circulation. 2017;135(16):1509-17.

159. Lucà F, Cipolletta L, Di Fusco SA, Iorio A, Pozzi A, Rao CM, et al. Remote monitoring: Doomed to let down or an attractive promise? Int J Cardiol Heart Vasc. 2019;24:100380.
160. Freedman B, Hindricks G, Banerjee A, Baranchuk A, Ching CK, Du X, et al. World Heart Federation Roadmap on Atrial Fibrillation - A 2020 Update. Glob Heart. 2021;16(1):41.
161. Noseworthy PA, Kaufman ES, Chen LY, Chung MK, Elkind MSV, Joglar JA, et al. Subclinical and Device-Detected Atrial Fibrillation: Pondering the Knowledge Gap: A Scientific Statement From the American Heart Association. Circulation. 2019;140(25):e944-e63.
162. Alvarez CK, Cronin E, Baker WL, Kluger J. Heart failure as a substrate and trigger for ventricular tachycardia. J Interv Card Electrophysiol. 2019;56(3):229-47.
163. Lau JK, Lowres N, Neubeck L, Brieger DB, Sy RW, Galloway CD, et al. iPhone ECG application for community screening to detect silent atrial fibrillation: a novel technology to prevent stroke. Int J Cardiol. 2013;165(1):193-4.
164. Lowres N, Neubeck L, Salkeld G, Krass I, McLachlan AJ, Redfern J, et al. Feasibility and cost-effectiveness of stroke prevention through community screening for atrial fibrillation using iPhone ECG in pharmacies. Thrombosis and haemostasis. 2014;111(06):1167-76.
165. Tison GH, Sanchez JM, Ballinger B, Singh A, Olgin JE, Pletcher MJ, et al. Passive Detection of Atrial Fibrillation Using a Commercially Available Smartwatch. JAMA Cardiol. 2018;3(5):409-16.
166. Bumgarner JM, Lambert CT, Hussein AA, Cantillon DJ, Baranowski B, Wolski K, et al. Smartwatch Algorithm for Automated Detection of Atrial Fibrillation. J Am Coll Cardiol. 2018;71(21):2381-8.
167. Barrett PM, Komatireddy R, Haaser S, Topol S, Sheard J, Encinas J, et al. Comparison of 24-hour Holter monitoring with 14-day novel adhesive patch electrocardiographic monitoring. The American journal of medicine. 2014;127(1):95-e11.
168. Haberman ZC, Jahn RT, Bose R, Tun H, Shinbane JS, Doshi RN, et al. Wireless Smartphone ECG Enables Large-Scale Screening in Diverse Populations. J Cardiovasc Electrophysiol. 2015;26(5):520-6.
169. Chan PH, Wong CK, Poh YC, Pun L, Leung WWC, Wong YF, et al. Diagnostic Performance of a Smartphone-Based Photoplethysmographic Application for Atrial Fibrillation Screening in a Primary Care Setting. Journal of the American Heart Association. 2016;5(7):e003428.
170. McManus DD, Lee J, Maitas O, Esa N, Pidikiti R, Carlucci A, et al. A novel application for the detection of an irregular pulse using an iPhone 4S in patients with atrial fibrillation. Heart Rhythm. 2013;10(3):315-9.
171. Turakhia MP, Desai M, Hedlin H, Rajmane A, Talati N, Ferris T, et al. Rationale and design of a large-scale, app-based study to identify cardiac arrhythmias using a smartwatch: The Apple Heart Study. Am Heart J. 2019;207:66-75.
172. Svennberg E, Engdahl J, Al-Khalili F, Friberg L, Frykman V, Rosenqvist M. Mass Screening for Untreated Atrial Fibrillation: The STROKESTOP Study. Circulation. 2015;131(25):2176-84.
173. Scully CG, Lee J, Meyer J, Gorbach AM, Granquist-Fraser D, Mendelson Y, et al. Physiological parameter monitoring from optical recordings with a mobile phone. IEEE Trans Biomed Eng. 2012;59(2):303-6.
174. Boulos MN, Wheeler S, Tavares C, Jones R. How smartphones are changing the face of mobile and participatory healthcare: an overview, with example from eCAALYX. Biomed Eng Online. 2011;10:24.
175. Poh MZ, McDuff DJ, Picard RW. Non-contact, automated cardiac pulse measurements using video imaging and blind source separation. Opt Express. 2010;18(10):10762-74.
176. Tarakji KG, Wazni OM, Callahan T, Kanj M, Hakim AH, Wolski K, et al. Using a novel wireless system for monitoring patients after the atrial fibrillation ablation procedure: the iTransmit study. Heart Rhythm. 2015;12(3):554-9.
177. Verkruysse W, Svaasand LO, Nelson JS. Remote plethysmographic imaging using ambient light. Opt Express. 2008;16(26):21434-45.
178. Haberman ZC, Jahn RT, Bose R, Tun H, Shinbane JS, Doshi RN, et al. Wireless Smartphone ECG Enables Large-Scale Screening in Diverse Populations. JOURNAL OF CARDIOVASCULAR ELECTROPHYSIOLOGY. 2015;26(5):520-6.
179. Ades PA, Pashkow FJ, Fletcher G, Pina IL, Zohman LR, Nestor JR. A controlled trial of cardiac rehabilitation in the home setting using electrocardiographic and voice transtelephonic monitoring. Am Heart J. 2000;139(3):543-8.

180. Bruining N, Caiani E, Chronaki C, Guzik P, van der Velde E, Task Force of the e-Cardiology W. Acquisition and analysis of cardiovascular signals on smartphones: potential, pitfalls and perspectives: by the Task Force of the e-Cardiology Working Group of European Society of Cardiology. Eur J Prev Cardiol. 2014;21(2 Suppl):4-13.
181. Jonathan E, Leahy M. Investigating a smartphone imaging unit for photoplethysmography. Physiol Meas. 2010;31(11):N79-83.
182. Bosworth HB, Powers BJ, Olsen MK, McCant F, Grubber J, Smith V, et al. Home blood pressure management and improved blood pressure control: results from a randomized controlled trial. Archives of internal medicine. 2011;171(13):1173-80.
183. McManus DD, Chong JW, Soni A, Saczynski JS, Esa N, Napolitano C, et al. PULSE-SMART: pulse-based arrhythmia discrimination using a novel smartphone application. Journal of cardiovascular electrophysiology. 2016;27(1):51-7.
184. Boriani G, Laroche C, Diemberger I, Fantecchi E, Popescu MI, Rasmussen LH, et al. Asymptomatic Atrial Fibrillation: Clinical Correlates, Management, and Outcomes in the EORP-AF Pilot General Registry. The American Journal of Medicine. 2015;128(5):509-18.e2.
185. Turakhia MP, Hoang DD, Zimetbaum P, Miller JD, Froelicher VF, Kumar UN, et al. Diagnostic utility of a novel leadless arrhythmia monitoring device. Am J Cardiol. 2013;112(4):520-4.
186. Heidbuchel H, Hindricks G. The year in cardiology 2014: arrhythmias and device therapy. Eur Heart J. 2015;36(5):270-8.
187. Merchant RM, Abella BS, Abotsi EJ, Smith TM, Long JA, Trudeau ME, et al. Cell phone cardiopulmonary resuscitation: audio instructions when needed by lay rescuers: a randomized, controlled trial. Ann Emerg Med. 2010;55(6):538-43 e1.
188. Varma N, Cygankiewicz I, Turakhia M, Heidbuchel H, Hu Y, Chen LY, et al. 2021 ISHNE/ HRS/ EHRA/ APHRS collaborative statement on mHealth in Arrhythmia Management: Digital Medical Tools for Heart Rhythm Professionals: From the International Society for Holter and Noninvasive Electrocardiology/Heart Rhythm Society/European Heart Rhythm Association/Asia Pacific Heart Rhythm Society. Ann Noninvasive Electrocardiol. 2021;26(2):e12795.
189. Lopez Perales CR, Van Spall HGC, Maeda S, Jimenez A, Laţcu DG, Milman A, et al. Mobile health applications for the detection of atrial fibrillation: a systematic review. Europace. 2021;23(1):11-28.
190. Li KHC, White FA, Tipoe T, Liu T, Wong MC, Jesuthasan A, et al. The Current State of Mobile Phone Apps for Monitoring Heart Rate, Heart Rate Variability, and Atrial Fibrillation: Narrative Review. JMIR Mhealth Uhealth. 2019;7(2):e11606.
191. Tadi MJ, Mehrang S, Kaisti M, Lahdenoja O, Hurnanen T, Jaakkola J, et al. Comprehensive Analysis of Cardiogenic Vibrations for Automated Detection of Atrial Fibrillation Using Smartphone Mechanocardiograms. IEEE Sensors Journal. 2019;19(6):2230-42.
192. König S, Bollmann A, Hindricks G. Digital health solutions in the screening of subclinical atrial fibrillation. Herz. 2021;46(4):329-35.
193. Krittanawong C, Johnson KW, Rosenson RS, Wang Z, Aydar M, Baber U, et al. Deep learning for cardiovascular medicine: a practical primer. Eur Heart J. 2019;40(25):2058-73.
194. Pereira T, Tran N, Gadhoumi K, Pelter MM, Do DH, Lee RJ, et al. Photoplethysmography based atrial fibrillation detection: a review. npj Digital Medicine. 2020;3(1):3.
195. Folke F, Andelius L, Gregers MT, Hansen CM. Activation of citizen responders to out-of-hospital cardiac arrest. Curr Opin Crit Care. 2021;27(3):209-15.
196. McConnell MV, Turakhia MP, Harrington RA, King AC, Ashley EA. Mobile Health Advances in Physical Activity, Fitness, and Atrial Fibrillation: Moving Hearts. J Am Coll Cardiol. 2018;71(23):2691-701.
197. Braun V, Clarke V. Using thematic analysis in psychology. Qualitative Research in Psychology. 2006;3(2):77-101.
198. Audebert HJ, Schenkel J, Heuschmann PU, Bogdahn U, Haberl RL, Grp TE. Effects of the implementation of a telemedical stroke network: the Telemedic Pilot Project for Integrative Stroke Care (TEMPiS) in Bavaria, Germany. LANCET NEUROLOGY. 2006;5(9):742-8.
199. Go AS, Mozaffarian D, Roger VL, Benjamin EJ, Berry JD, Borden WB, et al. Heart disease and stroke statistics--2013 update: a report from the American Heart Association. Circulation. 2013;127(1):e6-e245.
200. Schwamm LH, Holloway RG, Amarenco P, Audebert HJ, Bakas T, Chumbler NR, et al. A Review of the Evidence for the Use of Telemedicine Within Stroke Systems of Care A Scientific Statement From the American Heart Association/American Stroke Association. STROKE. 2009;40(7):2616-34.

201. Meyer BC, Raman R, Hemmen T, Obler R, Zivin JA, Rao R, et al. Efficacy of site-independent telemedicine in the STRokE DOC trial: a randomised, blinded, prospective study. Lancet Neurol. 2008;7(9):787-95.
202. Marler J. Tissue plasminogen activator for acute ischemic stroke. N Engl J Med. 1995;333(24):1581-7.
203. Audebert HJ, Kukla C, von Claranau SC, Kuhn J, Vatankhah B, Schenkel J, et al. Telemedicine for safe and extended use of thrombolysis in stroke - The Telemedic Pilot Project for Integrative Stroke Care (TEMPiS) in Bavaria. STROKE. 2005;36(2):287-91.
204. Shafqat S, Kvedar JC, Guanci MM, Chang YC, Schwamm LH. Role for telemedicine in acute stroke - Feasibility and reliability of remote administration of the NIH stroke scale. STROKE. 1999;30(10):2141-5.
205. Handschu R, Littmann R, Reulbach U, Gaul C, Heckmann JG, Neundorfer B, et al. Telemedicine in emergency evaluation of acute stroke - Interrater agreement in remote video examination with a novel multimedia system. STROKE. 2003;34(12):2842-6.
206. Levine SR, Gorman M. "TeleStroke" - The application of telemedicine for stroke. STROKE. 1999;30(2):464-9.
207. Wang S, Lee SB, Pardue C, Ramsingh D, Waller J, Gross H, et al. Remote evaluation of acute ischemic stroke: reliability of National Institutes of Health Stroke Scale via telestroke. Stroke. 2003;34(10):e188-e91.
208. Wiborg A, Widder B. Teleneurology to improve stroke care in rural areas: the Telemedicine in Stroke in Swabia (TESS) Project. Stroke. 2003;34(12):2951-6.
209. LaMonte MP, Bahouth MN, Hu P, Pathan MY, Yarbrough KL, Gunawardane R, et al. Telemedicine for acute stroke - Triumphs and pitfalls. STROKE. 2003;34(3):725-8.
210. Schwamm LH, Rosenthal ES, Hirshberg A, Schaefer PW, Little EA, Kvedar JC, et al. Virtual TeleStroke support for the emergency department evaluation of acute stroke. Academic Emergency Medicine. 2004;11(11):1193-7.
211. Audebert HJ, Kukla C, Vatankhah B, Gotzler B, Schenkel J, Hofer S, et al. Comparison of tissue plasminogen activator administration management between telestroke network hospitals and academic stroke centers - The Telemedical Pilot Project for Integrative Stroke Care in Bavaria/Germany. STROKE. 2006;37(7):1822-7.
212. Schwamm LH, Holloway RG, Amarenco P, Audebert HJ, Bakas T, Chumbler NR, et al. A review of the evidence for the use of telemedicine within stroke systems of care: a scientific statement from the American Heart Association/American Stroke Association. Stroke. 2009;40(7):2616-34.
213. Demaerschalk BM, Miley ML, Kiernan TEJ, Bobrow BJ, Corday DA, Wellik KE, et al. Stroke Telemedicine. Mayo Clin Proc. 2009;84(1):53-64.
214. Audebert H, Berger K, Boy S, Einhäupl K, Endres M, Gahn G, et al. Telemedizin in der akuten Schlaganfallversorgung. Aktuelle Neurologie - AKTUEL NEUROL. 2009;36:82-90.
215. Schwamm LH, Audebert HJ, Amarenco P, Chumbler NR, Frankel MR, George MG, et al. Recommendations for the Implementation of Telemedicine Within Stroke Systems of Care. Stroke. 2009;40(7):2635-60.
216. Demaerschalk BM. Telestrokologists: Treating Stroke Patients Here, There, and Everywhere with Telemedicine. Semin Neurol. 2010;30(05):477-91.
217. Johansson T, Wild C. Telemedicine in acute stroke management: systematic review. Int J Technol Assess Health Care. 2010;26(2):149-55.
218. Pervez MA, Silva G, Masrur S, Betensky RA, Furie KL, Hidalgo R, et al. Remote Supervision of IV-tPA for Acute Ischemic Stroke by Telemedicine or Telephone Before Transfer to a Regional Stroke Center Is Feasible and Safe. Stroke. 2010;41(1):e18-e24.
219. Wechsler LR, Demaerschalk BM, Schwamm LH, Adeoye OM, Audebert HJ, Fanale CV, et al. Telemedicine Quality and Outcomes in Stroke: A Scientific Statement for Healthcare Professionals From the American Heart Association/American Stroke Association. Stroke. 2017;48(1):e3-e25.
220. Ickenstein GW, Horn M, Schenkel J, Vatankhah B, Bogdahn U, Haberl R, et al. The use of telemedicine in combination with a new stroke-code-box significantly increases t-PA use in rural communities. Neurocritical Care. 2005;3(1):27-32.
221. Shamseer L, Moher D, Clarke M, Ghersi D, Liberati A, Petticrew M, et al. Preferred reporting items for systematic review and meta-analysis protocols (PRISMA-P) 2015: elaboration and explanation. BMJ : British Medical Journal. 2015;349:g7647.
222. Chobanian AV, Bakris GL, Black HR, Cushman WC, Green LA, Izzo JJL, et al. The Seventh Report of the Joint National Committee on Prevention, Detection, Evaluation, and Treatment of High Blood PressureThe JNC 7 Report. JAMA. 2003;289(19):2560-71.
223. Green BB, Cook AJ, Ralston JD, Fishman PA, Catz SL, Carlson J, et al. Effectiveness of home blood pressure monitoring, Web communication, and pharmacist care on hypertension control: a randomized controlled trial. Jama. 2008;299(24):2857-67.

224. Morisky DE, Green LW, Levine DM. Concurrent and predictive validity of a self-reported measure of medication adherence. Med Care. 1986;24(1):67-74.
225. McManus R, Mant J, Bray EP, Holder R, Jones MI, Greenfield S, et al. Telemonitoring and self-management in the control of hypertension (TASMINH2): a randomised controlled trial. LANCET. 2010;376(9736):163-72.
226. Margolis KL, Asche SE, Bergdall AR, Dehmer SP, Groen SE, Kadrmas HM, et al. Effect of home blood pressure telemonitoring and pharmacist management on blood pressure control: a cluster randomized clinical trial. Jama. 2013;310(1):46-56.
227. Hippisley-Cox J, Coupland C, Vinogradova Y, Robson J, Minhas R, Sheikh A, et al. Predicting cardiovascular risk in England and Wales: prospective derivation and validation of QRISK2. BMJ. 2008;336(7659):1475.
228. Lewington S, Clarke R, Qizilbash N, Peto R, Collins R. Age-specific relevance of usual blood pressure to vascular mortality: a meta-analysis of individual data for one million adults in 61 prospective studies. Lancet. 2002;360(9349):1903-13.
229. Bodenheimer T, Wagner EH, Grumbach K. Improving primary care for patients with chronic illness. Jama. 2002;288(14):1775-9.
230. Wootton R. Twenty years of telemedicine in chronic disease management--an evidence synthesis. J Telemed Telecare. 2012;18(4):211-20.
231. Friedman RH, Kazis LE, Jette A, Smith MB, Stollerman J, Torgerson J, et al. A telecommunications system for monitoring and counseling patients with hypertension. Impact on medication adherence and blood pressure control. Am J Hypertens. 1996;9(4 Pt 1):285-92.
232. Rogers MA, Small D, Buchan DA, Butch CA, Stewart CM, Krenzer BE, et al. Home monitoring service improves mean arterial pressure in patients with essential hypertension. A randomized, controlled trial. Ann Intern Med. 2001;134(11):1024-32.
233. Shea S, Weinstock RS, Teresi JA, Palmas W, Starren J, Cimino JJ, et al. A randomized trial comparing telemedicine case management with usual care in older, ethnically diverse, medically underserved patients with diabetes mellitus: 5 year results of the IDEATel study. J Am Med Inform Assoc. 2009;16(4):446-56.
234. Ekeland AG, Bowes A, Flottorp S. Effectiveness of telemedicine: a systematic review of reviews. Int J Med Inform. 2010;79(11):736-71.
235. Artinian NT, Washington OGM, Templin TN. Effects of home telemonitoring and community-based monitoring on blood pressure control in urban African Americans: a pilot study. Heart & Lung. 2001;30(3):191-9.
236. McKinstry B, Hanley J, Wild S, Pagliari C, Paterson M, Lewis S, et al. Telemonitoring based service redesign for the management of uncontrolled hypertension: multicentre randomised controlled trial. BMJ : British Medical Journal. 2013;346:f3030.
237. Scherr D, Zweiker R, Kollmann A, Kastner P, Schreier G, Fruhwald FM. Mobile phone-based surveillance of cardiac patients at home. J Telemed Telecare. 2006;12(5):255-61.
238. Green BB, Cook AJ, Ralston JD, Fishman PA, Catz SL, Carlson J, et al. Effectiveness of home blood pressure monitoring, Web communication, and pharmacist care on hypertension control - A randomized controlled trial. JAMA-JOURNAL OF THE AMERICAN MEDICAL ASSOCIATION. 2008;299(24):2857-67.
239. Pickering TG, Hall JE, Appel LJ, Falkner BE, Graves J, Hill MN, et al. Recommendations for Blood Pressure Measurement in Humans and Experimental Animals. Circulation. 2005;111(5):697-716.
240. Omboni S, Ferrari R. The role of telemedicine in hypertension management: focus on blood pressure telemonitoring. Curr Hypertens Rep. 2015;17(4):535.
241. Paré G, Jaana M, Sicotte C. Systematic Review of Home Telemonitoring for Chronic Diseases: The Evidence Base. Journal of the American Medical Informatics Association. 2007;14(3):269-77.
242. Parati G, Omboni S. Role of home blood pressure telemonitoring in hypertension management: an update. Blood Press Monit. 2010;15(6):285-95.
243. Omboni S, Panzeri E, Campolo L. E-Health in Hypertension Management: an Insight into the Current and Future Role of Blood Pressure Telemonitoring. Current Hypertension Reports. 2020;22.
244. Mc Kinstry B, Hanley J, Lewis S. Telemonitoring in the management of high blood pressure. Curr Pharm Des. 2015;21(6):823-7.
245. Ringeval M, Wagner G, Denford J, Paré G, Kitsiou S. Fitbit-Based Interventions for Healthy Lifestyle Outcomes: Systematic Review and Meta-Analysis. J Med Internet Res. 2020;22(10):e23954-e.
246. Oliveira MT, Jr., Paula LJ, Marcolino MS, Canesin MF. Executive summary - guideline on telecardiology in the care of patients with acute coronary syndrome and other cardiac diseases. Arq Bras Cardiol. 2015;105(2):105-11.

247. D'Agostino RB, Vasan RS, Pencina MJ, Wolf PA, Cobain M, Massaro JM, et al. General Cardiovascular Risk Profile for Use in Primary Care. Circulation. 2008;117(6):743-53.
248. Yusuf S, Hawken S, Ôunpuu S, Dans T, Avezum A, Lanas F, et al. Effect of potentially modifiable risk factors associated with myocardial infarction in 52 countries (the INTERHEART study): case-control study. The Lancet. 2004;364(9438):937-52.
249. Charlson ME, Pompei P, Ales KL, MacKenzie CR. A new method of classifying prognostic comorbidity in longitudinal studies: development and validation. Journal of chronic diseases. 1987;40(5):373-83.
250. Lloyd-Jones DM. Cardiovascular risk prediction: basic concepts, current status, and future directions. Circulation. 2010;121(15):1768-77.
251. Wilson PWF, D'Agostino RB, Levy D, Belanger AM, Silbershatz H, Kannel WB. Prediction of coronary heart disease using risk factor categories. Circulation. 1998;97(18):1837-47.
252. Conroy RM, Pyörälä K, Fitzgerald AP, Sans S, Menotti A, De Backer G, et al. Estimation of ten-year risk of fatal cardiovascular disease in Europe: the SCORE project. European Heart Journal. 2003;24(11):987-1003.
253. Craig P, Dieppe P, Macintyre S, Michie S, Nazareth I, Petticrew M. Developing and evaluating complex interventions: the new Medical Research Council guidance. Bmj. 2008;337:a1655.
254. Grundy SM. Low-density lipoprotein, non-high-density lipoprotein, and apolipoprotein B as targets of lipid-lowering therapy. Circulation. 2002;106(20):2526-9.
255. Hannun AY, Rajpurkar P, Haghpanahi M, Tison GH, Bourn C, Turakhia MP, et al. Cardiologist-level arrhythmia detection and classification in ambulatory electrocardiograms using a deep neural network. Nat Med. 2019;25(1):65-9.
256. James PA, Oparil S, Carter BL, Cushman WC, Dennison-Himmelfarb C, Handler J, et al. 2014 Evidence-Based Guideline for the Management of High Blood Pressure in Adults: Report From the Panel Members Appointed to the Eighth Joint National Committee (JNC 8). JAMA. 2014;311(5):507-20.
257. Stone NJ, Robinson JG, Lichtenstein AH, Bairey Merz CN, Blum CB, Eckel RH, et al. 2013 ACC/AHA Guideline on the Treatment of Blood Cholesterol to Reduce Atherosclerotic Cardiovascular Risk in Adults. Circulation. 2014;129(25_suppl_2):S1-S45.
258. Cleeman JI. Executive Summary of The Third Report of The National Cholesterol Education Program (NCEP) Expert Panel on Detection, Evaluation, And Treatment of High Blood Cholesterol In Adults (Adult Treatment Panel III). Jama. 2001;285(19):2486-97.
259. Garg AX, Adhikari NK, McDonald H, Rosas-Arellano MP, Devereaux PJ, Beyene J, et al. Effects of computerized clinical decision support systems on practitioner performance and patient outcomes: a systematic review. Jama. 2005;293(10):1223-38.
260. LeCun Y, Bengio Y, Hinton G. Deep learning. nature. 2015;521(7553):436-44.
261. Cabana MD, Rand CS, Powe NR, Wu AW, Wilson MH, Abboud PA, et al. Why don't physicians follow clinical practice guidelines? A framework for improvement. Jama. 1999;282(15):1458-65.
262. Bhavnani SP, Narula J, Sengupta PP. Mobile technology and the digitization of healthcare. European Heart Journal. 2016;37(18):1428-38.
263. Pashos CL, Normand S-LT, Garfinkle JB, Newhouse JP, Epstein AM, McNeil BJ. Trends in the use of drag therapies in patients with acute myocardial infarction: 1988 to 1992. Journal of the American College of Cardiology. 1994;23(5):1023-30.
264. Wasimuddin M, Elleithy K, Abuzneid AS, Faezipour M, Abuzaghleh O. Stages-Based ECG Signal Analysis From Traditional Signal Processing to Machine Learning Approaches: A Survey. IEEE Access. 2020;8:177782-803.
265. Davis S, Abidi SS, Cox J. Personalized cardiovascular risk management linking SCORE and behaviour change to Web-based education. Stud Health Technol Inform. 2006;124:235-40.
266. Ting DSW, Peng L, Varadarajan AV, Keane PA, Burlina PM, Chiang MF, et al. Deep learning in ophthalmology: The technical and clinical considerations. PROGRESS IN RETINAL AND EYE RESEARCH. 2019;72.
267. Seetharam K, Kagiyama N, Sengupta PP. Application of mobile health, telemedicine and artificial intelligence to echocardiography. Echo Research and Practice. 2019;6(2):R41-R52.
268. Davis S, Abidi SSR, editors. Adaptive patient education framework featuring personalized cardiovascular risk management interventions2006 2006: Springer.

269. Allen J. Photoplethysmography and its application in clinical physiological measurement. Physiol Meas. 2007;28(3):R1-39.
270. Camm AJ. Heart rate variability: standards of measurement, physiological interpretation and clinical use. Task Force of the European Society of Cardiology and the North American Society of Pacing and Electrophysiology. Circulation. 1996;93(5):1043-65.
271. Pantelopoulos A, Bourbakis NG. A survey on wearable sensor-based systems for health monitoring and prognosis. IEEE Transactions on Systems, Man, and Cybernetics, Part C (Applications and Reviews). 2009;40(1):1-12.
272. Inan OT, Migeotte PF, Park KS, Etemadi M, Tavakolian K, Casanella R, et al. Ballistocardiography and seismocardiography: a review of recent advances. IEEE J Biomed Health Inform. 2015;19(4):1414-27.
273. Shaffer F, Ginsberg JP. An Overview of Heart Rate Variability Metrics and Norms. Frontiers in Public Health. 2017;5(258).
274. Rajendra Acharya U, Paul Joseph K, Kannathal N, Lim CM, Suri JS. Heart rate variability: a review. Med Biol Eng Comput. 2006;44(12):1031-51.
275. Patel S, Park H, Bonato P, Chan L, Rodgers M. A review of wearable sensors and systems with application in rehabilitation. J Neuroeng Rehabil. 2012;9:21.
276. Elgendi M. On the analysis of fingertip photoplethysmogram signals. Curr Cardiol Rev. 2012;8(1):14-25.
277. Tamura T, Maeda Y, Sekine M, Yoshida M. Wearable Photoplethysmographic Sensors—Past and Present. Electronics. 2014;3(2).
278. Schäfer A, Vagedes J. How accurate is pulse rate variability as an estimate of heart rate variability? A review on studies comparing photoplethysmographic technology with an electrocardiogram. Int J Cardiol. 2013;166(1):15-29.
279. Hamilton PS, Tompkins WJ. Quantitative investigation of QRS detection rules using the MIT/BIH arrhythmia database. IEEE Trans Biomed Eng. 1986;33(12):1157-65.
280. Lee R-G, Chen K-C, Hsiao C-C, Tseng C-L. A mobile care system with alert mechanism. IEEE Transactions on Information Technology in Biomedicine. 2007;11(5):507-17.
281. Sufi F, Fang Q, Cosic I. ECG R-R peak detection on mobile phones. Annu Int Conf IEEE Eng Med Biol Soc. 2007;2007:3697-700.
282. Hung K, Zhang Y-T. Implementation of a WAP-based telemedicine system for patient monitoring. IEEE transactions on Information Technology in Biomedicine. 2003;7(2):101-7.
283. Akselrod S, Gordon D, Ubel FA, Shannon DC, Berger AC, Cohen RJ. Power spectrum analysis of heart rate fluctuation: a quantitative probe of beat-to-beat cardiovascular control. science. 1981;213(4504):220-2.
284. Carroll D, Turner JR, Lee HJ, Stephenson J. Temporal consistency of individual differences in cardiac response to a video game. Biological Psychology. 1984;19(2):81-93.
285. Billman G. Heart Rate Variability – A Historical Perspective. Frontiers in Physiology. 2011;2.
286. Sloan RP, Bigger JT. BIOBEHAVIORAL FACTORS IN CARDIAC-ARRHYTHMIA PILOT-STUDY (CAPS) - REVIEW AND EXAMINATION. CIRCULATION. 1991;83(4):52-7.
287. Chow HW, Yang CC. Accuracy of Optical Heart Rate Sensing Technology in Wearable Fitness Trackers for Young and Older Adults: Validation and Comparison Study. JMIR Mhealth Uhealth. 2020;8(4):e14707.
288. Rapalis A, Petrėnas A, Šimaitytė M, Bailón R, Marozas V. Towards pulse rate parametrization during free-living activities using smart wristband. Physiological Measurement. 2018;39(5):055007.
289. Sufi F, Khalil I. Diagnosis of cardiovascular abnormalities from compressed ECG: a data mining-based approach. IEEE Trans Inf Technol Biomed. 2011;15(1):33-9.
290. Blum J, Rockstroh C, Göritz AS. Heart Rate Variability Biofeedback Based on Slow-Paced Breathing With Immersive Virtual Reality Nature Scenery. Frontiers in Psychology. 2019;10.
291. Sufi F, Khalil I. Enforcing secured ECG transmission for realtime telemonitoring: A joint encoding, compression, encryption mechanism. Security and Communication Networks. 2008;1(5):389-405.
292. Sufi F, Khalil I, Habib I. Cardioids-based faster authentication and diagnosis of remote cardiovascular patients. Security and Communication Networks. 2011;4(11):1351-68.
293. Sufi FK, Khalil I, Mahmood AN. A clustering based system for instant detection of cardiac abnormalities from compressed ECG. Expert Syst Appl. 2011;38(5):4705-13.
294. Gratze G, Fortin J, Holler A, Grasenick K, Pfurtscheller G, Wach P, et al. A software package for non-invasive, real-time beat-to-beat monitoring of stroke volume, blood pressure, total peripheral resistance and for assessment of autonomic function. Comput Biol Med. 1998;28(2):121-42.

295. Vogel J, Auinger A, Riedl R, Kindermann H, Helfert M, Ocenasek H. Digitally enhanced recovery: Investigating the use of digital self-tracking for monitoring leisure time physical activity of cardiovascular disease (CVD) patients undergoing cardiac rehabilitation. PLOS ONE. 2017;12(10):e0186261.
296. Indik JH, Gimbel JR, Abe H, Alkmim-Teixeira R, Birgersdotter-Green U, Clarke GD, et al. 2017 HRS expert consensus statement on magnetic resonance imaging and radiation exposure in patients with cardiovascular implantable electronic devices. Heart Rhythm. 2017;14(7):e97-e153.
297. Brignole M, Auricchio A, Baron-Esquivias G, Bordachar P, Boriani G, Breithardt OA, et al. 2013 ESC guidelines on cardiac pacing and cardiac resynchronization therapy: the task force on cardiac pacing and resynchronization therapy of the European Society of Cardiology (ESC). Developed in collaboration with the European Heart Rhythm Association (EHRA). Europace. 2013;15(8):1070-118.
298. Crossley GH, Poole JE, Rozner MA, Asirvatham SJ, Cheng A, Chung MK, et al. The Heart Rhythm Society (HRS)/American Society of Anesthesiologists (ASA) Expert Consensus Statement on the perioperative management of patients with implantable defibrillators, pacemakers and arrhythmia monitors: facilities and patient management this document was developed as a joint project with the American Society of Anesthesiologists (ASA), and in collaboration with the American Heart Association (AHA), and the Society of Thoracic Surgeons (STS). Heart Rhythm. 2011;8(7):1114-54.
299. Russo RJ, Costa HS, Silva PD, Anderson JL, Arshad A, Biederman RW, et al. Assessing the Risks Associated with MRI in Patients with a Pacemaker or Defibrillator. N Engl J Med. 2017;376(8):755-64.
300. Kalin RON, Stanton MS. Current Clinical Issues for MRI Scanning of Pacemaker and Defibrillator Patients. Pacing and Clinical Electrophysiology. 2005;28(4):326-8.
301. Hurkmans CW, Knegjens JL, Oei BS, Maas AJJ, Uiterwaal GJ, van der Borden AJ, et al. Management of radiation oncology patients with a pacemaker or ICD: a new comprehensive practical guideline in The Netherlands. Radiation oncology. 2012;7(1):1-10.
302. Nazarian S, Hansford R, Roguin A, Goldsher D, Zviman MM, Lardo AC, et al. A prospective evaluation of a protocol for magnetic resonance imaging of patients with implanted cardiac devices. Annals of internal medicine. 2011;155(7):415-24.
303. Wilkoff BL, Bello D, Taborsky M, Vymazal J, Kanal E, Heuer H, et al. Magnetic resonance imaging in patients with a pacemaker system designed for the magnetic resonance environment. Heart rhythm. 2011;8(1):65-73.
304. Beinart R, Nazarian S. Effects of external electrical and magnetic fields on pacemakers and defibrillators: from engineering principles to clinical practice. Circulation. 2013;128(25):2799-809.
305. Epstein AE, Abraham WT, Bianco NR, Kern KB, Mirro M, Rao SV, et al. Wearable cardioverter-defibrillator use in patients perceived to be at high risk early post-myocardial infarction. J Am Coll Cardiol. 2013;62(21):2000-7.
306. Levine GN, Gomes AS, Arai AE, Bluemke DA, Flamm SD, Kanal E, et al. Safety of magnetic resonance imaging in patients with cardiovascular devices: an American Heart Association scientific statement from the Committee on Diagnostic and Interventional Cardiac Catheterization, Council on Clinical Cardiology, and the Council on Cardiovascular Radiology and Intervention: endorsed by the American College of Cardiology Foundation, the North American Society for Cardiac Imaging, and the Society for Cardiovascular Magnetic Resonance. Circulation. 2007;116(24):2878-91.
307. Sommer T, Naehle CP, Yang A, Zeijlemaker V, Hackenbroch M, Schmiedel A, et al. Strategy for safe performance of extrathoracic magnetic resonance imaging at 1.5 tesla in the presence of cardiac pacemakers in non–pacemaker-dependent patients: a prospective study with 115 examinations. Circulation. 2006;114(12):1285-92.
308. Gimbel JR, Bello D, Schmitt M, Merkely B, Schwitter J, Hayes DL, et al. Randomized trial of pacemaker and lead system for safe scanning at 1.5 Tesla. Heart Rhythm. 2013;10(5):685-91.
309. Higgins JV, Gard JJ, Sheldon SH, Espinosa RE, Wood CP, Felmlee JP, et al. Safety and outcomes of magnetic resonance imaging in patients with abandoned pacemaker and defibrillator leads. Pacing and Clinical Electrophysiology. 2014;37(10):1284-90.
310. Roguin A, Schwitter J, Vahlhaus C, Lombardi M, Brugada J, Vardas P, et al. Magnetic resonance imaging in individuals with cardiovascular implantable electronic devices †. EP Europace. 2008;10(3):336-46.
311. Cohen JD, Brinton EA, Ito MK, Jacobson TA. Understanding Statin Use in America and Gaps in Patient Education (USAGE): an internet-based survey of 10,138 current and former statin users. Journal of clinical lipidology. 2012;6(3):208-15.

312. Roguin A, Zviman MM, Meininger GR, Rodrigues ER, Dickfeld TM, Bluemke DA, et al. Modern pacemaker and implantable cardioverter/defibrillator systems can be magnetic resonance imaging safe: in vitro and in vivo assessment of safety and function at 1.5 T. Circulation. 2004;110(5):475-82.
313. Indik JH, Gimbel JR, Abe H, Alkmim-Teixeira R, Birgersdotter-Green U, Clarke GD, et al. 2017 HRS expert consensus statement on magnetic resonance imaging and radiation exposure in patients with cardiovascular implantable electronic devices. HEART RHYTHM. 2017;14(7):E97-E153.
314. Yeung C, Chacko S, Glover B, Campbell D, Crystal E, Ben-Dov N, et al. Radiotherapy for patients with cardiovascular implantable electronic devices: a review. Canadian Journal of Cardiology. 2018;34(3):244-51.
315. Muthalaly RG, Nerlekar N, Ge Y, Kwong RY, Nasis A. MRI in Patients with Cardiac Implantable Electronic Devices. Radiology. 2018;289(2):281-92.
316. Nyotowidjojo IS, Skinner K, Shah AS, Bisla J, Singh S, Khoubyari R, et al. Thoracic versus nonthoracic MR imaging for patients with an MR nonconditional cardiac implantable electronic device. Pacing Clin Electrophysiol. 2018;41(6):589-96.
317. Zecchin M, Severgnini M, Fiorentino A, Malavasi VL, Menegotti L, Alongi F, et al. Management of patients with cardiac implantable electronic devices (CIED) undergoing radiotherapy: A consensus document from Associazione Italiana Aritmologia e Cardiostimolazione (AIAC), Associazione Italiana Radioterapia Oncologica (AIRO), Associazione Italiana Fisica Medica (AIFM). Int J Cardiol. 2018;255:175-83.
318. Kalb B, Indik JH, Ott P, Martin DR. MRI of patients with implanted cardiac devices. J Magn Reson Imaging. 2018;47(3):595-603.
319. Jung W, Jäckle S, Zvereva V. MRI and implantable cardiac electronic devices. Curr Opin Cardiol. 2015;30(1):65-73.
320. Ipek EG, Nazarian S. Safety of Implanted Cardiac Devices in an MRI Environment. Current Cardiology Reports. 2015;17(7):51.
321. Shah AD, Patel AU, Knezevic A, Hoskins MH, Hirsh DS, Merchant FM, et al. Clinical Performance of Magnetic Resonance Imaging Conditional and Nonconditional Cardiac Implantable Electronic Devices. Pacing and Clinical Electrophysiology. 2017;40(5):467-75.
322. Miften M, Mihailidis D, Kry SF, Reft C, Esquivel C, Farr J, et al. Management of radiotherapy patients with implanted cardiac pacemakers and defibrillators: A Report of the AAPM TG-203(†). Med Phys. 2019;46(12):e757-e88.
323. Widmer RJ, Collins NM, Collins CS, West CP, Lerman LO, Lerman A. Digital Health Interventions for the Prevention of Cardiovascular Disease: A Systematic Review and Meta-analysis. Mayo Clin Proc. 2015;90(4):469-80.
324. Garber CE, Blissmer B, Deschenes MR, Franklin BA, Lamonte MJ, Lee IM, et al. American College of Sports Medicine position stand. Quantity and quality of exercise for developing and maintaining cardiorespiratory, musculoskeletal, and neuromotor fitness in apparently healthy adults: guidance for prescribing exercise. Medicine and science in sports and exercise. 2011;43(7):1334-59.
325. Moher D, Liberati A, Tetzlaff J, Altman DG. Preferred reporting items for systematic reviews and meta-analyses: the PRISMA statement. Int J Surg. 2010;8(5):336-41.
326. Bravata DM, Smith-Spangler C, Sundaram V, Gienger AL, Lin N, Lewis R, et al. Using pedometers to increase physical activity and improve health: a systematic review. Jama. 2007;298(19):2296-304.
327. Shcherbina A, Mattsson CM, Waggott D, Salisbury H, Christle JW, Hastie T, et al. Accuracy in wrist-worn, sensor-based measurements of heart rate and energy expenditure in a diverse cohort. Journal of personalized medicine. 2017;7(2):3.
328. Egger M, Smith GD, Schneider M, Minder C. Bias in meta-analysis detected by a simple, graphical test. BMJ. 1997;315(7109):629.
329. Haskell WL, Lee IM, Pate RR, Powell KE, Blair SN, Franklin BA, et al. Physical activity and public health: updated recommendation for adults from the American College of Sports Medicine and the American Heart Association. Med Sci Sports Exerc. 2007;39(8):1423-34.
330. Wallen MP, Gomersall SR, Keating SE, Wisløff U, Coombes JS. Accuracy of Heart Rate Watches: Implications for Weight Management. PLoS One. 2016;11(5):e0154420.
331. Martin SS, Feldman DI, Blumenthal RS, Jones SR, Post WS, McKibben RA, et al. mActive: A Randomized Clinical Trial of an Automated mHealth Intervention for Physical Activity Promotion. J Am Heart Assoc. 2015;4(11).
332. Evenson KR, Goto MM, Furberg RD. Systematic review of the validity and reliability of consumer-wearable activity trackers. International Journal of Behavioral Nutrition and Physical Activity. 2015;12(1):159.
333. Higgins J. Convincing evidence from controlled and uncontrolled studies on the lipid-lowering effect of a statin. Cochrane Database Syst Rev. 2012(12):Ed000049.

334. Graves LEF, Ridgers ND, Williams K, Stratton G, Atkinson GT. The physiological cost and enjoyment of Wii Fit in adolescents, young adults, and older adults. Journal of physical activity & health. 2010;7(3):393-401.
335. Deutsch JE, Borbely M, Filler J, Huhn K, Guarrera-Bowlby P. Use of a low-cost, commercially available gaming console (Wii) for rehabilitation of an adolescent with cerebral palsy. Phys Ther. 2008;88(10):1196-207.
336. Germano G, Hoes A, Karadeniz S, Mezzani A, Prescott E, Ryden L, et al. European Guidelines on cardiovascular disease prevention in clinical practice (version 2012). Eur Heart J. 2012;33:1635-701.
337. Lanningham-Foster L, Foster RC, McCrady SK, Jensen TB, Mitre N, Levine JA. Activity-promoting video games and increased energy expenditure. J Pediatr. 2009;154(6):819-23.
338. Ferguson T, Rowlands AV, Olds T, Maher C. The validity of consumer-level, activity monitors in healthy adults worn in free-living conditions: a cross-sectional study. International journal of behavioral nutrition and physical activity. 2015;12(1):1-9.
339. Graf DL, Pratt LV, Hester CN, Short KR. Playing active video games increases energy expenditure in children. Pediatrics. 2009;124(2):534-40.
340. Wang R, Blackburn G, Desai M, Phelan D, Gillinov L, Houghtaling P, et al. Accuracy of wrist-worn heart rate monitors. Jama cardiology. 2017;2(1):104-6.
341. Dooley EE, Golaszewski NM, Bartholomew JB. Estimating accuracy at exercise intensities: a comparative study of self-monitoring heart rate and physical activity wearable devices. JMIR mHealth and uHealth. 2017;5(3):e7043.
342. Bunn JA, Navalta JW, Fountaine CJ, Reece JD. Current state of commercial wearable technology in physical activity monitoring 2015–2017. International journal of exercise science. 2018;11(7):503.
343. Fuller D, Colwell E, Low J, Orychock K, Tobin MA, Simango B, et al. Reliability and Validity of Commercially Available Wearable Devices for Measuring Steps, Energy Expenditure, and Heart Rate: Systematic Review. JMIR Mhealth Uhealth. 2020;8(9):e18694.
344. Müller AM, Wang NX, Yao J, Tan CS, Low ICC, Lim N, et al. Heart Rate Measures From Wrist-Worn Activity Trackers in a Laboratory and Free-Living Setting: Validation Study. JMIR Mhealth Uhealth. 2019;7(10):e14120.
345. LeBlanc AG, Chaput JP, McFarlane A, Colley RC, Thivel D, Biddle SJH, et al. Active Video Games and Health Indicators in Children and Youth: A Systematic Review. PLOS ONE. 2013;8(6).
346. Nelson BW, Allen NB. Accuracy of Consumer Wearable Heart Rate Measurement During an Ecologically Valid 24-Hour Period: Intraindividual Validation Study. JMIR Mhealth Uhealth. 2019;7(3):e10828.
347. Shrestha N, Kukkonen-Harjula KT, Verbeek JH, Ijaz S, Hermans V, Pedisic Z. Workplace interventions for reducing sitting at work. Cochrane Database Syst Rev. 2018;6(6):Cd010912.
348. Nelson BW, Low CA, Jacobson N, Areán P, Torous J, Allen NB. Guidelines for wrist-worn consumer wearable assessment of heart rate in biobehavioral research. npj Digital Medicine. 2020;3(1):90.
349. Lobelo F, Kelli HM, Tejedor SC, Pratt M, McConnell MV, Martin SS, et al. The Wild Wild West: A Framework to Integrate mHealth Software Applications and Wearables to Support Physical Activity Assessment, Counseling and Interventions for Cardiovascular Disease Risk Reduction. Prog Cardiovasc Dis. 2016;58(6):584-94.
350. Budig M, Höltke V, Keiner M. Accuracy of optical heart rate measurement and distance measurement of a fitness tracker and their consequential use in sports. German Journal of Exercise and Sport Research. 2019;49:402-9.
351. Ware JE, Jr., Sherbourne CD. The MOS 36-item short-form health survey (SF-36). I. Conceptual framework and item selection. Med Care. 1992;30(6):473-83.
352. Krumholz HM, Parent EM, Tu N, Vaccarino V, Wang Y, Radford MJ, et al. Readmission after hospitalization for congestive heart failure among Medicare beneficiaries. Arch Intern Med. 1997;157(1):99-104.
353. Shah NB, Der E, Ruggerio C, Heidenreich PA, Massie BM. Prevention of hospitalizations for heart failure with an interactive home monitoring program. Am Heart J. 1998;135(3):373-8.

354. Rosamond W, Flegal K, Furie K, Go A, Greenlund K, Haase N, et al. Heart disease and stroke statistics--2008 update: a report from the American Heart Association Statistics Committee and Stroke Statistics Subcommittee. Circulation. 2008;117(4):e25-146.
355. Vinson JM, Rich MW, Sperry JC, Shah AS, McNamara T. Early readmission of elderly patients with congestive heart failure. J Am Geriatr Soc. 1990;38(12):1290-5.
356. Thompson PD, Buchner D, Pina IL, Balady GJ, Williams MA, Marcus BH, et al. Exercise and physical activity in the prevention and treatment of atherosclerotic cardiovascular disease: a statement from the Council on Clinical Cardiology (Subcommittee on Exercise, Rehabilitation, and Prevention) and the Council on Nutrition, Physical Activity, and Metabolism (Subcommittee on Physical Activity). Circulation. 2003;107(24):3109-16.
357. Stewart S, Marley JE, Horowitz JD. Effects of a multidisciplinary, home-based intervention on planned readmissions and survival among patients with chronic congestive heart failure: a randomised controlled study. The Lancet. 1999;354(9184):1077-83.
358. McMurray JJ, Stewart S. Epidemiology, aetiology, and prognosis of heart failure. Heart. 2000;83(5):596-602.
359. Hailey D, Roine R, Ohinmaa A. Systematic review of evidence for the benefits of telemedicine. J Telemed Telecare. 2002;8 Suppl 1:1-30.
360. Balas EA, Jaffrey F, Kuperman GJ, Boren SA, Brown GD, Pinciroli F, et al. Electronic communication with patients - Evaluation of distance medicine technology. JAMA-JOURNAL OF THE AMERICAN MEDICAL ASSOCIATION. 1997;278(2):152-9.
361. Barnason S, Zimmerman L, Schulz P, Tu C. Influence of an early recovery telehealth intervention on physical activity and functioning after coronary artery bypass surgery among older adults with high disease burden. Heart & Lung. 2009;38(6):459-68.
362. Barnason S, Zimmerman L, Nieveen J, Schulz P, Miller C, Hertzog M, et al. Influence of a symptom management telehealth intervention on older adults' early recovery outcomes after coronary artery bypass surgery. Heart Lung. 2009;38(5):364-76.
363. LaFramboise LM, Todero CM, Zimmerman L, Agrawal S. Comparison of Health Buddy with traditional approaches to heart failure management. Fam Community Health. 2003;26(4):275-88.
364. Brennan PF, Moore SM, Bjornsdottir G, Jones J, Visovsky C, Rogers M. HeartCare: an Internet-based information and support system for patient home recovery after coronary artery bypass graft (CABG) surgery. J Adv Nurs. 2001;35(5):699-708.
365. Fonarow GC, Abraham WT, Albert NM, Stough WG, Gheorghiade M, Greenberg BH, et al. Carvedilol use at discharge in patients hospitalized for heart failure is associated with improved survival: an analysis from Organized Program to Initiate Lifesaving Treatment in Hospitalized Patients with Heart Failure (OPTIMIZE-HF). Am Heart J. 2007;153(1):82.e1-11.
366. Louis AA, Turner T, Gretton M, Baksh A, Cleland JG. A systematic review of telemonitoring for the management of heart failure. Eur J Heart Fail. 2003;5(5):583-90.
367. Zimmerman L, Barnason S. Use of a telehealth device to deliver a symptom management intervention to cardiac surgical patients. J Cardiovasc Nurs. 2007;22(1):32-7.
368. Artinian NT, Harden JK, Kronenberg MW, Vander Wal JS, Daher E, Stephens Q, et al. Pilot study of a Web-based compliance monitoring device for patients with congestive heart failure. HEART & LUNG. 2003;32(4):226-33.
369. Strömberg A. The crucial role of patient education in heart failure. Eur J Heart Fail. 2005;7(3):363-9.
370. Brignole M, Auricchio A, Baron-Esquivias G, Bordachar P, Boriani G, Breithardt OA, et al. 2013 ESC Guidelines on cardiac pacing and cardiac resynchronization therapy: the Task Force on cardiac pacing and resynchronization therapy of the European Society of Cardiology (ESC). Developed in collaboration with the European Heart Rhythm Association (EHRA). Eur Heart J. 2013;34(29):2281-329.
371. Moss AJ, Zareba W, Hall WJ, Klein H, Wilber DJ, Cannom DS, et al. Prophylactic Implantation of a Defibrillator in Patients with Myocardial Infarction and Reduced Ejection Fraction. New England Journal of Medicine. 2002;346(12):877-83.
372. Bardy GH, Lee KL, Mark DB, Poole JE, Packer DL, Boineau R, et al. Amiodarone or an Implantable Cardioverter–Defibrillator for Congestive Heart Failure. New England Journal of Medicine. 2005;352(3):225-37.
373. Cleland JG, Daubert JC, Erdmann E, Freemantle N, Gras D, Kappenberger L, et al. The effect of cardiac resynchronization on morbidity and mortality in heart failure. N Engl J Med. 2005;352(15):1539-49.

374. Bristow MR, Saxon LA, Boehmer J, Krueger S, Kass DA, De Marco T, et al. Cardiac-resynchronization therapy with or without an implantable defibrillator in advanced chronic heart failure. N Engl J Med. 2004;350(21):2140-50.
375. Moss AJ, Hall WJ, Cannom DS, Klein H, Brown MW, Daubert JP, et al. Cardiac-resynchronization therapy for the prevention of heart-failure events. N Engl J Med. 2009;361(14):1329-38.
376. Priori SG, Blomström-Lundqvist C, Mazzanti A, Blom N, Borggrefe M, Camm J, et al. 2015 ESC Guidelines for the management of patients with ventricular arrhythmias and the prevention of sudden cardiac death: The Task Force for the Management of Patients with Ventricular Arrhythmias and the Prevention of Sudden Cardiac Death of the European Society of Cardiology (ESC)Endorsed by: Association for European Paediatric and Congenital Cardiology (AEPC). Europace. 2015;17(11):1601-87.
377. Moss AJ, Hall WJ, Cannom DS, Daubert JP, Higgins SL, Klein H, et al. Improved survival with an implanted defibrillator in patients with coronary disease at high risk for ventricular arrhythmia. Multicenter Automatic Defibrillator Implantation Trial Investigators. N Engl J Med. 1996;335(26):1933-40.
378. Moss AJ, Schuger C, Beck CA, Brown MW, Cannom DS, Daubert JP, et al. Reduction in inappropriate therapy and mortality through ICD programming. N Engl J Med. 2012;367(24):2275-83.
379. Tang AS, Wells GA, Talajic M, Arnold MO, Sheldon R, Connolly S, et al. Cardiac-resynchronization therapy for mild-to-moderate heart failure. N Engl J Med. 2010;363(25):2385-95.
380. Epstein AE, Dimarco JP, Ellenbogen KA, Estes NA, 3rd, Freedman RA, Gettes LS, et al. ACC/AHA/HRS 2008 Guidelines for device-based therapy of cardiac rhythm abnormalities. Heart Rhythm. 2008;5(6):e1-62.
381. Abraham WT, Fisher WG, Smith AL, Delurgio DB, Leon AR, Loh E, et al. Cardiac resynchronization in chronic heart failure. New England Journal of Medicine. 2002;346(24):1845-53.
382. McMurray JJV, Solomon SD, Inzucchi SE. DAPA-HF. N Engl J Med. 2019;381(21):1995-2008.
383. McAnulty J, Halperin B, Kron J, Larsen G, Rait M, Swenson R, et al. A comparison of antiarrhythmic-drug therapy with implantable defibrillators in patients resuscitated from near-fatal ventricular arrhythmias. New England Journal of Medicine. 1997;337(22):1576-83.
384. Hussein AA, Wilkoff BL. Cardiac Implantable Electronic Device Therapy in Heart Failure. Circ Res. 2019;124(11):1584-97.
385. Tseng AS, Kunze KL, Lee JZ, Amin M, Neville MR, Almader-Douglas D, et al. Efficacy of Pharmacologic and Cardiac Implantable Electronic Device Therapies in Patients With Heart Failure and Reduced Ejection Fraction. Circulation: Arrhythmia and Electrophysiology. 2019;12(6):e006951.
386. Linde C, Bongiorni MG, Birgersdotter-Green U, Curtis AB, Deisenhofer I, Furokawa T, et al. Sex differences in cardiac arrhythmia: a consensus document of the European Heart Rhythm Association, endorsed by the Heart Rhythm Society and Asia Pacific Heart Rhythm Society. EP Europace. 2018;20(10):1565-ao.
387. Sandhu A, Levy A, Varosy PD, Matlock D. Implantable Cardioverter-Defibrillators and Cardiac Resynchronization Therapy in Older Adults With Heart Failure. J Am Geriatr Soc. 2019;67(10):2193-9.
388. Gopinathannair R, Cornwell WK, Dukes JW, Ellis CR, Hickey KT, Joglar JA, et al. Device Therapy and Arrhythmia Management in Left Ventricular Assist Device Recipients: A Scientific Statement From the American Heart Association. Circulation. 2019;139(20):e967-e89.
389. Ng Chee Y, Mela T. A Primer on Cardiac Devices: Psychological and Pharmacological Considerations. Psychiatric Annals. 2016;46(12):683-90.
390. Chia PL, Foo D. Overview of implantable cardioverter defibrillator and cardiac resynchronisation therapy in heart failure management. Singapore Med J. 2016;57(7):354-9.
391. Boriani G, De Ponti R, Guerra F, Palmisano P, Zanotto G, D'Onofrio A, et al. Sinergy between drugs and devices in the fight against sudden cardiac death and heart failure. European Journal of Preventive Cardiology. 2021;28(1):110-23.
392. Saad TF, Hentschel DM, Koplan B, Wasse H, Asif A, Patel DV, et al. Cardiovascular implantable electronic device leads in CKD and ESRD patients: review and recommendations for practice. Semin Dial. 2013;26(1):114-23.
393. Krantz DS, Manuck SB. Acute psychophysiologic reactivity and risk of cardiovascular disease: a review and methodologic critique. Psychological bulletin. 1984;96(3):435.
394. Dembroski TM, MacDougall JM, Shields JL, Petitto J, Lushene R. Components of the Type A coronary-prone behavior pattern and cardiovascular responses to psychomotor performance challenge. Journal of Behavioral Medicine. 1978;1(2):159-76.

395. Keys A, Taylor HL, Blackburn H, Brozek J, Anderson JT, Simonson E. Mortality and coronary heart disease among men studied for 23 years. Arch Intern Med. 1971;128(2):201-14.
396. Houston BK, Smith TW, Zurawski RM. Principal dimensions of the Framingham Type A scale: Differential relationships to cardiovascular reactivity and anxiety. Journal of Human Stress. 1986;12(3):105-12.
397. Lundberg U, Rasch B, Westermark O. Physiological Reactivity and Type A Behavior in Preschool Children: A Longitudinal Study. Behavioral Medicine. 1991;17(4):149-57.
398. Svebak S, Knardahl S, Nordby H, Aakvaag A. Components of type A behavior pattern as predictors of neuroendocrine and cardiovascular reactivity in challenging tasks. Personality and Individual Differences. 1992;13(6):733-44.
399. Larkin KT, Zayfert C, Veltum LG, Abel JL. Effects of feedback and contingent reinforcement in reducing heart rate response to stress. Journal of Psychophysiology. 1992;6(2):119-30.
400. Larkin KT, Manuck SB, Kasprowicz AL. The effect of feedback-assisted reduction in heart rate reactivity on videogame performance. Biofeedback & Self Regulation. 1990;15(4):285-303.
401. Pan J, Tompkins WJ. A real-time QRS detection algorithm. IEEE Trans Biomed Eng. 1985;32(3):230-6.
402. Goldberger AL, Amaral LAN, Glass L, Hausdorff JM, Ivanov PC, Mark RG, et al. PhysioBank, PhysioToolkit, and PhysioNet: components of a new research resource for complex physiologic signals. circulation. 2000;101(23):e215-e20.
403. Moody GB, Mark RG. The impact of the MIT-BIH Arrhythmia Database. IEEE Engineering in Medicine and Biology Magazine. 2001;20(3):45-50.
404. Oresko JJ, Jin Z, Cheng J, Huang S, Sun Y, Duschl H, et al. A wearable smartphone-based platform for real-time cardiovascular disease detection via electrocardiogram processing. IEEE Transactions on Information Technology in Biomedicine. 2010;14(3):734-40.
405. Hochreiter S, Schmidhuber J. Long Short-Term Memory. Neural Comput. 1997;9(8):1735–80.
406. Kiranyaz S, Ince T, Gabbouj M. Real-time patient-specific ECG classification by 1-D convolutional neural networks. IEEE Transactions on Biomedical Engineering. 2015;63(3):664-75.
407. Miao F, Cheng Y, He Y, He Q, Li Y. A Wearable Context-Aware ECG Monitoring System Integrated with Built-in Kinematic Sensors of the Smartphone. Sensors. 2015;15(5).
408. Gradl S, Kugler P, Lohmüller C, Eskofier B, editors. Real-time ECG monitoring and arrhythmia detection using Android-based mobile devices2012: IEEE.
409. Acharya UR, Oh SL, Hagiwara Y, Tan JH, Adam M, Gertych A, et al. A deep convolutional neural network model to classify heartbeats. Computers in biology and medicine. 2017;89:389-96.
410. Luz EJdS, Schwartz WR, Cámara-Chávez G, Menotti D. ECG-based heartbeat classification for arrhythmia detection: A survey. Computer methods and programs in biomedicine. 2016;127:144-64.
411. Factor M, Gelernter DH, Sittig DF. The Multi-Trellis Software Architecture and the Intelligent Cardiovascular Monitor. Methods Inf Med. 1992;31(01):44-55.
412. Mamaghanian H, Khaled N, Atienza D, Vandergheynst P. Compressed Sensing for Real-Time Energy-Efficient ECG Compression on Wireless Body Sensor Nodes. IEEE Transactions on Biomedical Engineering. 2011;58(9):2456-66.
413. Ibaida A, Khalil I, Sufi F, editors. Cardiac abnormalities detection from compressed ECG in wireless telemonitoring using principal components analysis (PCA). 2009 International Conference on Intelligent Sensors, Sensor Networks and Information Processing (ISSNIP); 2009 7-10 Dec. 2009.
414. Mamaghanian H, Khaled N, Atienza D, Vandergheynst P, editors. Real-time compressed sensing-based electrocardiogram compression on energy-constrained wireless body sensors. 2011 IEEE International Symposium of Circuits and Systems (ISCAS); 2011 15-18 May 2011.
415. Ryan J, Sullivan C, Bell C, O'Sullivan C. Real-time interactive volumetric animation of the heart's electrical cycle from automatically synchronized ECG. Computer Animation and Virtual Worlds. 2004;15:353-60.
416. Faezipour M, Saeed A, Bulusu SC, Nourani M, Minn H, Tamil L. A patient-adaptive profiling scheme for ECG beat classification. IEEE Trans Inf Technol Biomed. 2010;14(5):1153-65.

417. Kanoun K, Mamaghanian H, Khaled N, Atienza D, editors. A real-time compressed sensing-based personal electrocardiogram monitoring system. 2011 Design, Automation & Test in Europe; 2011 14-18 March 2011.
418. Ryan J, O'Sullivan C, Bell C, Mooney R, editors. A virtual reality electrocardiography teaching tool2004.
419. Faezipour M, Saeed A, Nourani M, editors. Automated ECG profiling and beat classification. 2010 IEEE International Conference on Acoustics, Speech and Signal Processing; 2010 14-19 March 2010.
420. Adams Jr KF, Fonarow GC, Emerman CL, LeJemtel TH, Costanzo MR, Abraham WT, et al. Characteristics and outcomes of patients hospitalized for heart failure in the United States: rationale, design, and preliminary observations from the first 100,000 cases in the Acute Decompensated Heart Failure National Registry (ADHERE). American heart journal. 2005;149(2):209-16.
421. Grady KL, Dracup K, Kennedy G, Moser DK, Piano M, Stevenson LW, et al. Team management of patients with heart failure: A statement for healthcare professionals from The Cardiovascular Nursing Council of the American Heart Association. Circulation. 2000;102(19):2443-56.
422. Massie BM, Shah NB. Evolving trends in the epidemiologic factors of heart failure: rationale for preventive strategies and comprehensive disease management. American heart journal. 1997;133(6):703-12.
423. Haddad F, Hunt SA, Rosenthal DN, Murphy DJ. Right ventricular function in cardiovascular disease, part I: anatomy, physiology, aging, and functional assessment of the right ventricle. Circulation. 2008;117(11):1436-48.
424. Rosamond W, Flegal K, Friday G, Furie K, Go A, Greenlund K, et al. Heart disease and stroke statistics--2007 update: a report from the American Heart Association Statistics Committee and Stroke Statistics Subcommittee. Circulation. 2007;115(5):e69-171.
425. Finkelstein J, Cha E. Hypertension Telemanagement in Blacks. Circulation: Cardiovascular Quality and Outcomes. 2009;2(3):272-8.
426. Stewart S, Pearson S, Horowitz JD. Effects of a home-based intervention among patients with congestive heart failure discharged from acute hospital care. Archives of Internal Medicine. 1998;158(10):1067-72.
427. Finkelstein J, Wood J. Implementing home telemanagement of congestive heart failure using Xbox gaming platform. Annu Int Conf IEEE Eng Med Biol Soc. 2011;2011:3158-63.
428. Finkelstein J, Wood J. Delivering chronic heart failure telemanagement via multiple interactive platforms. management. 2013;6:8.
429. Finkelstein J, Dennison CR, editors. A pilot study of home automated telemanagement (HAT) system in African Americans with Congestive Heart Failure2010: IEEE.
430. Finkelstein J, Cha E, Dennison CR. Exploring feasibility of home telemanagement in African Americans with congestive heart failure. Stud Health Technol Inform. 2010;160(Pt 1):535-9.
431. Fonarow GC, Abraham WT, Albert NM, Gattis Stough W, Gheorghiade M, Greenberg BH, et al. Influence of a Performance-Improvement Initiative on Quality of Care for Patients Hospitalized With Heart Failure: Results of the Organized Program to Initiate Lifesaving Treatment in Hospitalized Patients With Heart Failure (OPTIMIZE-HF). Archives of Internal Medicine. 2007;167(14):1493-502.
432. Baker DW, Persell SD, Thompson JA, Soman NS, Burgner KM, Liss D, et al. Automated Review of Electronic Health Records to Assess Quality of Care for Outpatients with Heart Failure. Annals of Internal Medicine. 2007;146(4):270-7.
433. Kleinpell RM, Avitall B. Telemanagement in Chronic Heart Failure. Disease Management & Health Outcomes. 2005;13(1):43-52.
434. Finkelstein J, Wood J, Cha E, Orlov A, Dennison C, editors. Feasibility of congestive heart failure telemanagement using a Wii-based telecare platform2010: IEEE.
435. Müller-Nordhorn J, Willich SN. Effectiveness of interventions to increase adherence to statin therapy. Disease Management & Health Outcomes. 2005;13(2):73-82.
436. Ringh M, Rosenqvist M, Hollenberg J, Jonsson M, Fredman D, Nordberg P, et al. Mobile-Phone Dispatch of Laypersons for CPR in Out-of-Hospital Cardiac Arrest. NEW ENGLAND JOURNAL OF MEDICINE. 2015;372(24):2316-25.
437. Zijlstra JA, Stieglis R, Riedijk F, Smeekes M, Van der Worp WE, Koster RW. Local lay rescuers with AEDs, alerted by text messages, contribute to early defibrillation in a Dutch out-of-hospital cardiac arrest dispatch system. RESUSCITATION. 2014;85(11):1444-9.

438. Hasselqvist-Ax I, Riva G, Herlitz J, Rosenqvist M, Hollenberg J, Nordberg P, et al. Early Cardiopulmonary Resuscitation in Out-of-Hospital Cardiac Arrest. New England Journal of Medicine. 2015;372(24):2307-15.
439. Perkins GD, Handley AJ, Koster RW, Castrén M, Smyth MA, Olasveengen T, et al. European Resuscitation Council Guidelines for Resuscitation 2015: Section 2. Adult basic life support and automated external defibrillation. Resuscitation. 2015;95:81-99.
440. Pijls RWM, Nelemans PJ, Rahel BM, Gorgels APM. A text message alert system for trained volunteers improves out-of-hospital cardiac arrest survival. Resuscitation. 2016;105:182-7.
441. Brooks SC, Simmons G, Worthington H, Bobrow BJ, Morrison LJ. The PulsePoint Respond mobile device application to crowdsource basic life support for patients with out-of-hospital cardiac arrest: Challenges for optimal implementation. Resuscitation. 2016;98:20-6.
442. Sasson C, Rogers MA, Dahl J, Kellermann AL. Predictors of survival from out-of-hospital cardiac arrest: a systematic review and meta-analysis. Circ Cardiovasc Qual Outcomes. 2010;3(1):63-81.
443. Berglund E, Claesson A, Nordberg P, Djärv T, Lundgren P, Folke F, et al. A smartphone application for dispatch of lay responders to out-of-hospital cardiac arrests. Resuscitation. 2018;126:160-5.
444. Caputo ML, Muschietti S, Burkart R, Benvenuti C, Conte G, Regoli F, et al. Lay persons alerted by mobile application system initiate earlier cardio-pulmonary resuscitation: a comparison with SMS-based system notification. Resuscitation. 2017;114:73-8.
445. Monsieurs KG, Nolan JP, Bossaert LL, Greif R, Maconochie IK, Nikolaou NI, et al. European resuscitation council guidelines for resuscitation 2015: section 1. Executive summary. Resuscitation. 2015;95:1-80.
446. Rumsfeld JS, Brooks SC, Aufderheide TP, Leary M, Bradley SM, Nkonde-Price C, et al. Use of Mobile Devices, Social Media, and Crowdsourcing as Digital Strategies to Improve Emergency Cardiovascular Care. Circulation. 2016;134(8):e87-e108.
447. Berg KM, Cheng A, Panchal AR, Topjian AA, Aziz K, Bhanji F, et al. Part 7: Systems of Care: 2020 American Heart Association Guidelines for Cardiopulmonary Resuscitation and Emergency Cardiovascular Care. Circulation. 2020;142(16_suppl_2):S580-S604.
448. Greif R, Bhanji F, Bigham BL, Bray J, Breckwoldt J, Cheng A, et al. Education, Implementation, and Teams: 2020 International Consensus on Cardiopulmonary Resuscitation and Emergency Cardiovascular Care Science With Treatment Recommendations. Circulation. 2020;142(16_suppl_1):S222-S83.
449. Semeraro F, Greif R, Böttiger BW, Burkart R, Cimpoesu D, Georgiou M, et al. European resuscitation council guidelines 2021: systems saving lives. Resuscitation. 2021;161:80-97.
450. Sarkisian L, Mickley H, Schakow H, Gerke O, Jørgensen G, Larsen ML, et al. Global positioning system alerted volunteer first responders arrive before emergency medical services in more than four out of five emergency calls. Resuscitation. 2020;152:170-6.
451. Scquizzato T, Pallanch O, Belletti A, Frontera A, Cabrini L, Zangrillo A, et al. Enhancing citizens response to out-of-hospital cardiac arrest: a systematic review of mobile-phone systems to alert citizens as first responders. Resuscitation. 2020;152:16-25.
452. Derkenne C, Jost D, Roquet F, Dardel P, Kedzierewicz R, Mignon A, et al. Mobile smartphone technology is associated with out-of-hospital cardiac arrest survival improvement: the first year “Greater Paris Fire Brigade” experience. Academic Emergency Medicine. 2020;27(10):951-62.
453. Lyznicki JM, Williams MA, Deitchman SD, Howe JP, 3rd. Inflight medical emergencies. Aviat Space Environ Med. 2000;71(8):832-8.
454. Matinrad N, Granberg TA, Angelakis V. Modeling uncertain task compliance in dispatch of volunteers to out-of-hospital cardiac arrest patients. Comput Ind Eng. 2021;159(C):15.
455. Folke F, Andelius L, Gregers MT, Hansen CM. Activation of citizen responders to out-of-hospital cardiac arrest. CURRENT OPINION IN CRITICAL CARE. 2021;27(3):209-15.
456. Metelmann C, Metelmann B, Kohnen D, Brinkrolf P, Andelius L, Böttiger BW, et al. Smartphone-based dispatch of community first responders to out-of-hospital cardiac arrest - statements from an international consensus conference. Scandinavian Journal of Trauma, Resuscitation and Emergency Medicine. 2021;29(1):29.
457. Bland J, Altman D. STATISTICAL METHODS FOR ASSESSING AGREEMENT BETWEEN TWO METHODS OF CLINICAL MEASUREMENT. The Lancet. 1986;327(8476):307-10.

458. Agatston AS, Janowitz WR, Hildner FJ, Zusmer NR, Viamonte M, Detrano R. Quantification of coronary artery calcium using ultrafast computed tomography. Journal of the American College of Cardiology. 1990;15(4):827-32.
459. Germano G, Kiat H, Kavanagh PB, Moriel M, Mazzanti M, Su H-T, et al. Automatic quantification of ejection fraction from gated myocardial perfusion SPECT. Journal of Nuclear Medicine. 1995;36(11):2138-47.
460. Critchley LAH, Critchley JAJH. A meta-analysis of studies using bias and precision statistics to compare cardiac output measurement techniques. Journal of clinical monitoring and computing. 1999;15(2):85-91.
461. DePuey EG, Nichols K, Dobrinsky C. Left ventricular ejection fraction assessed from gated technetium-99m-sestamibi SPECT. J Nucl Med. 1993;34(11):1871-6.
462. Devereux RB, Pickering TG, Harshfield GA, Kleinert HD, Denby L, Clark L, et al. Left ventricular hypertrophy in patients with hypertension: importance of blood pressure response to regularly recurring stress. Circulation. 1983;68(3):470-6.
463. Alderman MH, Ooi WL, Madhavan S, Cohen H. Blood pressure reactivity predicts myocardial infarction among treated hypertensive patients. Journal of Clinical Epidemiology. 1990;43(9):859-66.
464. Prisant LM, Bottini PB, Carr AA. Clinical utility of ambulatory blood pressure monitoring in target organ complications and equipment choices. J Clin Pharmacol. 1992;32(7):620-6.
465. Burker EJ, Fredrikson M, Rifai N, Siegel W, Blumenthal JA. Serum Lipids, Neuroendocrine, and Cardiovascular Responses to Stress in Men and Women with Mild Hypertension. Behavioral Medicine. 1994;19(4):155-61.
466. Lum DP, Coel MN. Comparison of automatic quantification software for the measurement of ventricular volume and ejection fraction in gated myocardial perfusion SPECT. Nucl Med Commun. 2003;24(3):259-66.
467. Knollmann FD, Helmig K, Kapell S, Hummel M, Bocksch W, Hetzer R, et al. Coronary artery calcium scoring: diagnostic accuracy of different software implementations. Invest Radiol. 2003;38(12):761-8.
